# Predicting Antigen‐Specificities of Orphan T Cell Receptors from Cancer Patients with TCRpcDist

**DOI:** 10.1002/advs.202405949

**Published:** 2024-08-19

**Authors:** Marta A. S. Perez, Johanna Chiffelle, Sara Bobisse, Francesca Mayol‐Rullan, Marine Bugnon, Maiia E. Bragina, Marion Arnaud, Christophe Sauvage, David Barras, Denarda Dangaj Laniti, Florian Huber, Michal Bassani‐Sternberg, George Coukos, Alexandre Harari, Vincent Zoete

**Affiliations:** ^1^ Department of Oncology Ludwig Institute for Cancer Research Lausanne Branch Lausanne University Hospital (CHUV) and University of Lausanne (UNIL) Agora Cancer Research Center Lausanne CH‐1005 Switzerland; ^2^ Molecular Modeling Group SIB Swiss Institute of Bioinformatics University of Lausanne Quartier UNIL‐Sorge, Bâtiment Amphipole Lausanne CH‐1015 Switzerland; ^3^ Center for Cell Therapy CHUV‐Ludwig Institute Lausanne CH‐1011 Switzerland; ^4^ Department of Oncology Immuno‐Oncology Service Lausanne University Hospital Lausanne CH‐1011 Switzerland

**Keywords:** deorphanization, epitope specificity, specificity prediction, t cell receptors (TCRs), tcr clustering, tumor antigens

## Abstract

Approaches to analyze and cluster T‐cell receptor (TCR) repertoires to reflect antigen specificity are critical for the diagnosis and prognosis of immune‐related diseases and the development of personalized therapies. Sequence‐based approaches showed success but remain restrictive, especially when the amount of experimental data used for the training is scarce. Structure‐based approaches which represent powerful alternatives, notably to optimize TCRs affinity toward specific epitopes, show limitations for large‐scale predictions. To handle these challenges, TCRpcDist is presented, a 3D‐based approach that calculates similarities between TCRs using a metric related to the physico‐chemical properties of the loop residues predicted to interact with the epitope. By exploiting private and public datasets and comparing TCRpcDist with competing approaches, it is demonstrated that TCRpcDist can accurately identify groups of TCRs that are likely to bind the same epitopes. Importantly, the ability of TCRpcDist is experimentally validated to determine antigen specificities (neoantigens and tumor‐associated antigens) of orphan tumor‐infiltrating lymphocytes (TILs) in cancer patients. TCRpcDist is thus a promising approach to support TCR repertoire analysis and TCR deorphanization for individualized treatments including cancer immunotherapies.

## Graphical abstract

TCRpcDist is a novel and structure‐based algorithm for calculating T‐Cell Receptors (TCRs) physico‐chemical Distances. Leveraging metrics associated with molecular interactions performed by key residues within the TCR, TCRpcDist demonstrates unparalleled efficacy in determining antigen specificities of orphan tumor‐infiltrating lymphocytes in cancer patients—a critical endeavor in contemporary immunotherapies.

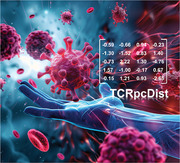

## 1 Introduction

T cell receptors (TCRs) orchestrate cellular immunity by recognizing peptide antigens presented by the major histocompatibility complex (pMHC).^[^ ^1^ ^]^ Although the principle of the TCR engagement with its cognate pMHC is well‐established,^[^ ^2^ ^]^ the prediction of which TCRs are accountable for triggering an immune response remains extremely difficult. This challenge is explained, in part, by the vast diversity of TCRs, with ≈10^20^ distinct TCRs generated through the V(D)J recombination process.^[^ ^3^ ^]^ Furthermore, the binding between TCRs and tumor epitopes is of relatively low‐affinity^[^ ^4^ ^]^ and is degenerate, meaning that several different TCRs can recognize the same antigen while at the same time one given TCR can recognize distinct antigens. Despite recent sequence‐ and structure‐based computational advances,^[^ ^5^, ^6^ ^]^ the unambiguous prediction of TCR‐pMHC pairing, from pools of thousands of candidates, remains a daunting task.^[^ ^7^ ^]^

Recent computational studies demonstrated that common patterns can be identified among TCR sequences interacting with the same epitope,^[^ ^8^, ^9^, ^10^, ^11^ ^]^ opening the road to an in silico characterization of the specificity, diversity and complexity of TCR repertoires.^[^ ^3^, ^12^ ^]^ Such approaches are already widely employed.^[^ ^13^, ^14^, ^15^ ^]^

Analysis of TCR sequences and TCR‐pMHC 3D structures led Glanville et al. in 2017^[^ ^8^ ^]^ to the observation that determining possible pMHC contact sites in complementary determining regions 3, CDR3s, notably in CDR3β, would offer an opportunity to cluster TCRs with a high probability of sharing the same specificity. Based on this assumption, the authors developed the GLIPH program (grouping of lymphocyte interactions by paratope hotspots) to cluster TCRs based on global and local TCR sequence similarity. When benchmarking GLIPH on a training set of 2068 unique sequences spanning eight pMHC specificities, the authors found that by combining local and global similarity 14% of the TCRs were clustered, of which 94% were correctly grouped with other TCRs of common specificity. Such an approach could be used to cluster TCRs that recognize the same epitope and to predict their HLA restriction. However, GLIPH loses efficiency and accuracy when more than 10′000 TCRs are analyzed. To circumvent this problem, Huang et al. developed GLIPH2 in 2020, which can process millions of TCR sequences.^[^ ^16^ ^]^

Dash et al. in 2017^[^ ^9^ ^]^ defined a distance measure on the space of TCRs, TCRdist, allowing clustering and visualization of repertoire diversity. This quantitative measure of similarity between paired αβ TCRs was obtained by listing the residues belonging to the CDR1, 2, and 3 loops, as well as an additional variable loop between CDR2 and CDR3, and by computing a similarity‐weighted mismatch distance defined based on the BLOSUM62 substitution matrix, with a gap penalty to capture variations in the length of the CDRs. Of note, a higher weight was given to the CDR3 sequence in view of its prominent role in epitope binding. This distance can then be calculated for each possible TCR pairs belonging to a given repertoire, generating a so‐called distance matrix. The latter can be used for TCRs clustering, or the construction of hierarchical distance trees to analyze the diversity and complexity of the TCR repertoire. In 2021, Mayer‐Blackwell et al.^[^ ^14^ ^]^ used a new version of TCRdist, TCRdist3,^[^ ^17^ ^]^ to guide the formation of meta‐clonotypes (i.e., groups of TCRs with biochemically similar CDRs that likely share antigen recognition) optimized for biomarker development. TCRdist3 brings new flexibility to distance‐based repertoire analysis, allowing customization of the distance metric, analysis of δγ TCRs, and at‐scale computation with sparse data representations and parallelized calculations.

In 2019, Ostmeyer and colleagues^[^ ^10^ ^]^ introduced an approach consisting in feeding machine learning techniques, based on logistic regressions, with biophysicochemical descriptors of the TCR interface for analyzing immune repertoires of several patients, with the objective to identify differences between TCRs in normal and tumor tissues. In this approach, the biophysicochemical characteristics of sliding windows of 4 consecutive residues of CDR3β (i.e., the so called 4‐mers), excluding the first 4 and last 3 residues, were described using five Atchley factors^[^ ^18^ ^]^ that encode for codon diversity, secondary structure, molecular size, polarity, and electrostatic charge of the residues. The method identified a short list of preferred values for these descriptors at key positions in TCRs present in the tumor, which permitted the identification of disease‐associated TCRs. Although this approach must be retrained for each set of TCRs studied and is restricted to CDR3β only (which limits its predictive ability), this type of sequence‐based “property”‐based approach could circumvent some of the drawbacks of purely sequence‐based analysis, such as the need to have very large numbers of disease‐associated TCR sequences available for training, and the possibility of detecting potential antigen‐binding TCRs even if their sequences differ from those that have been previously encountered.

Also in 2019, Lanzarotti et al. developed a model for prediction of TCR targets based on similarity to a database of TCRs with known pMHC.^[^ ^19^ ^]^ They showed increased predictive ability by focusing on CDRs rather than the full length TCR protein sequences, by incorporating information from paired α and β chains, and by integrating information for all 6 CDR loops rather than just CDR3. Additionally, they demonstrated that the inclusion of structural information in the model improved, consistently yet modestly, the accuracy of the epitope prediction, in particular in situations where no sequence with high similarity was available in the TCR database. They foresaw a promising advancement as TCR structural modeling tools improved in accuracy^[^ ^20^, ^21^, ^22^ ^]^ and the repository of available TCR 3D structures for modeling templates expanded.^[^ ^23^ ^]^

In 2021, Ehrlich introduced SwarmTCR, a method that combines sequence‐based approach with structural information.^[^ ^24^ ^]^ SwarmTCR uses CDR sequences to predict TCR specificity using a nearest‐neighbor approach. The approach works by optimizing the weights of the individual CDR regions to maximize classification performance, a knowledge taken from TCR‐pMHC structures.^[^ ^25^ ^]^ CDRs receive varying weightings based on the presented peptide and the MHC type to accurately represent the TCR's contribution to pMHC binding. SwarmTCR showed comparable performance to TCRdist when using TCR sequence information from single cell and bulk data recognizing 5 different pMHCs.^[^ ^24^ ^]^ The performance, robustness and generalizability of this approach was highly dependent on the training data and it has therefore proved difficult to extend its use to pMHCs lacking a large number of known specific TCRs.

Approaches predicting TCR‐pMHC binding based on their 3D structure and on force‐field‐based modeling have already been investigated.^[^ ^26^, ^27^ ^]^ Although physics‐based Molecular Modelling approaches at the atom scale have been proven powerful to optimize the affinity of a TCR toward a specific epitope, they are limited by calculation speeds and therefore can only be applied to a limited number of TCRs. Recently Lin et al. introduced RACER (Rapid Coarse‐Grained Epitope TCR), a pairwise energy model capable of rapidly assessing TCR‐peptide affinity for large‐scale HLA‐matched repertoires.^[^ ^27^ ^]^ RACER applies supervised machine learning to distinguish strong from weak TCR‐pMHC pairs, with fixed MHC. The trained parameters further enable a physical interpretation of the interaction patterns encoded in the TCR. When structural data for a specific TCR‐pMHC pair is unavailable, TCR‐pMHC models based on TCR‐pMHC experimental structures are built. Even though this approach depends on the training data and that pairwise interactions are only one of several factors influencing epitope recognition, this tool can be used to understand general questions regarding TCR and relevant antigen landscape. However, it is challenging to use this approach for TCR and pMHC pairing from a pool of thousands of candidates, since i) this would require making a model for all possible TCR‐pMHC combinations including structural relaxation, and because ii) the scope would be limited by the fact that the approach requires treating the different alleles separately and the TCR‐pMHC structure of the allele of interest has to exist on Protein Data Bank (PDB).^[^ ^25^, ^28^ ^]^

Building on the previously described methods and overcoming their constraints, we developed TCRpcDist (TCRs PhysicoChemical Distances), an innovative and fast approach that allows TCR clustering by analyzing the biophysicochemical properties of the relevant 4‐mer residue motifs of CDR1α, CDR2α, CDR3α, CDR1β, CDR2β, and CDR3β. TCRpcDist was compared with other existing approaches and further validated using public and private CD8+ TCR datasets (class I MHC interactions). We report that TCRpcDist identifies: 1) TCRs clusters that are likely to bind the same known epitopes and 2) the most probable epitope targeted by an orphan TCR from a pool of pMHCs for which TCR binders are known, ultimately resulting in the successful deophanization of multiple viral and tumor‐specific TCRs.

## 2 Results

**Figure 1:**
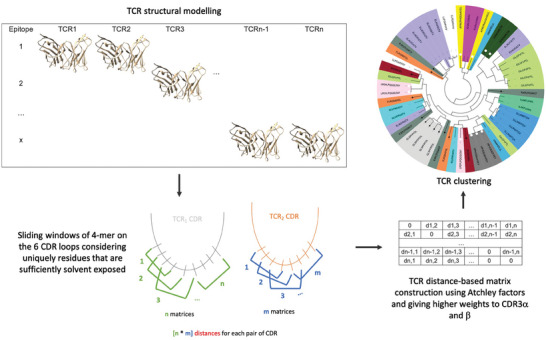
Representative scheme for the clustering pipeline used in TCRpcDist‐3D. The clustering pipeline consists of four main steps. First, all possible sliding windows of 4 residues that constitute the so‐called 4‐mer subunits are identified. The CDR residues that cannot directly contact the peptide, as determined by their solvent accessibility in the structural models, can be excluded from the process. Next, each 4‐mer subunit is converted into a biophysicochemical representation using 5 Atchley factors. For each CDR of a pair of TCRs, all the *n* 4‐mer motifs that are possible to construct from the first TCR with all the *m* possible 4‐mer motifs of the second TCR are compared. This results in *n* × *m* matrix comparisons for each CDR for each pair of TCRs. The matrix comparisons are performed via a Manhattan distance score normalized over the maximum possible distance. This score ranges from 0, for 4‐mers sharing the same biophysicochemical properties, to 1, for 4‐mers that have the highest difference in biophysicochemical properties. The method was developed using TCR‐pMHC PDB structures before being tested for use with TCR homology models for broader applications. The clustering accuracy is found maximal when a weighting of ≈30% is applied to the subset of amino acids in CDR3α or CDR3β and a weighting of 10% are given to the subset of amino acids in CDR1α, CDR2α, CDR1β, or CDR2β, respectively. The clustering accuracy is better when, together with the weighting factors, only residues sufficiently exposed to the solvent, thus potentially able to contribute to the pMHC binding interface, are part of the 4‐mers.

TCRpcDist is a novel and fast approach that calculates distances between TCRs using a metric related to the physico‐chemical properties of the most important residues of these receptors. The clustering pipeline consists of four main steps, (**Figure** **1**). First, all possible sliding windows of 4 residues that constitute the so‐called 4‐mer subunits are identified on the CDRs of the TCR. The CDR residues that cannot directly contact the peptide, as determined by their solvent accessibility in the structural models, can be excluded from the process. Next, each 4‐mer subunit is converted into a biophysicochemical representation using 5 Atchley factors. For each CDR of a pair of TCRs, all the *n* 4‐mer motifs that are possible to construct from the first TCR with all the *m* possible 4‐mer motifs of the second TCR are compared. This results in *n* × *m* matrix comparisons for each CDR for each pair of TCRs. The matrix comparisons are performed via a Manhattan distance score normalized over the maximum possible distance. This score ranges from 0, for 4‐mers sharing the same biophysicochemical properties, to 1, for 4‐mers that have the highest difference in biophysicochemical properties. The method was developed using TCR‐pMHC PDB structures before being tested for use with TCR homology models for broader applications. The clustering accuracy is found maximal when a weighting of ≈30% is applied to the subset of amino acids in CDR3α or CDR3β and a weighting of 10% are given to the subset of amino acids in CDR1α, CDR2α, CDR1β, or CDR2β, respectively. The clustering accuracy is better when, together with the weighting factors, only residues sufficiently exposed to the solvent, thus potentially able to contribute to the pMHC binding interface, are part of the 4‐mers. (Figure 1). We propose 3 different versions of the approach: i) using only the TCR CDR3β loop sequence, TCRpcDist^CDR3β^; ii) using all the 6 CDR loops sequences, TCRpcDist^6CDRs^, and iii) using all the 6 CDR loops together with their solvent accessibility information in their experimental or modeled 3D structures, TCRpcDist‐3D. The ability of the three different versions of the approach to cluster TCR repertoire is extensively assessed and compared with state‐of‐the‐art competitors. Finally, TCRpcDist‐3D is applied to the TCR repertoires of cancer patients resulting in the identification and experimental validation of cognate pMHC for 11 orphan TCRs.

### 2.1 The Maximal Clustering Efficiency of TCRpcDist is Obtained When Taking the Solvent Accessibility of the CDRs Residues into Account

TCRpcDist approach was developed using 54 TCRs recognizing 16 distinct known pMHC taken from experimentally determined structures of class I TCR‐pMHC complexes stored in the PDB (Table S1, Supporting Information). Given that the 3D version of our approach considers aspects of TCR‐pMHC 3D interactions, we developed TCRpcDist using a dataset of TCRs for which the TCR‐pMHC 3D structures are known. Consequently, a set of 54 non‐redundant TCRs was the largest available set for developing our approach as of June 2020.

**Figure 2:**
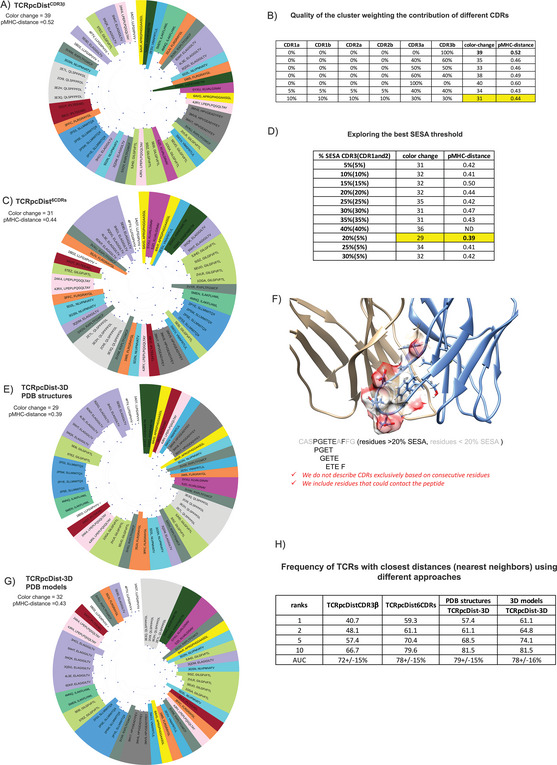
TCRpcDist clustering TCRs and correlating with their specificity. A) shows hierarchical clustering of a set of 54 TCRs recognizing 16 different pMHC using the Atchley‐based distance considering only sliding windows of 4 consecutive residues of the CDR3β. After clustering, each TCR is colored according to the pMHC it binds. The sequence of the bound peptide is also given; B) quality of the cluster as measured by the number of color changes and the pMHC‐distance, for diverse weightings of the contributions of the various CDRs. The maximal clustering efficiency is highlighted in yellow and obtained when each CDR3s contribute by 30% and each of the remaining CDRs by 10% to the distance calculation; C) shows the hierarchical clustering of a set of 54 TCRs recognizing 16 different pMHC using the Atchley‐based distance considering all 6 TCR CDRs (i.e., CDR1α, CDR2α, CDR3α, CDR1β, CDR2β, and CDR3β). After clustering, each TCR is colored according to the pMHC it binds. The sequence of the bound peptide is also given; D) exploring the best nSESA threshold. The clustering efficiency as measured by the number of color changes and the pMHC‐distance is maximal when residues with nSESA < 5% in CDRs 1 and 2 and residues with nSESA < 20% in CDRs 3 are excluded from the distance calculation; E) shows hierarchical clustering of a set of 54 TCRs recognizing 16 different pMHC using the Atchley‐based distance considering all 6 TCR CDRs (i.e., CDR1α, CDR2α, CDR3α, CDR1β, CDR2β, and CDR3β), as well as residues buriedness calculated on the PDB structures. After clustering, each TCR is colored according to the pMHC it binds. The sequence of the bound peptide is also given; F) illustrates how solvent exposed residues are included in the distance calculation while buried residues are excluded (The TCR structure corresponds to PDB ID 4JRX); G) shows hierarchical clustering of a set of 54 TCRs recognizing 16 different pMHC using the Atchley‐based distance considering all 6 TCR CDRs (i.e., CDR1α, CDR2α, CDR3α, CDR1β, CDR2β, and CDR3β), as well as residues buriedness calculated on 3D models created by our pipeline that models TCRs from sequences. After clustering, each TCR is colored according to the pMHC it binds. The sequence of the bound peptide is also given; H) table shows how often a TCR with the same specificity is found in the top 1, 2, 5 and 10 TCRs with the closest distances using 4 versions of TCRpcDist (using just CDR3β, using all CDRs, using all CDRs + nSESA (residues buriedness) taken from PDB structures and using all CDRs + nSESA (residues buriedness) taken from 3D models constructed by our pipeline to model sequences). Area Under ROC curve (AUC) as a measure of accuracy and respective standard deviation is also presented.

Our analysis was first carried out with TCRpcDist^CDR3β^ assuming that the CDR3β loop of a TCR makes most of the interactions with the peptide presented by the pMHC. The clustering results, illustrated in **Figure** **2**A, were determined solely based on TCRpcDist^CDR3β^, without consideration of the corresponding cognate pMHCs. The coloring according to the TCR specificity was applied afterward, to display if similar TCRs indeed bind identical pMHC. We observed that, even though only CDR3β residues were used to calculate distances, clusters of receptors binding the same pMHC tend to spontaneously form. We measured the number of times the color changed between two successive nodes in the hierarchical clustering tree (Figure 2A) as a qualitative measure of the clustering efficacy, starting from the upper node and turning clockwise. Thirty‐nine color changes were observed, which was an encouraging result since a random clustering provided an average of 51 color changes (*p* < 0.0001). The quality of the clustering was also determined by a more quantitative metric, pMHC‐distance, defined as the average branch length distance between all possible pairs of TCR nodes that recognize the same pMHC. The lower this average, the closer the TCRs binding the same pMHC are grouping in this hierarchical clustering. The average pMHC‐distance was 0.52 for the hierarchical clustering generated by TCRpcDist^CDR3β^ (**Table** **1**) while a random clustering provided a pMHC‐distance of 0.94 (*p* < 0.0001).

**Table 1:** Average branch length distance and number of clusters (#n) per peptide in the hierarchical clustering tree for the PDB set considering different versions of TCRpcDist to calculate the distance: considering only CDR3β TCRpcDist^CDR3β^, or all CDRs, TCRpcDist6^CDRs^, and all CDRs+ nSESA based on experimental 3D structure (PDB) and all CDRs+ nSESA based on 3D structural models, TCRpcDist‐3D. Each peptide contributes with the same weight (1/16) to the final pMHC‐distance, to prevent this measure to be biased by the most frequent pMHCs. The number of TCRs per peptide is also presented, in column1.

n. TCRS	peptide	TCRpcDist‐CDR3β		TCRpcDIist‐6CDRs		TCRpcDist‐3D (PDB structures)		TCRpcDist‐3D (3D models)	
		pMHC‐distance	n. clusters	pMHC‐distance	n. clusters	pMHC‐distance	n. clusters	pMHC‐distance	n. clusters
2	APRGPHGGAASGL	0.90	#2	0.52	#2	0.49	#2	0.66	#2
2	ASNENMETM	0.68	#2	0.11	#1	0.14	#1	0.16	#1
8	ELAGIGILTV	0.67	#5	0.60	#3	0.42	#2	0.47	#3
3	FLRGRAYGL	0.83	#3	0.74	#3	0.71	#2	0.73	#3
7	GILGFVFTL	0.66	#5	0.64	#3	0.48	#3	0.55	#3
3	HPVGEADYFEY	0.00	#1	0.00	#1	0.00	#1	0.00	#1
2	ILAKFLHWL	0.00	#1	0.16	#1	0.06	#2	0.12	#2
2	IPLTEEAEL	0.12	#1	0.69	#2	0.85	#2	0.92	#2
2	KLVALGINAV	0.76	#2	0.17	#1	0.18	#1	0.19	#1
3	LLFGXPVYV	0.45	#3	0.57	#2	0.33	#2	0.26	#2
3	LPEPLPQGQLTAY	0.72	#3	0.47	#2	0.58	#2	0.55	#2
3	NLVPMVATV	0.77	#3	0.54	#2	0.64	#2	0.64	#3
4	QLSPFPFDL	0.00	#1	0.22	#1	0.20	#1	0.12	#1
4	RXPLTFGWCF	0.85	#4	0.64	#4	0.65	#4	0.76	#4
4	SLLMWITQX	0.00	#1	0.10	#1	0.03	#1	0.09	#1
2	VMAPRTLIL	0.96	#2	0.91	#2	0.53	#2	0.70	#2
–	Average	0.52	–	0.44	–	0.39	–	0.43	–
–	STDEV	0.37	–	0.28	–	0.27	–	0.29	–

Our analysis was afterward carried out by considering the contributions of all six CDR loops, i.e., CDR1α, CDR2α, CDR3α, CDR1β, CDR2β, and CDR3β in our TCR distance definition, TCRpcDist^6CDRs^. The quality of the resulting clustering as measured by the color change and the pMHC‐distance in the hierarchical clustering tree was optimized by weighting the contribution of the different CDRs (Figure 2B). The clustering relevance was maximal when weighting values of 30% were applied to the subset of amino acids in CDR3α or CDR3β and of 10% to the subset of amino acids in CDR1α, CDR2α, CDR1β, or CDR2β (Figure 2B). To avoid overfitting the model on the developmental set, we did not perform a full sampling of the effects of the weights to optimize their values. The hierarchical clustering using all CDRs (Figure 2C) was much better at grouping together TCRs that bind the same pMHC, with only 31 color changes (*p* < 0.0001 compared to random), compared to 39 when considering only CDR3β (p‐value < 0.0001, Figure 2A). Concomitantly, the pMHC‐distance decreased to 0.44, compared to 0.52 when considering only CDR3β (p‐value < 0.0001, Table 1). For example, the 3 TCRs recognizing the peptide LPEPLPQGQLTAY, with PDB codes 4JRY, 4JRX, and 2AK4 were not clustered when based exclusively on CDR3β (pink slices were separated in Figure 2A). The clustering efficiency improved when considering all the CDRs, with 2 TCRs (2AK4 and 4JRX) out of 3, now grouped together (Figure 2B). 2AK4 and 4JRX clustered together using all CDRs as they share the same TRAV, CDR3α, and TRBV genes, i.e., same CDR1α, CDR2α, CDR3α, CDR1β, and CDR2β (Table S1, Supporting Information). Of note, when working with bulk sequencing data, αβ pairing information is not available and, despite the lower accuracy of the approach using uniquely CDR3β information, this method can still be effectively used for clustering purposes, with much better results than at random (p‐value < 0.0001). The relevance of the clustering approach using exclusively CDR3β residues was already shown in a previous study.^[^ ^5^ ^]^

Finally, our analysis was performed considering the solvent accessibility of the 6 CDRs loops’ residues in the TCR 3D structure, TCRpcDist‐3D. CDRs residues buried into the TCR structure were not considered in the TCR distance calculation as they were not available for the pMHC interaction. Normalized Solvent Excluded Surface Area (nSESA) for all residues in the 6 CDRs were calculated on the experimental 3D structures of the TCRs (SESA values per TCR per CDR residue can be seen in Data S1 (Supporting Information) and the clustering efficiency was tested on different SESA thresholds (Figure 2D). Then, the residues of CDRs 1 and 2 (α and β), with a nSESA > 5% were considered for TCR distance calculation, while a threshold of 20% was applied for the two CDR3s (α and β), this combination of parameters giving the maximum clustering efficiency. Accordingly, the distances between CDRs were calculated using sliding windows of 4 consecutive solvent‐exposed residues, and not necessarily consecutive residues. Indeed, Ostmeyer et al. already showed that the residues interacting pMHC are not necessarily consecutive in the TCR structure.^[^ ^10^ ^]^ Taking the buriedness of the residues into account enhanced the quality of the clustering (Figure 2E). The number of color changes in the hierarchical clustering tree reached 29 and the pMHC‐distance decreased to 0.39 (*p* < 0.0001 compared to random and compared with previous versions of the approach) (see Table 1). Noticeably, we observed in Figure 2F that the 4^th^ residue of the CDR3β i.e., Pro in this example, excluded from the distance calculation in TCRpcDist^6CDRs^, is sufficiently solvent exposed and should be considered for the analysis of the TCR distance. Indeed, the 3D structure of the complex TCR‐pMHC PDB ID 4JRX confirmed that the Pro residue contacts the peptide (Figure S1, Supporting Information). This illustrated that approaches that systematically get rid of the first 4 and the last 3 residues from the CDR loops,^[^ ^10^, ^15^ ^]^ since they are supposed to be likely buried, might in fact accidentally remove residues relevant for binding. TCRpcDist‐3D avoided this approximation by computing the nSESA for all CDR residues to determine their solvent accessibility and excluded the buried residues (nSESA < 20%) from the distance calculation.

Since CDR loops of TCR are extremely flexible and since we will be using TCR 3D models instead of X‐ray structures in real case applications, we clustered these TCRs using structural models instead of experimental structures (Figure 2G). TCR models were constructed without using templates that share the same genes as the query TCR to make the exercise less obvious. We observed a much better clustering efficiency for several pMHC‐related TCRs when using nSESA from structural models compared to the version of the approach without any structural information (Table 1). The hierarchical clustering tree showed that, for example, the TCRs recognizing the peptide ELAGIGILTV clustered much better when solvent accessibility was considered whether we were using structural models or experimental structures (purple slices more spread in Figure 2A,C and closer in Figure 2E,G). The average of the branch length distance between the nodes of ELAGIGILTV‐specific TCRs decreased from 0.67, to 0.60, to 0.47 and ultimately to 0.42 using respectively, TCRpcDist^CDR3β^, TCRpcDist^6CDRs^, TCRpcDist‐3D with 3D models and TCRpcDist‐3D with 3D structures (Table 1).

To further compare the different versions of the approach, we calculated how often a TCR with the same specificity was found in the top 1, 2, 5, and 10 closest TCRs (nearest neighbours) using different versions of TCRpcDist (Figure 2H). An increase of that frequency was observed at rank 10, progressing from 66.7% (TCRpcDistCDR3ß) to 79.6% (TCRpcDist^6CDRs^), p‐value < 0.001, and ultimately to 81.5% (TCRpcDist‐3D, using either 3D experimental or modeled structures), p‐value = 0.3. The increase is significant between TCRpcDistCDR3ß and TCRpcDist^6CDRs^ and continues between TCRpcDist^6CDRs^ to TCRpcDist‐3D, although less significantly. Thus, the inclusion of 3D structures provides added value for several specificities, although it is less efficient for others. This was in line with the improvement of the scores related to the hierarchical clustering trees, i.e., the number of color changes and the pMHC‐distance, as hierarchizations consider all the distances between all the possible pairs (here 54*54 distances). Interestingly, using TCRpcDist‐3D with models at rank 1, 2, 5, and 10, we found the right selectivity within the closest TCRs in 61.1%, 64.8%, 74.1%, and 81.5% of the cases, respectively, which was better than what was obtained when using experimental structures or even better than the TCRpcDist^6CDRs^ (Figure 2H). We observed that some TCRs with identical specificity and with high sequence identity were structurally closer in structural models than in experimental structures, explaining these improvements in the ranks.

In July 2024, in response to the increased number of TCRs deposited in the PDB, we reassessed our approach using a set of 96 TCRs that recognize 48 distinct known pMHCs. We observed that the weighting parameters and the solvent accessibility thresholds developed using 54 TCRs and 16 pMHC and employed in the current version of TCRpcDist remain valid. See Data S2 (Supporting Information).

### 2.2 Clustering Efficiency of TCRpcDist Using a Private Data Set of TCRs

**Figure 3:**
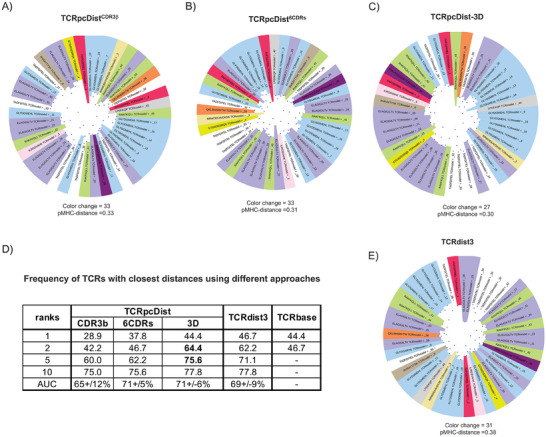
TCRpcDist clustering TCRs and correlating with their specificity using a private set of TCRs with known specificity. A) shows the hierarchical clustering of a test set of 45 TCRs recognizing 12 different pMHC using the Atchley‐based distance considering only sliding windows of 4 consecutive residues of the CDR3β. These TCRs were not used to choose the TCRpcDist parameters. After clustering, each TCR is colored according to the pMHC it binds. The sequence of the bound peptide is also given; B) shows the hierarchical clustering of a set of 45 TCRs recognizing 12 different pMHC using the Atchley‐based distance and considering all 6 TCR CDRs (i.e., CDR1α, CDR2α, CDR3α, CDR1β, CDR2β, and CDR3β). After clustering, each TCR is colored according to the pMHC it binds. The sequence of the bound peptide is also given; C) shows the hierarchical clustering of a set of 45 TCRs recognizing 12 different pMHC using the Atchley‐based distance, considering all 6 TCR CDRs (i.e., CDR1α, CDR2α, CDR3α, CDR1β, CDR2β, and CDR3β) as well as residues buriedness. After clustering, each TCR is colored according to the pMHC it binds. The sequence of the bound peptide is also given; D) table shows how often a TCR with the same specificity is found in the top 1, 2, 5 and 10 TCRs with the closest distances using the 3 versions of TCRpcDist, TCRbase (webserver: https://services.healthtech.dtu.dk/services/TCRbase‐1.0/ ) and TCRdist3^[^ ^14^ ^]^ approaches. AUC as a measure of accuracy and respective standard deviation is also presented; E) shows hierarchical clustering of a set of 45 TCRs recognizing 12 different pMHC using TCRdist3.^[^ ^14^ ^]^

The approach was then applied to a private set of 45 TCRs with 12 different known cognate pMHCs from 4 melanoma patients (Mel#1‐#4) (most described in Arnaud et al.,^[^ ^29^ ^]^ Table S2, Supporting Information). Since the experimental 3D structure of these TCRs is unknown, the buriedness of all the CDR residues was calculated based on TCR models. The hierarchical clustering trees for this assessment set were depicted in **Figure** **3** and as for the previous set, we measured the quality of the clustering by color change and pMHC‐distance. The number of color changes was 33 using TCRpcDist^CDR3β^ (Figure 3A), 33 using TCRpcDist^6CDRs^ (Figure 3B), and 27 with TCRpcDist‐3D (Figure 3C), which was much better than random (p‐value<0.0001 in all cases). The pMHC‐distance was 0.33, 0.31, and 0.30 for these three TCRpcDist variants, respectively. Once again, we observed an increase of the clustering efficiency using TCRpcDist‐3D, confirming the robustness of the approach, even though we were using TCR structural models instead of TCR experimental structures. Calculation of TCR loop conformations being stochastic, TCRpcDist‐3D is itself stochastic, contrarily to TCRpcDist which is deterministic. However, the variability on calculated TCRpcDist‐3D distances between several runs, starting from the same input, remains very limited (See Data S3, Supporting Information) and does not change the conclusions of TCR distance analysis.

Hierarchical clustering trees (Figure 3A–C) showed that, for example, the TCRs recognizing the peptide ELAGIGILTV (overrepresented in the set with 27% of all TCRs recognizing it, Table S2, Supporting Information) clustered much better with TCRpcDist‐3D (purple slices closer in Figure 3C). Nevertheless, even at this level of approximation (Figure 3C) they formed sub‐clusters instead of a single cluster. On the contrary, the TCRs recognizing the same peptide – also overrepresented in the PDB (17% of the TCRs, Table S1, Supporting Information) – formed mainly a single cluster when applying TCRpcDist‐3D either using PDB structures or models (Figure 2E,G). Sub‐clusters of ELAGIGILTV were formed in the present set due to a much higher TCR sequence variability. TCRs recognizing ELAGIGILTV in this set exhibited 10 different TRAV genes, 8 different TRBV genes, 18 different AA in CDR3α and 16 in CDR3β, while TCRs recognizing ELAGIGILTV in the PDB set showed only 2 different TRAV genes (87.5 % of the TCRs recognizing TRAV12‐2), 5 different TRBV genes and 12 different AA in CDR3α and β. The statistics regarding the different composition of the TCRs recognizing ELAGIGILTV are given in Table S3 (Supporting Information). Clustering this private set was challenging as it contained singleton TCRs and high sequence variability for TCRs recognizing the same peptide. Still the clustering efficiency was remarkable, especially for TCRpcDist‐3D. TCR pairs with the closest distance (top 1) shared the same specificity in 28.9%, 37.8%, and in 44.4% of the cases, respectively for TCRpcDist^CDR3β^, TCRpcDist^6CDRs^, and TCRpcDist‐3D (p‐values <0.0001) (Figure 3D). The predictive power of our approach was increased by considering the top 2, top 5 or the top 10 closest TCRs to a given TCR. Indeed, the specificity of a given TCR was shared by at least one of the 2, 5, and 10 top‐ranked TCRs in 42.2%, 60.0%, and 75% of the cases, respectively, when using TCRpcDist^CDR3β^ (p‐value < 0.0001). When using TCRpcDist^6CDRs^, the success increased to 46.7%, 62.2%, and 75.6%, and even reached 64.4%, 75.6%, and 77.8% for TCRpcDist‐3D (Figure 3D). The gain was impressive when comparing the data with and without 3D information (maximum gain of 22.2% points for rank 2 when comparing TCRpcDist^CDR3β^ with TCRpcDist‐3D). We note that this set contained singleton peptides for 6 TCRs, for which it was impossible to find a TCR pair with the same specificity, hence the maximum probability of finding a TCR pair was 86.7% and not 100%. We used structural models instead of experimental structures and once again the superior accuracy TCRpcDist‐3D was confirmed.

### 2.3 TCRpcDist‐3D Compares Favorably to State‐Of‐The‐Art Approaches

We applied one of the most renown and freely available method, TCRdist3,^[^ ^17^ ^]^ to analyze the diversity and complexity of the above mentioned private set, Figure 3E, and compared with TCRpcDist‐3D, Figure 3C. The private set of TCRs was not used to train either of these two approaches, thus ensuring an unbiased comparison. For TCRdist3 we observed a hierarchical clustering with 31 color changes and a pMHC‐distance of 0.38, while for TCRpcDist‐3D we obtained 27 color changes and a pMHC‐distance of 0.30. This significant difference in the number of color changes and pMHC‐distance values (*p* < 0.0001), indicated a slightly higher clustering efficiency for TCRpcDist‐3D on this set. To evaluate if we can use both TCRpcDist‐3D and TCRdist3 in a consensus way, and possibly generate a synergy between the two approaches, we combined TCRpcDist‐3D and TCRdist3 normalized distances (between 0 and 1), each approach contributing to 50% of the final new TCR distances. The corresponding hierarchical clustering (Figure S2, Supporting Information) presented 30 color changes and a pMHC‐distance of 0.36, which was better than TCRdist3 but worse than TCRpcDist‐3D, illustrating that there was no added value using the two approaches in this manner and in this case.

We also quantified the outcome of the similarity principle using TCRpcDist‐3D, TCRdist3 and TCRbase (web server: https://services.healthtech.dtu.dk/services/TCRbase‐1.0/), and analyzed the frequency with which the TCR with the smallest distance from a given one recognizes the same pMHC. Our findings indicated that all three approaches are valuable, albeit with slight differences in performance. TCRdist3 exhibited the highest success rate in pairing TCRs with the same specificity when considering the TCR with the closest distance (rank 1), achieving a success rate of 46.7%, compared to 44.4% for TCRpcDist‐3D and TCRbase 1.0 (Figure 3D). However, TCRpcDist‐3D performed better than the others when the two closest TCRs were considered (rank 2), with a success rate of 64.4%, compared to 62.2% for TCRdist3 and 46.7% for TCRbase 1.0. TCRpcDist‐3D outperformed TCRdist3 at rank 5, while all approaches exhibited similar success rate at rank 10 (Figure 3D). These results reflects with the better scores in terms of color changes and pMHC‐distance shown by TCRpcDist‐3D, as the hierarchical clustering trees represented the distances between all TCR pairs, and not just the closest TCR or a given rank.

Among the 12 different peptides present in our private set, only two (ELAGIGILTV and RAKFKQLL) are covered by pre‐trained models of the NetTCR‐2.2 webserver (https://services.healthtech.dtu.dk/services/NetTCR‐2.2/). Therefore, we investigated if NetTCR‐2.2 could accurately predict the specificity of each TCR in the private set, known to bind ELAGIGILTV and RAKFKQLL, by ranking these peptides highest among the 26 possibilities. For these two TCRs, we analyzed the frequency with which the peptides scored highest (rank 1 and rank 2) corresponded to the known binders and compared these results with the specificities inferred by the nearest neighbors (rank 1 and rank 2) using TCRpcDist‐3D, TCRbase, and TCRdist3 as described in the previous paragraph. Although the comparison conducted here with NetTCR‐2.2 was different, the objective remained the same: inferring the specificities of a given TCR. At rank 1, for the TCRs recognizing ELAGIGILTV, the success in predicting the correct specificity was lower for NetTCR‐2.2, with a correct prediction in 41.7% of cases, while the success rate was 50% for TCRpcDist‐3D, TCRdist3, and TCRbase. At rank 2, NetTCR‐2.2 maintained the same number of correct predictions, while all other approaches increased in accuracy, achieving 58.3% for TCRbase, 78.0% for TCRpcDist‐3D, and 83.3% for TCRdist3. The success rates were lower for all four approaches when analyzing the TCRs recognizing RAKFKQLL, with the following results at ranks 1 and 2, respectively: 20% and 40% for TCRpcDist‐3D, 20% and 20% for NetTCR‐2.2, 0% and 0% for TCRbase, and 40% and 60% for TCRdist3. TCRdist3 performed better for this subset and these two particular specificities at ranks 1 and 2, but not for the entire private set (Figure 3D) where TCRpcDist‐3D showed the same accuracy at rank 1 and outperformed TCRdist3 at rank 2. These results are discussed in Data S4 (Supporting Information).

**Figure 4:**
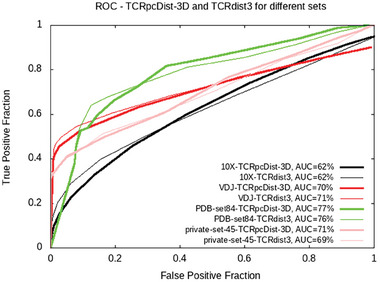
ROC curves computed using the TCRpcDist‐3D and TCRdist3^[^ ^9^, ^17^ ^]^ approaches for 4 independent sets: the private set of 45 TCRs, the set of 84 PDB structures, the 10X Genomics set comprising 1′956 TCRs and the VDJdb2022 comprising 8′128 TCRs covering 337 pMHC bound by a single TCR and 334 pMHC bound by at least two TCRs.

We further compared TCRpcDist‐3D and TCRdist3 using a standard metric of the classification success, the receiver operating characteristic curve (ROC), to assess the performance of the nearest‐neighbors distance. ROC curves were computed for 4 independent sets: a set of 84 experimental structures, the private set of 45 TCRs (Table S2, Supporting Information), the 10X Genomics set comprising 1′956 TCRs (see methods and Table S4, Supporting Information) and the VDJdb2022 comprising 8′128 TCRs and covering 337 pMHC bound by a single TCR and 334 pMHC bound by at least two TCRs (Table S5, Supporting Information). The area under these ROC curves (AUC) ranged between 62% and 67% for both TCRpcDist‐3D and TCRdist3 (**Figure** **4**). The overall accuracy of both predictors was satisfactory but not very high, notably due to the presence of TCRs with a single specificity that were impossible to pair and the presence of pMHCs recognized by only a few different TCRs. Globally TCRpcDist‐3D and TCRdist3 provided significantly similar results.

To test the predictive power for a given peptide, we did 6 cross‐validations by randomly allocating 30% of all TCRs specific for a pMHC as a reference. We tested how often the TCRs with the lowest distances recognize the same peptide when compared with the reference, and calculated sensitivity and specificity. For this analysis we took the 40 most represented peptides in VDJdb2022 (each peptide having at least 20 specific TCRs) and the average of the AUC was calculated together with its standard deviation for each given pMHC (**Table** **2**). Results showed that the efficacies of TCRpcDist‐3D and TCRdist3 are peptide‐dependent. TCRpcDist‐3D was better at predicting TCR specificities for certain peptides such as AVFDRKSDAK, NLVPMVATV, IVTDFSVIK, SPRWYFYYL, CINGVCWTV, RLRAEAQVK, and NQKLIANQF with AUC showing higher AUC (p‐value < 0.0001), while TCRdist3 was better at predicting specificities for certain peptides such as RLPGVLPRA, RAKFKQLL, PTDNYITTY, NYNYLYRLF, RAQAPPPSW, YLQPRTFLL, and LLWNGPMAV showing higher AUC (p‐value < 0.0001). The approaches yielded comparable results for the remaining 26 peptides evaluated.

**Table 2:** TCRpcDist‐3D versus TCRdist3: comparison of their predictive power for the 40 most represented peptides on VDJdb2022. Average of the area under receiver operating characteristic curves (AUC) and standard deviations are presented per approach and per peptide. The differences between the approaches are calculated and the peptide is highlighted in bold when the differences between the approaches are significant, with p‐values < 0.0001.

Peptide	TCRpcDist‐3D		TCRdist3		[AUC(TCRpcDist‐3D)]‐ [AUC(TCRdist3)]
	AUC	SD	AUC	SD	
AVFDRKSDAK	56.78%	0.08%	54.63%	0.08%	2.2%
NLVPMVATV	67.30%	0.12%	65.42%	0.12%	1.9%
IVTDFSVIK	65.86%	0.13%	64.06%	0.29%	1.8%
SPRWYFYYL	70.72%	0.25%	69.04%	0.19%	1.7%
CINGVCWTV	72.91%	0.20%	71.34%	0.35%	1.6%
RLRAEAQVK	68.26%	0.15%	67.24%	0.27%	1.0%
GPRLGVRAT	75.88%	0.23%	75.02%	0.24%	0.9%
HPVTKYIM	76.12%	0.19%	75.47%	0.17%	0.6%
RVAGDSGFAAY	77.79%	0.20%	77.15%	0.20%	0.6%
FTSDYYQLY	77.27%	0.22%	76.70%	0.27%	0.6%
NQKLIANQF	74.95%	0.16%	74.38%	0.12%	0.6%
RPHERNGFTVL	77.29%	0.27%	76.79%	0.10%	0.5%
QYIKWPWYI	76.55%	0.19%	76.06%	0.23%	0.5%
ATDALMTGF	72.47%	0.30%	72.02%	0.22%	0.4%
LTDEMIAQY	71.79%	0.16%	71.37%	0.15%	0.4%
ALWEIQQVV	77.26%	0.13%	76.88%	0.20%	0.4%
VYFLQSINF	76.74%	0.10%	76.43%	0.13%	0.3%
DATYQRTRALVR	73.47%	0.31%	73.24%	0.18%	0.2%
LLFGYPVYV	76.75%	0.13%	76.52%	0.13%	0.2%
RTLNAWVKV	76.68%	0.12%	76.51%	0.12%	0.2%
AYAQKIFKI	77.06%	0.11%	76.99%	0.08%	0.1%
FLYALALLL	80.27%	0.05%	80.26%	0.07%	0.0%
TPRVTGGGAM	98.46%	0.01%	98.47%	0.01%	0.0%
KSKRTPMGF	98.25%	0.01%	98.26%	0.01%	0.0%
LLYDANYFL	78.17%	0.13%	78.19%	0.14%	0.0%
RPPIFIRRL	77.68%	0.16%	77.92%	0.14%	‐0.2%
ALSKGVHFV	77.09%	0.12%	77.34%	0.13%	‐0.3%
YSEHPTFTSQY	77.26%	0.21%	77.51%	0.09%	‐0.3%
TTDPSFLGRY	68.52%	0.20%	69.11%	0.25%	‐0.6%
ELAGIGILTV	76.71%	0.12%	77.31%	0.23%	‐0.6%
RLPGVLPRA	75.94%	0.13%	76.61%	0.19%	‐0.7%
GLCTLVAML	70.41%	0.31%	71.31%	0.42%	‐0.9%
PTDNYITTY	77.42%	0.20%	78.39%	0.17%	‐1.0%
RAKFKQLL	65.34%	0.17%	66.32%	0.11%	‐1.0%
KLVALGINAV	77.56%	0.36%	78.54%	0.20%	‐1.0%
NYNYLYRLF	79.03%	0.15%	80.05%	0.13%	‐1.0%
RAQAPPPSW	77.22%	0.16%	78.28%	0.32%	‐1.1%
YLQPRTFLL	70.31%	0.30%	71.40%	0.17%	‐1.1%
GILGFVFTL	74.07%	0.59%	75.71%	0.55%	‐1.6%
LLWNGPMAV	72.44%	0.32%	76.52%	0.21%	‐4.1%

A comparison with the predictive model SwarmTCR, is presented in Data S4 (Supporting Information) further illustrating the competitiveness of TCRpcDist.

### 2.4 Applying Distance Thresholds on TCRpcDist‐3D Yields >90% Accurate Pairing TCRs of the Same Specificity

**Figure 5:**
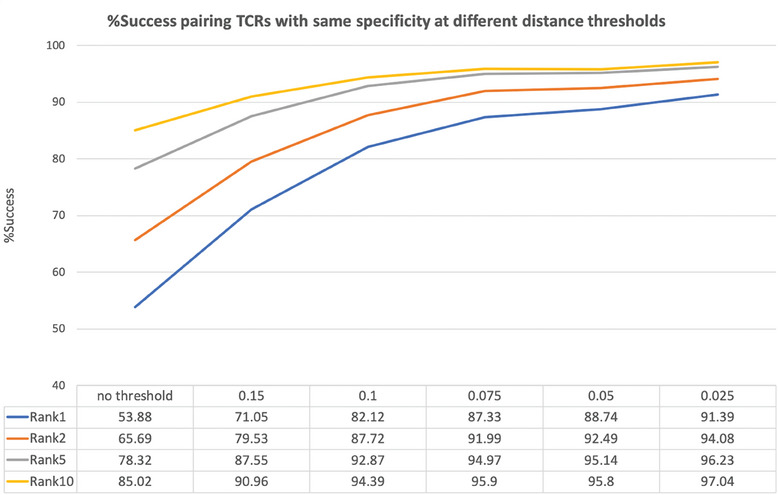
Percentage of success in pairing TCRs with the same specificity at different TCRpcDist‐3D distance thresholds using the 10X Genomics dataset, after removing the overrepresented KLGGALQAK peptide.

Encouraged by these results we further analyzed TCRpcDist‐3D distance behavior. We computed the probability of a TCR pair to share the same pMHC as a function of the TCRpcDist‐3D value (Figure S3, Supporting Information) using 10X Genomics data set. We observed that for this data set at a distance 0.15, the probability of a peptide pair to share the same pMHC was 50%. This probability increased when the distance between two TCRs decreased, reaching a maximum probability of 85% when the distance was 0. We recomputed the same probability as a function of the TCRpcDist‐3D value for the 10X subset without the overrepresented KLGGALQAK (Figure S3, Supporting Information) and the behavior was nearly the same. At a distance 0.17 the probability of a peptide pair to share the same pMHC was 50%. Again, the lower the distance between two TCRs the higher the probability they shared the same pMHC, reaching a maximum probability of ≈90% when the distance was 0. We studied how frequently, starting from a given TCR, it is possible to find another TCR with the same specificity in the 1, 2, 5, and 10 top‐ranked TCRs (**Figure** **5**). We performed this analysis without any distance threshold and afterward with thresholds of 0.15 and lower (Figure 5) considering that the probability of sharing the same specificity is higher at these values (inflexion point in the sigmoid curve, Figure S3, Supporting Information). TCR pairs with the closest TCRpcDist‐3D (rank 1) shared the same specificity in 53.9% of the cases, which is much better than what can be obtained by random assignments (19% success; p‐value < 0.0001). The predictive power of TCRpcDist‐3D was increased considering the top 5 and the top 10 closest TCRs. Indeed, the specificity of a given TCR was shared by at least one of the 5 and 10 top‐ranked TCRs according to TCRpcDist‐3D in 78.3% and 85.0% of the cases, respectively (p‐value < 0.0001). By applying distance thresholds, the predictive ability of TCRpcDist‐3D was significantly increased at the cost of decreasing the number of TCRs analyzed. For example, in 91.0% of the cases, a given TCR of the dataset shared its specificity with at least one of the other 10 top‐ranked TCRs, if only TCR pairs at a distance lower than 0.15 were considered (Figure 5). 55.5% of the TCRs from the repertoire were analyzed with this threshold. We concluded that TCRpcDist‐3D values lower than 0.15 provided a good compromise between accuracy in the specificity prediction and number of TCRs under study.

We further observed that TCRs less frequently found in the reference set (<0.7%, **Table** **3**) are rarely paired with the TCR of the same specificity, while TCRs with higher representations are easier to pair (Table 3). To tackle the problem of different abundance in the repertoire (which we decided to keep mimicking clinical data) we introduced the enrichment factor (EF), a metric which determines if the abundance of the TCRs with a given pMHC specificity has increased in the top 10 closest TCRs relative to the average abundance in the entire reference database. For a given specificity, EF is calculated as the ratio between the frequency of the TCRs with that specificity in the 10 TCRs closest to the query, and the frequency of the TCRs with that specificity in the entire reference repertoire. The analysis of the EF of the TCRs with same specificities allows to check if their presence in the top 10 is only an accidental result of their abundance in the repertoire, or an effect of their similarity to the input TCR, increasing the confidence in the prediction. We found that we can confidently predict that two TCRs share the same specificity when their distances are very low (≪0.15) and their EF is high within the study set.

**Table 3:** Frequency of the TCRs with the same specificity correctly paired in the rank1 and in the rank10 when no threshold is applied. Results obtained when applying TCRpcDist‐3D to the 10X Genomics data without the overrepresented peptide KLGGALQAK.

Frequency	Peptide	Relative frequency rank1	Relative frequency rank10
25.20	AVFDRKSDAK	34.08	93.10
0.72	AYAQKIFKI	0.00	0.00
0.10	CLLWSFQTSA	0.00	0.00
0.31	CYTWNQMNL	0.00	0.00
6.44	ELAGIGILTV	62.70	91.27
0.46	FLASKIGRLV	0.00	0.00
0.87	FLYALALLL	70.59	100.00
26.33	GILGFVFTL	84.66	96.12
2.25	GLCTLVAML	43.18	88.64
0.20	IMDQVPFSV	0.00	0.00
0.15	IPSINVHHY	0.00	0.00
6.85	IVTDFSVIK	32.84	64.93
0.46	KTWGQYWQV	0.00	0.00
0.15	KVLEYVIKV	0.00	0.00
0.31	LLDFVRFMGV	0.00	0.00
0.82	LLFGYPVYV	0.00	6.25
0.20	MLDLQPETT	0.00	0.00
0.15	QPRAPIRPI	0.00	0.00
21.17	RAKFKQLL	69.08	97.34
5.32	RLRAEAQVK	9.62	44.23
0.72	RTLNAWVKV	0.00	7.14
0.15	SLFNTVATL	0.00	0.00
0.36	SLFNTVATLY	0.00	0.00
0.15	YLLEMLWRL	0.00	0.00
0.15	YLNDHLEPWI	0.00	0.00

### 2.5 Successful TCRs Deorphanization of Tumor‐Infiltrating Lymphocytes (TILs) from Cancer Patients Using TCRpcDist‐3D

TCRpcDist‐3D and the clustering described herein were used for the prediction of TCR specificity, i.e., predicting which pMHC a TCR could bind. This was done by applying the similarity principle which states that similar molecules are likely to share similar bioactivities. Following a strategy well‐known in computer‐aided drug design to predict the targets of bioactive compounds,^[^ ^30^ ^]^ consisting in i) reverse‐screening a library of TCRs for which we know the specificity (a.k.a. the reference set) to identify those that are the most similar to the orphan TCR according to TCRpcDist‐3D, and ii) infer the orphan TCR's probable specificity from the top 1, 2, 5 to 10 most similar TCRs in the reference set and their distance from the orphan TCR. Of course, using this approach, it is only possible to predict a potential specificity for an orphan TCR if it belongs to the specificities of the reference TCRs with which it will be compared. Consequently, due to the limited number of TCRs for which the cognate pMHC is already known, it may be that none of the orphan TCR binds any of the candidate epitopes. Nevertheless, unlike machine learning approaches that require multiple TCRs with a given specificity to make predictions, TCRpcDist can make predictions with just a single TCR of known specificity as a reference. If an orphan TCR is found to have a small distance (≪0.15) from this single TCR with known specificity, we can be highly confident that the orphan TCR shares the same specificity.

We applied TCRpcDist‐3D to private data with known and unknown specificities from the previously mentioned 4 melanoma patients (private set Mel#1‐Mel#4), 2 additional melanoma, 1 gastrointestinal and 1 lung cancer patients. We carried out four tests to validate the capacity of TCRpcDist‐3D to deorphanize TCRs based on the similarity principle. In all cases, we were able to successfully predict their specificities and thus to deorphanize TCRs.

**Figure 6:**
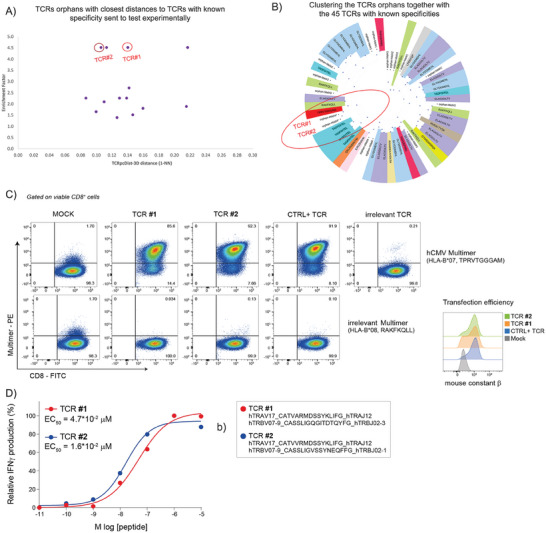
Using TCRpcDist‐3D to deorphanize TCRs found in cancer cells of patients Mel#1, Mel#2, Mel#3, Mel#4. A) TCRs orphans tested experimentally. Each dot corresponds to the TCRpcDist‐3D value between the orphan TCR and the closest reference TCR, and the corresponding EF value; B) the hierarchical clustering tree of the TCR orphans tested experimentally and the TCRs with known specificity used as reference; C) validation of peptide‐specificity predicted through TCRpcDist‐3D (round 1 of experiments). Validation of antigen‐specificity for two positive TCRs found by TCRpcDist‐3D screening. TCRalpha‐ and TCRbeta‐coding RNA was transfected into recipient Jurkat cells engineered for human CD8 expression and CRISPR TCRalphabeta‐KO. After over‐night incubation cells were stained with the CMV pp65‐multimer (TPRVTGGGAM, HLA‐B*07). A previously identified pp65‐specific TCR and an irrelevant TCR were used respectively as technical positive (CTRL+) and negative control. An irrelevant EBV B2LF‐1‐multimer (RAKFKQLL, HLA‐B*08) was used to further confirm the specificity of the two predicted TCRs; D) functional characterization of two TCRs predicted through TCRpcDist‐3D in the first round of experiments. The functional avidity of both CMV pp65‐specific TCRs was measured using activated primary T cells. Shown are the normalized relative frequencies of IFNγ‐producing T cells and the EC_50_ (effect concentration 50%, peptide concentration required for half‐maximal T cell activation) is given for each TCR. Color‐coded corresponding TCR sequences are reported on the right.

First, we have studied 8′224 intratumoral orphan TCRs from four melanoma patients, 1856 from Mel#1, 2792 from Mel#2, 2011 from Mel#3, and 1564 from Mel#4 (Table S2, Supporting Information) and screened them against the TCRs with known specificity within the same patient for the first three patients and within all patients for the fourth (Mel#4) (Table S2, Supporting Information). For patient Mel#4 we knew in advance that some orphan TCRs (annotated in Table S2, Supporting Information were reactive against a pool of 32 viral epitopes from Cytomegalovirus, Epstein‐Barr virus, and Influenza viruses (Table S6, Supporting Information). Consequently, we screened them against TCR specific to these peptides, even though they belonged to a different patient. Among the 8′224 orphans TCRs studied, only 11 had a TCRpcDist‐3D lower than 0.15 to a given TCR with known specificity, and none of them lower than 0.09 (**Figure** **6**A,B and **Table** **4**). We experimentally tested these 11 TCRs together with 5 additional orphan TCRs that presented TCRpcDist‐3D distances between 0.15 and 0.25 to a reference TCR, with consequently even lower probability of a correct specificity prediction (≪50%, Figure S3, Supporting Information). The predicted cognate pMHC of 2 TCRs out of these 16 orphan ones were experimentally validated (Figure 6C,D). They corresponded to those exhibiting the lowest distances together with the highest enrichment factor, i.e., a TCRpcDist‐3D of 0.11 and 0.14 to the closest TCRs with known specificity and an enrichment factor of 4.5 in both cases (Figure 6A). These two TCRs were specific to TPRVTGGGAM:HLA‐B*07 and, in the set of 45 reference TCRs, spontaneously grouped with the single TCR recognizing TPRVTGGGAM:HLA‐B*07 (Figure 6B). The sequence details of the orphan TCRs experimentally validated and their closest TCR with known specificity can be seen in Table 4.

**Table 4:** The orphan TCRs sent to test in the four rounds of experiments (distances < 0.15, probability of correct prediction > 50%) and the closest neighbor TCR with known specificities. The closest neighbors allowed us to infer the specificity of the orphans. The peptide, the TCRpcDist‐3D distance, the Enrichment Factor (if applied), and the figure correspondence are also present. The TCRs deorphanized and the closest neighbors are in bold.

Round	Patient	Alpha Vseq	Alpha Jseq	CDR3α	Beta Vseq	Beta Jseq	CDR3β	peptide specificity	TCRpsDist‐3D	EF	Figure correspondance
1	Mel#2	TRAV12‐2	TRAJ52	CAVTTGGTSYGKLTF	TRBV11‐2	TRBJ2‐7	CASKGAPTIYYEQYF	orphan	0.13	1.4	
	Mel#2	TRAV12‐2	TRAJ52	CAVKGSGTSYGKLTF	TRBV9	TRBJ2‐1	CASSLTGYEQFF	GLYDGMEHL			
	Mel#2	TRAV1‐2	TRAJ26	CAVRFRDNYGQNFVF	TRBV5‐1	TRBJ2‐1	CASSLSWTSGNEQFF	orphan	0.09	2.3	
	Mel#2	TRAV1‐2	TRAJ28	CAVRTGYSGAGSYQLTF	TRBV5‐1	TRBJ2‐1	CASSYGNEQFFG	TADFDITEL			
	Mel#4	TRAV17	TRAJ12	CATVVRMDSSYKLIF	TRBV7‐9	TRBJ2‐1	CASSLIGVSSYNEQFF	orphan	0.11	4.5	Figure 5 – TCR #2
	Mel#2	TRAV17	TRAJ12	CATVVRMDSSYKLIF	TRBV7‐9	TRBJ2‐1	CASSLVGEGWSDEQFF	TPRVTGGGAM			
	Mel#2	TRAV12‐2	TRAJ49	CAGSTGNQFYF	TRBV27	TRBJ2‐3	CASSPWGASDTQYF	orphan	0.10	1.6	
	Mel#2	TRAV12‐2	TRAJ49	CAVNAGNQFYF	TRBV4‐1	TRBJ2‐3	CASSPDRSADTQYF	ELAGIGILTV			
	Mel#2	TRAV12‐1	TRAJ42	CVVNSYGGSQGNLIF	TRBV9	TRBJ1‐2	CASSVVSGGTYGYTF	orphan	0.11	2.1	
	Mel#2	TRAV12‐2	TRAJ37	CAVKDGNTGKLIF	TRBV9	TRBJ1‐2	CASSLTGYGYTF	GLYDGMEHL			
	Mel#4	TRAV14/DV4	TRAJ4	CVSGGYNKLIF	TRBV12‐4	TRBJ1‐6	CASGSGNSPLHF	orphan	0.11	4.5	
	Mel#1	TRAV14/DV4	TRAJ4	CAMRAGGYNKLIF	TRBV14	TRBJ2‐5	CASSHWTSGSGETQYF	ILRGSVAHK			
	Mel#1	TRAV13‐1	TRAJ3	CAAGLGRYSSASKIIF	TRBV20‐1	TRBJ2‐5	CSAKRTSGHQETQYF	orphan	0.13	2.3	
	Mel#1	TRAV13‐1	TRAJ3	CAASDSSASKIIF	TRBV9	TRBJ2‐5	CASSVGKETQYF	FAFGEPREL			
	Mel#1	TRAV26‐2	TRAJ30	CILRDVGRDDKIIF	TRBV9	TRBJ2‐5	CASSARQGRGETQYF	orphan	0.14	2.3	
	Mel#1	TRAV13‐1	TRAJ3	CAASDSSASKIIF	TRBV9	TRBJ2‐5	CASSVGKETQYF	FAFGEPREL			
	Mel#1	TRAV35	TRAJ30	CAGQVVMDDKIIF	TRBV9	TRBJ2‐5	CASSPPVGETQYF	orphan	0.14	2.3	
	Mel#1	TRAV13‐1	TRAJ3	CAASDSSASKIIF	TRBV9	TRBJ2‐5	CASSVGKETQYF	FAFGEPREL			
	Mel#4	TRAV17	TRAJ12	CATVARMDSSYKLIF	TRBV7‐9	TRBJ2‐3	CASSLIGQGITDTQYF	orphan	0.14	4.5	Figure 5 – TCR #1
	Mel#2	TRAV17	TRAJ12	CATVVRMDSSYKLIF	TRBV7‐9	TRBJ2‐1	CASSLVGEGWSDEQFF	TPRVTGGGAM			
	Mel#2	TRAV13‐1	TRAJ4	CAVPGVLSGGYNKLIF	TRBV9	TRBJ1‐1	CASSVASPNTEAFF	orphan	0.15	1.5	
	Mel#2	TRAV13‐1	TRAJ37	CASYSGNTGKLIF	TRBV9	TRBJ2‐5	CASSVTSGTLYF	TADFDITEL			
2	Mel #5	TRAV12‐1	TRAJ12	CVVNGEDSSYKLIF	TRBV2	TRBJ2‐2	CASSEGQVAPGELFF	EBV – reactive	–	7.6	Figure S4 (Supporting Information) – TCR#1
	Mel #5	TRAV12‐1	TRAJ12	CVVNGMDSSYKLIF	TRBV2	TRBJ2‐2	CASSAGQVAPGELFF	EBV – reactive	0.00		Figure S4 (Supporting Information) – TCR#2
	10X genomics	TRAV12‐1	TRAJ12	CVVNGGDSSYKLIF	TRBV2	TRBJ2‐2	CASSEGQVSPGELFF	GLCTLVAML	0.06		
	GI #1	TRAV5	TRAJ31	CAEDNNARLMF	TRBV20‐1	TRBJ1‐2	CSARDRTGNGYTF	orphan	–	28.0	Figure S4 (Supporting Information) – TCR#3
	10X genomics	TRAV5	TRAJ31	CAEDNNARLMF	TRBV20‐1	TRBJ1‐2	CSARDSTGNGYTF	GLCTLVAML	0.02		
3	Lung #1	TRAV26‐2	TRAJ53	CILSDGGSNYKLTF	TRBV2	TRBJ2‐7	CASSEPGYEQYF	orphan	0.00	not applied	Figure S5A (Supporting Information) – TCR#3
	Lung #1	TRAV26‐2	TRAJ53	CILSDGGSNYKLTF	TRBV2	TRBJ2‐7	CASSDPGYEQYF	DSNDYHILR			
	Lung #1	TRAV26‐2	TRAJ53	CIPSDGGSNYKLTF	TRBV2	TRBJ2‐1	CASSVPGYEQFF	orphan	0.00		Figure S5A (Supporting Information) – TCR#2
	Lung #1	TRAV26‐2	TRAJ53	CILSDGGSNYKLTF	TRBV2	TRBJ2‐7	CASSDPGYEQYF	DSNDYHILR			
	Lung #1	TRAV3	TRAJ40	CAVRDISTTSGTYKYIF	TRBV28	TRBJ1‐2	CASSPPGDPIYGYTF	orphan	0.06		Figure S5A (Supporting Information) – TCR#1
	Lung #1	TRAV3	TRAJ40	CAVRDISTTSGTYKYIF	TRBV28	TRBJ1‐6	CASSSPGDSYNSPLHF	DSNDYHILR			
4	Mel #6	TRAV14/DV4	TRAJ26	CAMEEYGQNFVF	TRBV27	TRBJ2‐4	CASSLSGGLYNEQFF	orphan	0.02	not applied	Figure 6 + Figure S5B (Supporting Information) – TCR #1
	Mel #6	TRAV14/DV4	TRAJ26	CAIINYGQNFVF	TRBV27	TRBJ2‐4	CASSLSASGRVNIQYF	SLKLHYQL			
	Mel #6	TRAV12‐2	TRAJ52	CALGSAGGTSYGKLTF	TRBV6‐5	TRBJ1‐2	CASSPSGAPANYGYTF	orphan	0.05		Figure S5C (Supporting Information) – TCR #1
	10X genomics	TRAV12‐2	TRAJ52	CAVNLGLTAGGTSYGKLTF	TRBV6‐4	TRBJ1‐2	CASRAGTEISGYGYTF	ELAGIGILTV			
	Mel #6	TRAV12‐2	TRAJ49	CAVNTGNQFYF	TRBV6‐1	TRBJ1‐5	CASSEAGVGQPQHF	orphan	0.06		Figure S5C (Supporting Information) – TCR #2
	10X genomics	TRAV12‐2	TRAJ49	CAVPGSTGNQFYF	TRBV6‐3	TRBJ1‐5	CASSFGFGQPQHF	ELAGIGILTV			
	Mel #6	TRAV12‐2	TRAJ47	CAVTLTKYGNKLVF	TRBV11‐2	TRBJ1‐2	CASSLGGGPIGYTF	orphan	0.06		Figure S5C (Supporting Information) – TCR#3
	10X genomics	TRAV12‐2	TRAJ39	CAANAGNMLTF	TRBV11‐2	TRBJ1‐2	CASSLGGGTEAFF	ELAGIGILTV			
	Mel #6	TRAV12‐2	TRAJ45	CAVNPGGGADGLTF	TRBV28	TRBJ2‐1	CASTPPGTSGKSSYNEQFF	orphan	0.07		Figure S5C (Supporting Information) – TCR#4
	10X genomics	TRAV12‐2	TRAJ32	CAVNGGGATNKLIF	TRBV28	TRBJ1‐1	CAIPGPSNTEAFF	ELAGIGILTV			

Second, we selected 44 orphan TCRs, annotated as non‐tumor reactive from 2 additional patients (1 melanoma patient, Mel #5, and 1 patient with oesophagus gastrointestinal cancer GI #1) (Table S7, Supporting Information) and used TCRpcDist‐3D to deorphanize them against viral peptides present on 10X Genomics database. Two TCRs from patient Mel #5 and 1 TCR from patient GI #1 were predicted to bind the EBV BMLF1 GLCTLVAML peptide presented by HLA‐A*02:01, based on the closest distance to EBV BMLF1‐specific TCRs and were supported by high enrichment factors (Table 4). Of interest, these predictions were all successfully validated experimentally (Figure S4, Supporting Information).

Third, 1′054 orphan TCRs found in a lung cancer patient (Lung #1, Table S8, Supporting Information) were screened against 7 TCRs with known specificity to DSNDYHILR/HLA‐A*68:01 (Table S8, Supporting Information). Three orphan TCRs showed the closest distance to TCRs with known specificity. For all of them the TCRpcDist‐3D distances between the orphan and the closest pair were lower than 0.06 (Table 4), corresponding to a probability of a correct prediction higher than 80% (Figure S3, Supporting Information). These specificity predictions were experimentally and successfully validated for those TCRs (Figure S5A, Supporting Information). Again, our approach proved to be able to identify, among orphan TCRs, candidates that are likely to be deorphanized and predict their specificities even though a very restricted number of TCRs with known specificities was used as reference.

**Figure 7:**
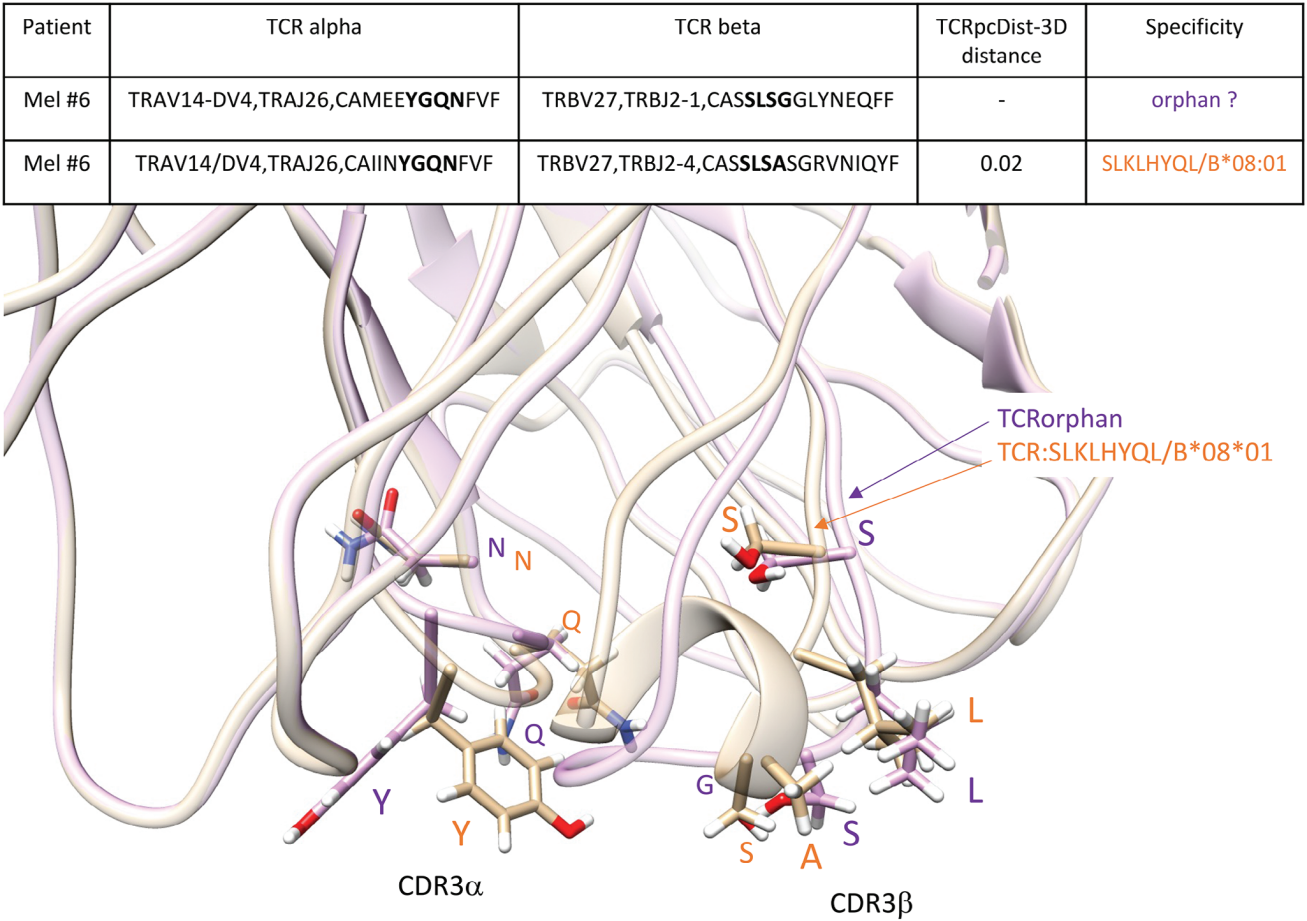
3D models superimposition of Mel#6 TCR specific for the neoAg SLKLHYQL/HLA‐B*08:01, in light brown with the orphan Mel#6 TCR with closest TCRpcDist‐3D distance, in light purple. Orphans and closest TCR are described in detail in Table 4. These TCRs exhibit a very small TCRpcDist‐3D distance, 0.02, since they share the same TRAV and TRBV and exhibit the same 4‐mer in CDR3α, YGQN, and nearly the same 4‐mer in CDR3β SLSA versus SLSG. The CDR3s 4‐mer features, highlighted in bold in the table and in sticks in the 3D models, are solvent exposed and able to interact with the peptide.

Finally, 2′111 orphan TCRs of a melanoma patient (Mel #6) were screened against TCRs from the same patient binding the neoantigen (neoAg) SLKLHYQL and the tumor associated antigen (TAA) ELAGIGILTV (Table S9, Supporting Information). These orphans were also screened against 10X Genomics TCRs that bind the TAAs ELAGIGILTV. One TCR was predicted to bind the neoAg SLKLHYQL/HLA‐B*08:01, which was experimentally validated (Table 4; and Figure S5B, Supporting Information). The orphan TCR and the closest TCR binding this neoAg SLKLHYQL/HLA‐B*08:01 that allowed this prediction, showed a TCRpcDist‐3D value of 0.02 (Table 4). This small distance is consistent with the fact that these TCRs share the same TRAV and TRBV and exhibit the same 4‐mer in CDR3α, YGQN, and nearly the same 4‐mer in CDR3β SLSA versus SLSG. These residues are solvent exposed and able to interact with the peptide, as evident from the structural models of these TCRs (**Figure** **7**). Due to the dissimilarities in terms of length and sequence of the CDR3s, especially CDR3β, this prediction could not have been done using full sequence based approaches like TCRdist3. As an illustration, in this case, TCRdist3 gives a normalized distance of 0.19 between the orphan TCR and the TCR with known specificity, which would not have led to selecting this TCR for deorphanization against this epitope. On the contrary, our approach gives a much smaller distance of 0.02, which triggered the above‐mentioned experimental validation. Further details can be seen in Table S11 (Supporting Information). Evidence of the response to the neo‐peptide SLKLHYQL by CD8+ TILs was recently published by Müller, M. et al.^[^ ^31^ ^]^ Four orphan TCRs were predicted to bind the TAA ELAGIGILTV/HLA‐A*02:01 peptide based on their closest distance to TCRs with the same specificity (Table 4). Two out of 4 predicted TCRs were shown to mediate T cell activation in vitro, thus experimentally validated for the predicted specificity (Table 4; and Figure S5C, Supporting Information).

## 3 Discussion

TCRpcDist‐3D is a state‐of‐the‐art approach that competes with existing approaches as shown by comparison with TCRdist3, TCRbase‐1.0, NetTCR‐2.2 and SwarmTCR. Our tool presents important methodologic refinements that result in performance improvements for some pMHC. Similar to the method of Ostmeyer et al., TCRpcDist uses the Atchley factors rather than the sequences to calculate distances between TCRs. However, unlike that approach, but similarly to TCRdist, it takes CDR loops 1, 2, and 3 into account, and not just CDR3β. TCRpcDist differs however from TCRdist by the fact that the latter considers also CDR loop 2.5 and performs a global sequence alignment prior to scoring the latter using BLOSUM62 derived parameters, while TCRpcDist‐3D tries to identify and uses the most relevant 4‐mers (i.e., four consecutive residues) to account for the fact that not all residues are likely to bind directly to pMHC. To accentuate the focus on the residues most likely to interact with the pMHC, TCRpcDist‐3D limits the search for 4‐mers containing only sufficiently solvent exposed residues according to TCR structural models. TCRpcDist‐3D is therefore a structural and biophysical approach that allows calculating distances between TCRs based on the physicochemical properties of the solvent exposed 4‐mers that are possible to construct from CDR1α, CDR2α, CDR3α, CDR1β, CDR2β, and CDR3β sequences. Such distances can subsequently be used to cluster TCRs or apply the similarity principle to try to predict TCR specificities. Moreover, TCRpcDist is broadly applicable and can be generalized without the need for supervised training.

Our approach is universal and can be applied to any specificity. Although we used a set of 54 TCRs with only 9 alleles and 16 specificities to develop the approach, it can predict binding for any epitope outside the training data. This capability was demonstrated by the successful application of our method to much larger and diverse validation sets. Moreover, we compared the predictive power of TCRpcDist‐3D with the gold standard approach TCRdist3 and observed that our model is better predicting specificities for some peptides including AVFDRKSDAK, NLVPMVATV, IVTDFSVIK, SPRWYFYYL, CINGVCWTV, RLRAEAQVK, and NQKLIANQF.

We applied TCRpcDist‐3D to private data with known and unknown specificities from 8 patients with different cancer types. We carried out experimental tests to validate the capacity of TCRpcDist‐3D of TCR deorphanization and we were able to select deorphanizable orphans and successfully predict their specificities, including viral, TAA and neoAg epitopes. Deorphanization was done based on the similarity principle, which posits that similar TCRs (distances ≪0.15) are likely to bind the same pMHC. The strategy involved: i) screening a library of TCRs with known specificities to identify the closest orphan TCRs according to TCRpcDist‐3D, and ii) inferring the orphan TCR's probable specificity based on the distances between them and the TCRs with known specificities in the reference library. First, we have challenged our approach and predicted specificities for two orphans TCRs from a pool of 8′224 TCRs found within 4 melanoma patients (Mel #1 to Mel #4). Among the 11 predictions for which the similarity principle could be applied with some confidence (distance < 0.15) we validated the specificity for two orphans for the same viral peptide, the ones with higher EF. Second, two TCRs from patient Mel #5 and 1 TCR from patient GI #1 were predicted to bind the EBV BMLF1 GLCTLVAML peptide presented by HLA‐A*02:01, based on the closest distance to EBV BMLF1‐specific TCRs of the private and 10X Genomics databases. Their TCRpcDist‐3D distances were very low, <0.06 with a high EF and these predictions were validated experimentally. Third, in patient Lung #1, three additional orphan TCRs showing the closest distance to TCRs specific for the neoAg DSNDYHILR/HLA‐A*68:01 (TCRpcDist‐3D distance < 0.06) were predicted to bind the same neoAg, which was experimentally validated. Finally, in patient Mel #6, 1 TCR was tested and found specific for the neoAg SLKLHYQL/HLA‐B*08:01 (TCRpcDist‐3D distance between orphan and TCR with known specificity <0.02). Four TCRs derived from patient Mel #6 were predicted and screened toward the TAA ELAGIGILTV/HLA‐A*02:01 peptide based on their closest distance to TCRs previously validated for the same specificity. Two out of 4 TCRs confirmed the predicted specificity to TAA. TCRpcDist‐3D can thus decipher immune recognition, which is critical for understanding a wide range of diseases and for the development of effective vaccines and immunotherapies. We were very successful screening orphans TCRs against TCRs with known specificity within the same patient, with more than 80% of the in silico predictions being validated. Screening orphan TCRs within the same patient ensured that the considered possible epitopes are displayed by the tumor of a given patient. As far as we know, TCRpcDist‐3D is the only in silico tool for TCR‐similarities estimation that demonstrated its ability to deorphanize TCRs. Applying TCRpcDist‐3D to additional private data with known and unknow specificities, from more patients and other cancer types, is ongoing.

TCRpcDist‐3D and the clustering described herein can be used to analyze the diversity of TCR repertoires, by grouping together those likely to bind the same pMHC. In addition, it can also be used for the prediction of TCR specificity, i.e., predicting which pMHC a TCR could bind. One possible approach is to cluster TCRs for which the cognate pMHC is known, together with orphans TCRs for which the target is unknown. The positioning of these orphan TCRs indeed provides an indication about possible cognate pMHC, i.e., those belonging to a group of TCRs that recognize one given peptide or those that have the closest distance to a TCR with a given specificity. TCRpcDist is able to provide new insights into T cell responses captured in the TCR repertoire and will facilitate the development of new clinical strategies to treat and monitor not only cancer but other infectious diseases.

TCRpcDist‐3D, as described here, has already been integrated with a Tumor Reactivity Predictor, showcasing the potential of this combination of algorithms to identify clinically relevant TCRs for personalized T cell therapy.^[^ ^32^ ^]^

### 3.1 Limitations of the Study

Our approach demonstrates high clustering efficiency when compared with the competitors but there is still room for further improvements. The weighting parameters and the solvent accessibility thresholds will be reassessed as soon as a significantly larger number of TCRs with known pMHC and 3D structures are available. The fact that the TCRpcDist calculates TCR distances based on 4‐mer features is a limitation, particularly when working with TCRs with long CDR loops, where more than 4 residues can be involved in the binding of the antigen. Using sequence features longer than 4‐mer in the approach may improve the clustering efficiency for longer CDR loops but will not be applicable for short ones. We are exploring the feasibility of including features of variable lengths. Another limitation of TCRpcDist is the normalization of the TCR pair distances by the largest calculated distance within a dataset. This means that normalization is performed within the dataset, requiring caution when comparing distances across different datasets. Our observations indicated that using a dataset‐dependent normalization factor better differentiated TCRs within that set by amplifying their distances and more effectively highlighting their differences, especially when TCRs with similar sequences were present within a dataset (data not shown). We may consider implementing a universal normalization in the next version of our approach. One more constraint of TCRpcDist is the fact that it uses one single conformation per TCR model, i.e., the lowest energy one among 10 conformations, to calculate the residues that may interact with the pMHC. The dynamic behavior of the CDR loops and the stochastic nature of the modelling approach may result in different residues available to recognize the pMHC within the same TCR sequence if we model it in different times. To circumvent this issue, we could sample more conformations to find the low‐energy one and/or make use of a conformational ensemble in order to determine the residues able to interact with a pMHC. Of note, although more sampling and an approach that makes use of a conformational ensemble would be feasible, it would also be substantially more time consuming which could be a limitation for large scale applications, for example on thousands of orphans’ TCRs from clinical sets. Last, but not least, our 3D modelling approach is based on the TCRmodel available in Rosetta since 2019, which among several other approaches evaluated at that time, offered an optimal balance between computational time and accuracy. In recent years new tools for TCR and TCRpMHC modelling have emerged, leveraging cutting‐edge machine learning and structural biology techniques. Noteworthy examples include AlphaFold,^[^ ^22^ ^]^ TCRmodel2,^[^ ^33^ ^]^ and ImmuneBuilder^[^ ^34^ ^]^ and we may consider including one of these in a near future.

Regarding the specificity predictions, TCRpcDist‐3D proved to be able to fish and deorphanize TCRs in cancer patients under clinical investigation. Additional specificity predictions in these and other patients will further be essential to describe the impact of our approach in cancer immunotherapy. Further studies are ongoing thanks to a larger private data set with more patients and more TCRs with known specificities.

To further improve our clustering efficiency and deorphanization capability we are also working on i) combining TCRpcDist with other methods following a consensus approach, so that we can combine their individual strengths and mitigate their limitations, and on ii) applying to additional databases.

## 4 Experimental Section

### TCRs Datasets – Developmental Set

The development set consisted in of 54 CD8+ TCR structures with known pMHC (Table S1, Supporting Information) taken from a set of 151 TCR‐pMHC complexes whose experimental structures were retrieved from the Protein Data Bank (PDB) in June 2020.^[^ ^25^ ^]^ TCR duplicates (having the same CDR 1, 2, 3; α and β sequence, independently of the pMHC they recognize) and singleton pMHC (pMHC recognized by only one single TCR, thus preventing the possibility of cognate TCR comparison) were removed from this set. Human and mouse structures were considered to increase the sampling size, with a total of 48 and 6 human and mouse TCRs, respectively. Singleton TCRs and redundant TCRs were removed, even though they could increase the sample size, as they did not contribute to the development of the model. Indeed: i) TCR singletons, cannot be paired, by definition, and this independently of the parameters used in TCRpcDist and ii) Pairs of redundant TCRs would always have zero or near zero distances, independently of the parameters. Since this approach considers aspects of TCR‐pMHC 3D interactions, The approach was developed using a developmental set of TCRs for which the TCR‐pMHC 3D structures were known. Due to this, a set of 54 TCRs was the largest set available for developing this approach as of June 2020. This set included structures with single point mutations (SP). As the SP mutations are in CDRs1, 2, and CDR3, they were keptfor the following reasons: 1) to increase the size of the set; 2) to check the effect of SP on the predictive ability, as they were placed in regions that contact the pMHC and 3) it was known that SP mutations may result in different TCR specificity. By excluding TCRs with SP mutations from the 54‐set it was ended up with a training set of 48 TCRs. The PDB identifiers excluded from the 54 set in Table S1 (Supporting Information) were 2P5W, 2PYE, 2VLR, 3MV8, 3MV9, and 5NQK. Benchmarking the approach using this developmental set of 48 structures the same conclusions were reached regarding the best combination of parameters to use in this approach (Data S5, Supporting Information). In July 2024, This approach was reevaluated using a collection of 96 TCRs that recognize 48 distinct known pMHCs retrieved from PDB (Data S2, Supporting Information).

### TCRs Datasets – Assessment Sets

The first assessment set consisted in a private collection of 45 CD8+ TCR sequences with known pMHC (Table S2, Supporting Information) found in 4 melanoma cancer patients (Mel #1, Mel#2, Mel#3, and Mel#4). These TCRs did not overlap with the TCRs used in the developmental set. They constitute a new test set that could be used by the research community working in this field. Further details about the patient data and TCR determination for these cancer patients were given below across this Experimental Section.

For purposes of comparison with competing approaches a subset of 84 non‐redundant and non‐singleton Human CD8+ TCRs with known specificity taken from PDB in January 2023 was worked with. The initial developmental set of 54 TCRs could not be compared from PDB with the TCRdist3 approach as it contains mouse and human TCRs together and both species could not input in the tcrdist3 algorithm.

Further assessment of this approach was done on a much larger data set that consists in CD8+ TCR sequences taken from 10X Genomics data (Single Cell Immune Profiling Dataset by Cell Ranger, 10x Genomics, version 3.0.2, 4 patients). Only true cells (with a “True” label in the “is_cell” column of the all_contig_annotations.csv file) and cells expressing high confidence were kept for further analyses. TCR pairs lacking some important information like one of the chains, one gene, the CDR3 information, and the cognate pMHC were also removed. Moreover, TCRs that express one single α and one single β chains were worked with and removed redundant, singleton, and cross‐reactive TCRs. This generated a list of 8′528 CD8+ TCRs (Table S4, Supporting Information) that could be modeled in 3D (see below) and of which 77.1% bind the CMV peptide KLGGALQAK bound to the A*03:01. Detailed analysis was performed without the KLGGALQAK viral peptide that represents 77.1% of the database (Table S10, Supporting Information for the pMHC representation in the 10X) and therefore could bias the analysis performed. After removing the TCRs binding the KLGGALQAK peptide, it was ended up with 1′956 TCRs.

A last assessment of this approach was done on VDJdb, which includes, among others, 10X Genomics data, PDB data, and several COVID‐19 related TCRs.^[^ ^35^ ^]^ The data was retrieved on 22.08.2022 and filtered for “HomoSapiens” TCRs. TCR pairs lacking some important information like one of the chains, one gene, the CDR3 information, and the cognate pMHC were also removed. Moreover, TCRs that express one single α and one single β chains were worked with and removed redundant TCRs. TCRs single point mutations were considered for better mimicking real case applications. This generated a list of 23′814 CD8+ TCRs of which 18′708 could be modeled in 3D (see below). Among the TCRs modeled, 55.6% bind the CMV peptide KLGGALQAK presented by HLA*03:01. After removing this overrepresented peptide, it was ended up with 8′128 TCRs, recognizing 671 peptides of which 337 were singleton and 334 were bound by at least 2 TCRs. The 8′128 TCRs set (Table S5, Supporting Information) was used for benchmarking. The set was labeled as VDJdb2022.

### TCRs Datasets – Deorphanizations

Four datasets were used to validate the capacity of TCRpcDist‐3D for deorphanization and were able to fish deorphanizable TCRs in all of them and successfully predict their specificities.

First, 8′224 orphan TCRs (Table S2, Supporting Information) determined by single‐cell experiments and found in 4 cancer patients (Mel #1, Mel#2, Mel#3, and Mel#4) were used with the objective of deorphanizing some of them. They were screened against TCRs with known specificity found within the same 4 melanoma patients (Table S2, Supporting Information).

Second, 44 orphan TCRs were selected from 2 additional patients (1 melanoma patient, Mel #5, and 1 patient with oesophagus gastrointestinal cancer GI #1) determined by single‐cell experiments and identified as non‐tumor‐reactive (Table S7, Supporting Information) and they were deorphanized them against viral peptides present on 10X Genomics.

Third, 1′054 orphan TCRs determined by single‐cell experiments and found in a lung cancer patient (Lung #1) were screened against the 7 TCRs with known specificity to DSNDYHILR/HLA‐A*68:01 found within the same patient, see Table S8 (Supporting Information).

Fourth, 2′111 orphan TCRs found in a melanoma patient (Mel #6) were screened against TCRs with specificities to neo and TAA found within the same patient, see Table S9 (Supporting Information).

Further details about the patient data and TCR determination for these cancer patients were given below.

### Modeling TCR 3D‐Structures from Sequence

The Rosetta “TCRmodel” protocol^[^ ^20^ ^]^ was applied to find the best TCR templates and model the 3D structures of the TCRs from the sequences (PDB, 10X Genomics, VDJdb2022, and private sets). RosettaTCR was designed to use all known TCR structures as templates and were optimized to correctly model TCR architectures. If no template was identified among existing TCR structures, CDR loops were obtained from antibody crystal structures, or were modeled de novo. A total of 10 models were produced for each TCR, and the highest‐ranked one according to the Rosetta energy function^[^ ^20^ ^]^ was selected as the final model for the TCR 3D‐structure. The modeling of the TCR was the limiting step of this approach and takes ≈1 min on a 16‐CPU and was therefore feasible for large‐scale applications. The final number of TCRs sequences for each one of the described datasets correspond to TCRs for which 3D models were obtained. Indeed, 19.6% of TCR sequences from 10X Genomics could not be modeled due to the lack of relevant templates (which means that the regular expressions used by the software to identify CDR1, 2, and 3 for one of the chains provided no satisfying result).

### Solvent Accessibility Calculation

The solvent accessibility of each CDR1α, CDR2α, CDR3α and CDR1β, CDR2β, and CDR3β residue was determined as the relative solvent excluded surface area (SESA) computed with the MSMS package of the UCSF Chimera software,^[^ ^36^ ^]^ as described elsewhere.^[^ ^37^ ^]^ The normalized SESA, nSESA, was calculated by normalizing the surface area of the residue in the TCR of interest by its surface area in a reference state. The latter was defined as the Gly‐X‐Gly tripeptides in which X was the residue type of interest.^[^ ^38^ ^]^ nSESA thus ranges from 0% for totally buried residues to 100% for residues exposed to the solvent to the same degree as in Gly‐X‐Gly.

### TCRs Distance Calculation and Clustering

The clustering pipeline is summarized in Figure 1 and consists in the following main steps:

First, all possible sliding windows of 4 residues that constitute the so‐called 4‐mer subunits were identified. The first four and last 3 residues from the CDR3 were excluded from the process. Alternatively, CDR residues with low SESA in the structural models might be excluded from this process since these residues were unlikely to contact the pMHC. Indeed, after a benchmark of the TCR clustering of the PDB set (details in the results section), using only the residues of CDR1s and CDR2s (α and β) with SESA > 5% and CDR3s (α and β) with SESA > 20% was found to improve the quality of the clustering approach. These weights were confirmed as the best in an external test set of 374 TCR unique models extracted from VDJ in 2019 (see Section S4, Supporting Information). Solvent accessibility per residue per TCR for the PDB set can be read in Supporting Information (Section S2, Supporting Information).

Second, each 4‐mer subunit was converted into a biophysicochemical representation using 5 Atchley factors that describe i) hydrophobicity, ii) secondary structure, iii) size/mass, iv) codon degeneracy, and v) electric charge.^[^ ^18^ ^]^ Given the encoding of residues using Atchley factors, the distance between two sets of 4 consecutive residues was calculated as the Manhattan distance between the two corresponding matrices M1 and M2, i.e., d(M1, M2) where:

(1) $$\begin{array}{c} d\left(M1,M2\right)=\sum_{i=\left[1:4\right]}\left|H_{1,i}-H_{2,i}\right|+\left|SS_{1,i}-SS_{2,i}\right|+\left|SM_{1,i}-SM_{2,i}\right|\\ +\left|CD_{1,i}-CD_{2,i}\right|+\left|ES_{1,i}-ES_{2,i}\right| \end{array}$$

Here, H_1,i_ is the Hydrophobicity Atchley factor of residue *i* in Matrix 1, H_2,i_ is the Hydrophobicity Atchley factor of residue *i* in Matrix 2, SS_1,i_ is the Secondary Structure Propensity Atchley factor of residue *i* in Matrix 1, etc.

Third, to calculate the distance between two corresponding loops, such as the CDR3β of two different TCRs, the matrices of Atchley factors for the *n* possible 4‐residues sliding windows of the first TCR and the *m* 4‐residues sliding windows of the second TCR were generated. Then, the distance between each possible corresponding pair of matrices was comprehensively calculated. The smallest distance, d_min_, between all possible pairs of matrices was retained as the distance between the two CDR3β loops.

(2) $$d\left(TCR1\;CDR3\beta ,\;TCR2\;CDR3\beta \right)=min\left(d{\left(M_{i},M_{j}\right)}_{i\in \left[1,n\right],\;j\in \left[1,m\right]}\right)$$

The above equation to calculate the distance between two CDR3β of two different TCRs was also applied to all CDR1s and CDR2s. Finally, the distance between the two TCRs was defined as the (possibly weighted) sum of the calculated distances between each pair of CDR1α, CDR2α, CDR3α, CDR1β, CDR2β, and CDR3β loops.

Of note, when analyzing a set of multiple TCRs, all distances between each possible pair of TCRs were calculated as described above. These distances were then normalized by dividing each of them by the largest calculated distance for all possible pairs. Consequently, the final distance between two TCRs ranges from 0 (for two TCRs bearing an identical set of 4 residues on each considered loop – only CDR3β or all CDRs) to 1 (for the maximum dissimilarity between two CDRs within a data set).

Finally, the calculated distances between TCRs within a set could be used to construct a hierarchical clustering tree (Figure 1). After an optimization of the TCR clustering approach using the PDB set (details in the results section), it was found that the best clustering was obtained when giving a weight of 10% to the contributions of CDR1s and CDR2s (α and β) and of 30% to those of CDR3s (α and β) in the calculation of the distance. These weights were confirmed as the best in an external test set of 374 TCR unique models extracted from VDJ in 2019 (see Data S6, Supporting Information). The generic hierarchical clustering algorithm UPGMA (unweighted pair group method with arithmetic mean) was used. The ETE3 python toolkit^[^ ^39^ ^]^ was employed for the visualization of the hierarchical clustering trees. Each TCR in the tree was colored according to its specificity (pMHC). As a qualitative measure of the efficacy of the clustering, the number of times the color changed between two successive nodes was determined, starting from the upper node of the hierarchical clustering and turning clockwise. The quality of the clustering was also determined by a more quantitative metric though non‐standard, pMHC‐distance, using the python library for phylogenetic computing, DendroPy,^[^ ^40^ ^]^ version 4.5.2, and the class PhylogeneticDistanceMatrix. pMHC‐distance was defined as the average branch length distance between all possible pairs of TCR nodes that recognize the same pMHC. The p‐values were calculated by randomization tests when discussing pMHC‐distances and color changes. For this, the actual TCR distances were randomly permuted before performing again the hierarchization and recalculating the corresponding pMHC‐distances and color changes. The process was repeated thousands of times to estimate the p‐value in a test with the appropriate precision. On top of these metrics, to test the predictive power of TCRpcDist, more “standard metrics” were used. A TCR classifier was defined that assigns a given TCR to the TCRs within the repertoire with the lowest distances (nearest neighbor or NN‐distance). In other words, for a given TCR, the nearest neighbor was the TCR with the smallest TCRpcDist distance among all the TCRs within the dataset, excluding itself. The sensitivity and specificity of the classifier were measured. To test the predictive power for a given pMHC, 6 cross‐validations were done by randomly allocating 30% of all TCRs specific for this pMHC as a reference. It was tested how often the TCRs with the lowest distances recognize the same peptide when compared with the reference, by measuring sensitivity and specificity. For each given pMHC, the average of the area under these receiver operating characteristic curves (AUC), a standard metric of the classification success, was calculated together with its standard deviation. Statistically significant differences between distributions were determined by two‐tailed paired students t‐tests.

### Patients and Regulatory Issues

Tumor samples used in this study (scTCR‐sequencing) were collected from six patients with metastatic melanoma and lung cancers. TCRs from two additional patients with melanoma and gastrointestinal cancers were also used. These eight patients were enrolled in phase I clinical trial approved by the institutional regulatory committee at Lausanne University Hospital (Ethics Committee, University Hospital of Lausanne‐CHUV): Mel #1‐#5 (trial NCT03475134, https://www.biorxiv.org/content/10.1101/2022.12.23.519261v1) and Mel #6, Lung #1 and GI #1 (trial NCT04643574). Patients’ recruitment, and study procedures were approved by regulatory authorities and all patients signed written informed consents. Data included in this study was comprised of both published and unpublished data.

### Tissue Processing

Resected baseline tumors (prior TIL‐ACT, tumors used to generate TIL products) were chopped into 1–2 mm2 pieces and cryopreserved in 90% human serum + 10% dimethyl sulfoxide (DMSO). For single‐cell experiments, both frozen and fresh material were used as starting material. The day of the assay, pathofrozen pieces were thawed in RPMI + 10% FBS and chopped in small pieces using a scalpel. Tissue was dissociated in RPMI + 2% Gelatin (#G7041, Sigma‐Aldrich) + 200 IU mL^−1^ Collagenase I (#17100‐017, ThermoFisher Scientific) + 400 IU mL^−1^ Collagenase IV (#17104‐019, ThermoFisher Scientific) + 5 IU mL^−1^ Deoxyribonuclease I (#D4527, Sigma‐Aldrich) + 0.1% RNasin Plus RNase Inhibitor (#N2618, Promega) for 15–30 min (depending of sample size and consistency) at 37 °C and shaken at 160 rpm. Digested cells were filtered using a 70 µm strainer and resuspended in PBS + 1% Gelatin + 0.1% RNasin. Cells were manually counted with hematocymeter then stained for viability with 50 uM mL^−1^ of Calcein AM (#C3099, Thermo Fisher Scientific) and FcR blocked (#130‐059‐901, Miltenyi Biotec) for 15 min at RT. After incubation and washing, cells were stained with CD45‐APC (#304 012, BioLegend) for 20 min at 4 °C. After washing, cells were resuspended in PBS + 0.04% BSA (Sigma‐Aldrich) + 0.1% RNasin and DAPI staining (Invitrogen) was performed

### Single‐Cell RNA and TCR Sequencing

CD45 live cells were sorted with the MoFlo AstriosEQ (Beckman Coulter) and manually counted to assess viability with Trypan blue. Ex vivo CD45 cells from tumors were resuspended at a density of 600–1200 cells µL^‐1^ with a viability of >90% and subjected to a 10X Chromium instrument for single‐cell analysis. The standard protocol of 10X Genomics was followed and the reagents for the Chromium Single Cell 5′ Library and V(D)J library (v1.0 Chemistry) were used. 12′200 cells were loaded per sample, with the targeted cell recovery of 7′000 cells according to the protocol. Using a microfluidic technology, single cell were captured and lysed, mRNA was reverse transcribed to barcoded cDNA using the provided reagents (10X Genomics). 14 PCR cycles were used to amplify cDNA and the final material was divided into two fractions: first fraction was target‐enriched for TCRs and V(D)J library was obtained according to manufacturer protocol (10X Genomics). Barcoded V(D)J libraries were pooled and sequenced by an Illumina HiSeq 2500 Sequencer. The second fraction was processed for 5′ gene expression library following the manufacturer's instruction (10X Genomics). Barcoded samples were pooled and sequenced by an Illumina HiSeq 4000 sequencer.

The scRNA‐seq reads were aligned to the GRCh38 reference genome and quantified using *Cellranger* count (10x Genomics, version 3.0.2). scTCR‐seq (VDJ) data were aligned to the same human genome using the cellranger vdj (10X Genomics, version 3.1.0). Only true cells (with a “True” label in the “is_cell” column of the all_contig_annotations.csv file) were kept for further analyses. Cells from the VDJ sequencing were mapped to the scRNA‐Seq data (5'GEX). This allowed to only select CD8^+^ TCR clones for downstream analyses.

### Peptide Synthesis

Peptides produced by the Peptides and Tetramers Core Facility (PTCF) of the University of Lausanne were HPLC purified (≥90% pure), verified by mass spectrometry, and kept lyophilized at −80 °C.

### TCR Validation

To validate antigen specificity and interrogate T cell reactivity, TCRα/β pairs were cloned into recipient Jurkat cell line (TCR/CD3 Jurkat‐luc cells (NFAT), Promega, in‐house stably transduced with human CD8α/β and TCRα/β CRISPR‐KO) and activated peripheral T cells., as previously described^[^ ^29^, ^41^ ^]^ with minor modifications. In brief, paired α and β chains were annotated based on TCRpcDist‐3D, and corresponding full‐length codon‐optimized DNA sequences were synthesized at GeneArt (Thermo Fisher Scientific) or Telesis Bio. DNA strings served as template for in vitro transcription (IVT) and polyadenylation of RNA molecules as per the manufacturer's instructions ((HIScribe T7 ARCA mRNA kit, NEB), followed by co‐transfection into recipient T cells.

Jurkat cells were electroporated using the Neon electroporation system (Thermo Fisher Scientific) with the following parameters: 1325 V, 10 ms, 3 pulses. Electroporated Jurat cells were either co‐cultured with peptide‐pulsed HLA‐matched presenting cells followed by a bioluminescence assay or interrogated by pMHC‐multimer staining to assess peptide‐specificity. For the luciferase assay, 2 × 10^4^ Jurkat cells were cocultured with 4 × 10^4^ PHA‐activated CD4 T cells (CD4 blasts) in the presence of 1 mm specific peptides in 96‐well plates. After overnight incubation, the assay was performed using the Bio‐Glo Luciferase Assay System (Promega). The following experimental controls of TCR transfection were included: mock (transfection with water) and a control TCR (irrelevant crossmatch of a TCRα and β chain from a private TCR library). Luminescence was measured with a Spark Multimode Microplate Reader (Tecan). As positive control of T cell aspecific stimulation Jurkat cells were cultured in the presence of TransAct (Miltenyi). Alternatively, Jurkat cells were interrogated by pMHC‐multimer staining with the following surface panel: anti‐CD3 APC Fire 50 (SK7, Biolegend), anti‐CD8 Pacific Blue (RPA‐T8, BD Biosciences), anti‐mouse TCRβ‐constant APC (H57‐597, Thermo Fisher Scientific) and with viability dye Aqua (Thermo Fisher Scientific). FACS samples were acquired with LSRFortessaTM (BD Bioscience) flow cytometer and FACS data analyzed with FlowJo v10 (TreeStar).

For the avidity assay, autologous activated PBMCs were electroporated with aNeon electroporation system (Thermo Fisher Scientific). Cells were resuspended at 15–20 × 10^6^ cells mL^−1^ in buffer R and mixed with 500 µg of TCRα chain RNA together with 500 µg of TCRβ chain RNA and electroporated with the following parameters: 1600 V, 10 ms, 3 pulses. The functional avidity of hCMV pp65‐specific T cells was assessed by IFN‐γ Enzyme‐Linked ImmunoSpot (ELISpot, Mabtech) assay with limiting peptide dilutions (ranging from 10 µg mL^−1^ to 0.1 pg mL^−1^) as described.^[^ ^42^ ^]^ Briefly, 2 × 10^3^ transfected T cells were plated per well in a pre‐coated 96‐well ELISpot plate (Mabtech) together with 5 × 10^4^ CD4 blasts and challenged with limiting dilutions of the specific peptide. EC_50_ values were derived by dose‐response curve analysis (log(peptide concentration) versus response) using GraphPad Prism software (v.7, GraphPad). The peptide concentration required to achieve a half‐maximal cytokine response (EC_50_) was determined and referred to as the functional avidity.

### Selection of Tumor Antigens

NeoDisc was used for the selection of tumor antigens as previously described.^[^ ^29^ ^]^

## Conflict of Interest

VZ is consultant for Cellestia Biotech. GC has received grants, research support or is coinvestigator in clinical trials by Bristol‐Myers‐Squibb, Celgene, Boehringer Ingelheim, Tigen, Roche, Iovance and Kite. GC has received honoraria for consultations or presentations by Roche, Genentech, BMS, AstraZeneca, Sanofi‐Aventis, Nextcure and GeneosTx. GC has patents in the domain of antibodies and vaccines targeting the tumor vasculature as well as technologies related to T‐cell expansion and engineering for T‐cell therapy. GC receives royalties from the University of Pennsylvania. SB and AH have patents in technologies related to T‐cell expansion and engineering for T‐cell therapy. Other authors declare no competing interests.

## Author Contributions

M.A.S.P. and V.Z. performed conceptualization. M.A.S.P. and V.Z. (computational) and J.C., S.B., M.A., and A.H. (experimental) performed methodology. M.A.S.P., M.B., and V.Z. performed software. M.A.S.P., J.C., S.B., M.A., C.S., D.B., D.D.L., F.H., M.B.‐S., G.C., A.H., and V.Z. performed investigation. M.A.S.P., M.E.B., and F.M.R. performed data curation. M.A.S.P. and V.Z. performed wrote the original draft. M.A.S.P., J.C., S.B., D.D.L., M.B.S., G.C., A.H. and V.Z. performed wrote, reviewed, and edited the draft. M.A.S.P. and V.Z. performed visualization. A.H. and V.Z. performed supervision. V.Z., G.C., and A.H. performed funding acquisition.

## Supporting information

Supporting Information

Supporting Information

## Data Availability

All the private and public Single‐cell TCR‐seq data sets can be found in Supporting Information. TCRpcDist is a pipeline piloting different codes available as a webservice. To calculate TCR distances using TCRpcDist and or TCRpcDist‐3D, please follow the instructions in the README‐TCRpcDist‐webservice. Any additional information required to reanalyze the data reported in this paper is available from the lead contact upon request. scTCR‐Seq data are available under the NCBI Gene Expression Omnibus (GEO) accession number GSE249741.
