# Supplementary material for: Predicting Antigen‐Specificities of Orphan T Cell Receptors from Cancer Patients with TCRpcDist
Source: Adv Sci (Weinh). 2024 Aug 19;11(40):2405949. doi: 10.1002/advs.202405949 (PMC11516110; doi:10.1002/advs.202405949)
Supplement: Supplementary file 1 — Supporting Information [file ADVS-11-2405949-s002.docx]

Predicting Antigen-Specificities of Orphan T Cell Receptors from Cancer Patients with TCRpcDist

*Marta A. S. Perez^1,2^, Johanna Chiffelle^1,3^, Sara Bobisse^1,3^, Francesca Mayol-Rullan^1,2^, Marine Bugnon^1,2^, Maiia E. Bragina^1,2^, Marion Arnaud^1,3^, Christophe Sauvage ^1,3^, David Barras^1,3^, Denarda Dangaj Laniti^1,3^, Florian Huber^1,3^, Michal Bassani-Sternberg^1,3^, George Coukos^1,3,4^, Alexandre Harari^1,3^ and Vincent Zoete^1,2*^*

^1^ Ludwig Institute for Cancer Research, Lausanne Branch, Department of Oncology, Lausanne University Hospital (CHUV) and University of Lausanne (UNIL), Agora Cancer Research Center, Lausanne, Switzerland.

^2^Molecular Modeling Group, SIB Swiss Institute of Bioinformatics, University of Lausanne, Quartier UNIL-Sorge, Bâtiment Amphipole, CH-1015 Lausanne, Switzerland.

^3^Center for Cell Therapy, CHUV-Ludwig Institute, Lausanne, Switzerland

^4^Immuno-Oncology Service, Department of Oncology, Lausanne University Hospital, Lausanne, Switzerland

*** Correspondence:**Corresponding Author
[Vincent.zoete@unil.ch](mailto:Vincent.zoete@unil.ch)

# Supporting Information


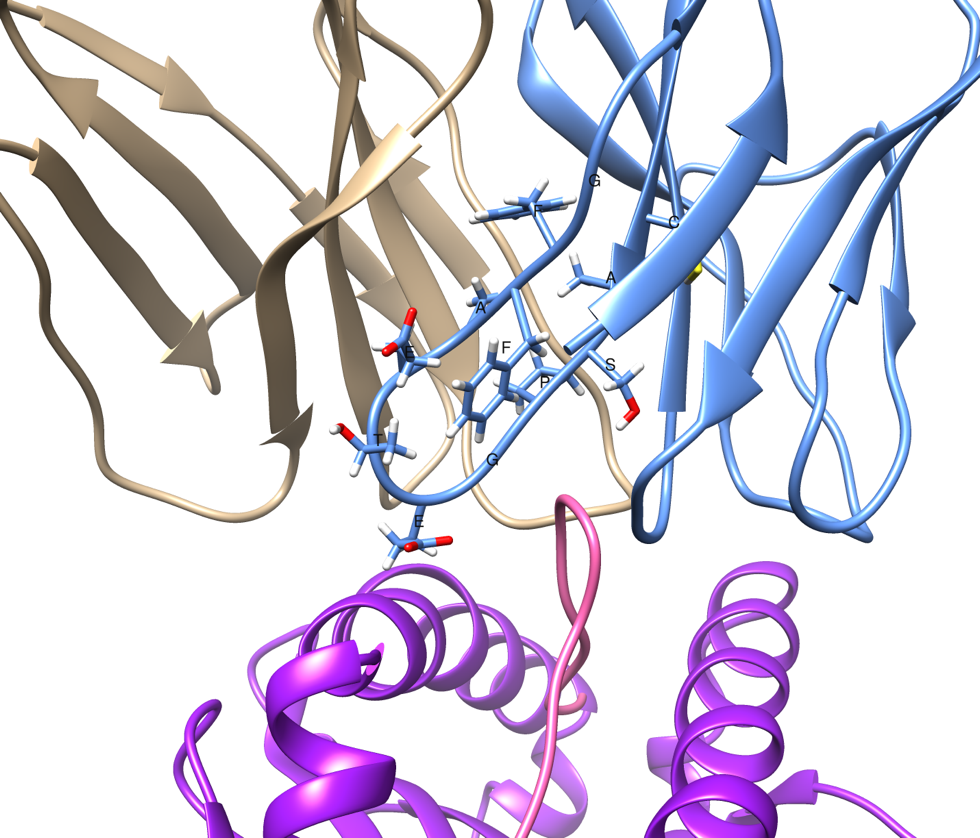


# SI Figure 1. The 3D structure of the complex TCR-pMHC PDB ID 4JRX. Representation of TCR alpha chain in brown ribbon, TCR beta chain in blue ribbon with the CDR3β residues in stick and colored by atom with carbon atoms in blue, peptide in pink ribbon and MHC in purple ribbon. CDR3β amino acids are labeled in black (single letter code). It is show that the Pro (P), the 4rd residue in the CDR3β sequence, it is in contact with the bulged portion of the peptide.

#

**SI Figure 2**. Hierarchical clustering of a set of 45 private TCRs (SI Table 2) recognizing 12 different pMHC combining TCRpcDist-3D and TCRdist3 normalized distances (between 0 and 1), each approach contributing to 50% of the final new TCR distances.


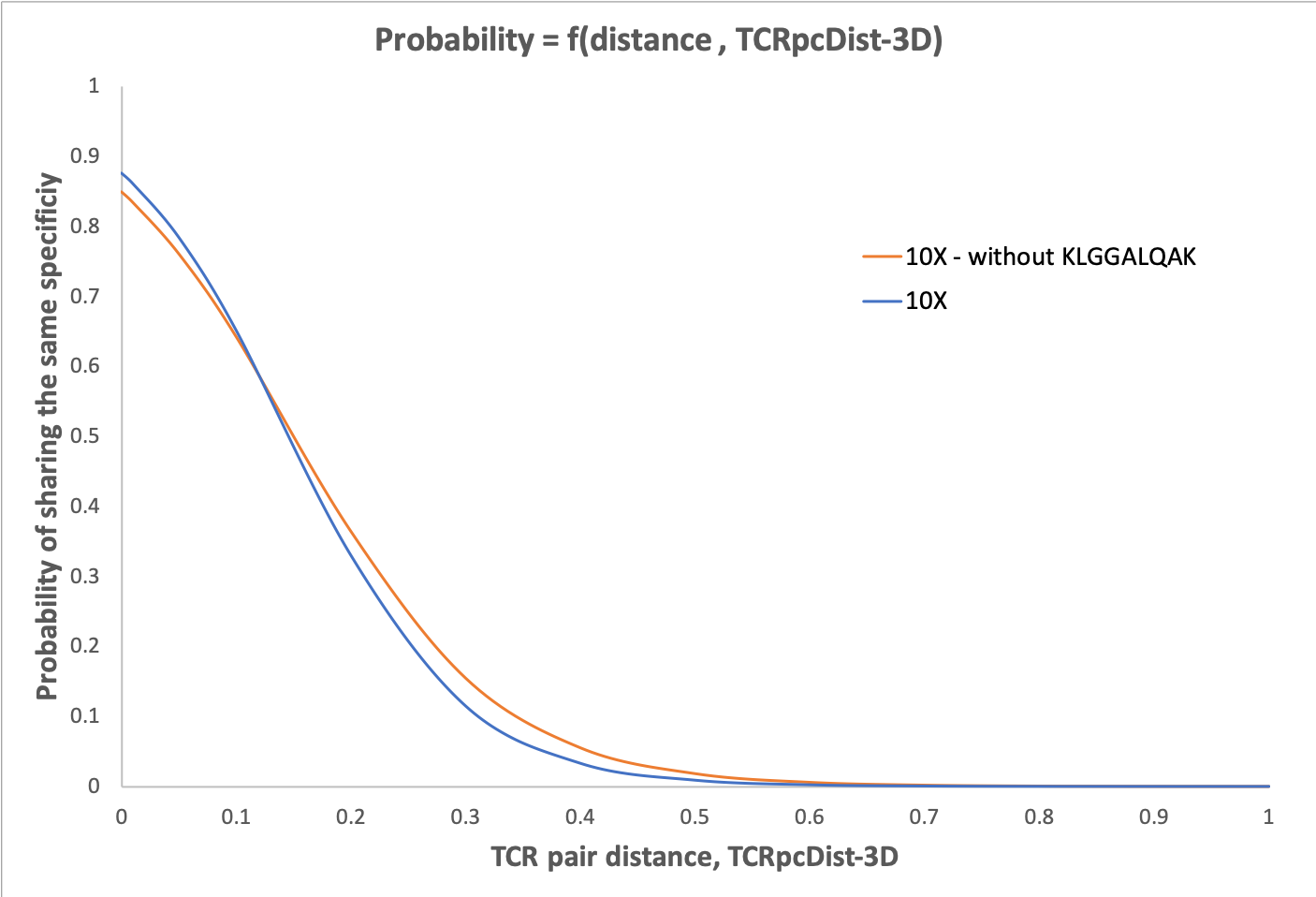


**SI Figure 3.** Probability of a TCR pair to share the same specificity as a function of their TCRpcDist-3D value. The sigmoid curve in blue was obtained using 8’528 TCRs with known specificities. The sigmoid curve in orange was obtained after removing the TCRs recognizing the overrepresented KLGGAQAK peptide.

**SI Figure 4**. Screening of viral peptide-specific TCRs predicted through TCRpcDist-3D. Two TCRs from patient Mel #5 and 1 TCR from patient GI #1 were predicted to bind the EBV BMLF1 GLCTLVAML peptide presented by HLA-A*02:01, based on the closest distance to EBV BMLF1-specific TCRs of the private and 10X Genomics databases. Jurkat cells were transfected with RNA coding for candidate or control TCRs and co-cultured with GLCTLVAML peptide-pulsed matched presenting cells. Luminescence was measured after overnight incubation. Ctrl + TCR: positive control TCR specific for the tested peptide. Mock: Jurkat cells transfected with water. αCD3/CD28: Jurkat cells stimulated with TransAct^TM^.

**SI Figure 5**. Screening of neoAg and TAA-specific TCRs predicted through TCRpcDist-3D. A-B) Candidate TCRs were predicted by TCRpcDist-3D to bind specific neoAgs identified in cancer patients using private data. A) In patient Lung #1, three additional TCRs showing the closest distance to previously deorphanized TCRs were revealed specific for the neoAg DSNDYHILR/HLA-A*68:01 by Luminescence assay following RNA-TCR transfection into Jurkat cells and overnight coculture with matched peptide-pulsed presenting cells. B) In patient Mel #6, 1 TCR was tested and found specific for the neoAg SLKLHYQL/HLA-B*08:01. C) Four TCRs derived from patient Mel #6 were predicted and screened toward the TAA ELAGIGILTV/HLA-A*02:01 peptide based on their closest distance to TCRs with the same specificity. Two out of 4 TCRs were shown to mediate T cell activation and confirmed the predicted specificity by Luminescence assay. Ctrl + TCR: positive control TCR specific for the tested peptide. Mock: TCR/KO Jurkat cells transfected with water. αCD3/CD28: Jurkat cells stimulated with TransAct^TM^.

**Data S1 -** Solvent accessibility calculations for the CDR loops of the PDB structures.

**Data S2 -** Reevaluation of TCRpcDist using a larger set of 96 TCRs structures retrieved from Protein Data Bank that recognize 48 distinct known pMHCs.

**Data S3 -** Three independent TCRpcDist-3D distance calculations for the private set of 45 TCRs (part 1) and their standard deviations SD (part 2). We observe that among the three independent runs there are only 94 TCR pairs distances with SD>0.1 and the maximal SD is 0.19. This indicates that only 47 pairs of TCRs among the 990 possible pairs report SD>0.1, i.e. 4.7%. The average SD over all the pairs is only 0.04, indicating that the variability on calculated TCRpcDist-3D distances between several runs, starting from the same input, remains very limited and does not change the main conclusions of TCR distance analysis.

**Data S4** – Further details on the performance comparison between TCRpcDist and the following approaches: TCRbase, TCRdist3, TCRbase 1.0, NetTCR-2.2 and SwarmTCR.

**Data S5 -** By excluding TCRs with SP mutations from the initial developmental set of 54 PDB structures, we refined the set to 48 TCRs. The PDB identifiers excluded from the original set, as detailed in SI Table 1, were 2P5W, 2PYE, 2VLR, 3MV8, 3MV9 and 5NQK. Benchmarking our approach using this set of 48 structures yielded consistent conclusions regarding the optimal combination of parameters for our methodology. Detailed analysis and comparisons between the original set of 54 and the refined set of 48, using additional standard metrics beyond those presented in the current manuscript, are provided in this document.

**Data S6** – Assessment set of 374 non-redundant TCRs taken from VDJdb in 18.09.2020 (TCRs described in **SI Table 12.** Similarly to the results obtained for the developmental set: (i) using only the residues of CDR1s and CDR2s (α and β) with nSESA > 5% and CDR3s (α and β) with nSESA>20% was found to improve the quality of the clustering approach; (ii) we found that the best clustering was obtained when giving a weight of 10% to the contributions of CDR1s and CDR2s (α and β) and of 30% to those of CDR3s (α and β) in the calculation of the distance. The hierarchical clustering trees for this set is depicted as the tables that describe the weights exploration. Again, we measured the quality of the clustering by color change and pMHC-distance. We observed that the number of color changes was 198 using only CDR3b, 157 using all CDRs and 154 using all CDRs + nSESA from the 3D structure (p-value <0.0001 in all cases). The pMHC-distance was 0.87, 0.57 and 0.55 for these three TCRpcDist variants, respectively. Once again, we observed an increase of the clustering efficiency using TCRpcDist-3D and the parameters obtained in the developmental set.
