# Supplementary material for: Predicting Antigen‐Specificities of Orphan T Cell Receptors from Cancer Patients with TCRpcDist
Source: Adv Sci (Weinh). 2024 Aug 19;11(40):2405949. doi: 10.1002/advs.202405949 (PMC11516110; doi:10.1002/advs.202405949)
Supplement: Supplementary file 2 — Supporting Information [file ADVS-11-2405949-s001.zip › SI-corrected/DataS1.docx]

Predicting Antigen-Specificities of Orphan T Cell Receptors from Cancer Patients with TCRpcDist

*Marta A. S. Perez^1,2^, Johanna Chiffelle^1,3^, Sara Bobisse^1,3^, Francesca Mayol-Rullan^1,2^, Marine Bugnon^1,2^, Maiia E. Bragina^1,2^, Marion Arnaud^1,3^, Christophe Sauvage ^1,3^, David Barras^1,3^, Denarda Dangaj Laniti^1,3^, Florian Huber^1,3^, Michal Bassani-Sternberg^1,3^, George Coukos^1,3,4^, Alexandre Harari^1,3^ and Vincent Zoete^1,2*^*

^1^ Ludwig Institute for Cancer Research, Lausanne Branch, Department of Oncology, Lausanne University Hospital (CHUV) and University of Lausanne (UNIL), Agora Cancer Research Center, Lausanne, Switzerland.

^2^Molecular Modeling Group, SIB Swiss Institute of Bioinformatics, University of Lausanne, Quartier UNIL-Sorge, Bâtiment Amphipole, CH-1015 Lausanne, Switzerland.

^3^Center for Cell Therapy, CHUV-Ludwig Institute, Lausanne, Switzerland

^4^Immuno-Oncology Service, Department of Oncology, Lausanne University Hospital, Lausanne, Switzerland

*** Correspondence:**Corresponding Author
[Vincent.zoete@unil.ch](mailto:Vincent.zoete@unil.ch)

# Supporting Information

**Data S1 - solvent accessibility calculations for the CDR loops of the PDB structures.**

**Table_nSESA_CDR1a**

TYSDRGSQSFF 1ao7 0.615965 0.168271 0.694824 0.493733 0.930795 0.772578 0.084069 0.845101 0.276231 0.0190804 0.0517796

DYTNSMFDYFL 1bd2 0.61813 0.357427 0.783898 0.315726 1.12295 0.792033 0.147975 0.715555 0.386863 0.0444302 0.00000094

NHSTISGTDYIH 1mi5 0.672392 0.0917007 0.851623 0.992169 0.340374 0.545135 0.892897 0.869298 0.102443 0.481567 0.00000095 0.338571

NSSSVFSSLQ 1oga 0.593988 0.409275 0.980159 0.493847 0.6814 0.108468 0.871238 0.195293 0.10227 0.00000094

VYETRDTTYYLF 2ak4 0.717129 0.215747 0.551514 0.593851 0.341231 0.674197 0.996021 0.618895 0.197112 0.253778 0.0228386 0.178327

KYSYSATPYLF 2e7l 0.699142 0.320726 0.912064 0.436131 1.01718 0.797506 0.788392 0.368664 0.568912 0.00000094 0.0391187

NHSTISGNEYVY 2esv 0.69957 0.243082 0.870051 1.10474 0.445754 0.48838 0.982859 0.681275 0.139556 0.498984 0.0721807 0.208604

SFTDSAIYNLQ 2f53 0.668388 0.147431 0.798028 0.356099 0.578186 0.813841 0.226127 0.439105 0.261269 0.00000094 0.236437

KYSYSATPYLF 2oi9 0.703366 0.25794 0.755092 0.481907 1.0988 0.717899 0.74934 0.32549 0.538795 0.00000094 0

SFTDSAIYNLQ 2p5e 0.68655 0.283926 0.81997 0.500611 0.845563 0.672237 0.252754 0.554677 0.161561 0.10617 0.212956

SFTDSAIYNLQ 2p5w 0.659719 0.285793 0.850436 0.589993 0.843694 0.700988 0.248121 0.548041 0.179725 0.121004 0.223723

SFTDSAIYNLQ 2pye 0.606992 0.16039 0.758881 0.47198 0.715286 0.67876 0.0222597 0.679448 0.167283 0.00000094 0.168086

NSSSVFSSLQ 2vlr 0.718289 0.404399 1.00314 0.582508 0.701088 0.139931 0.924082 0.231636 0.113602 0.00000094

KYSYSATPYLF 3e2h 0.731217 0.254004 0.773595 0.656466 1.04605 0.617438 0.878535 0.335942 0.611684 0.427559 0.141739

KYSYSATPYLF 3e3q 0.617279 0.291325 0.801063 0.458859 1.04245 0.737002 0.793678 0.330027 0.486399 0.00000094 0.0897902

TYDTSDPSYGLF 3ffc 0.701123 0.112648 0.79187 0.27433 1.04757 0.382746 0.993686 0.412736 0.0893075 0.00000212 0 0

SFPSSNFYALH 3gsn 0.556935 0.151253 0.68795 0.576751 0.846303 0.878215 0.301781 0.915631 0.109098 0.00000094 0.155162

TYSDRGSQSFF 3hg1 0.661419 0.158055 0.751079 0.556743 0.841984 0.678566 0.142816 0.847385 0.139019 0.00000080 0

SYTVSGLRGLF 3mv7 0.555269 0.242487 0.725344 0.485107 0.924634 0.384186 0.39728 0.599529 0.00000212 0.0638525 0.0896804

SYTVSGLRGLF 3mv8 0.514 0.315734 0.775807 0.471571 0.894647 0.377312 0.43989 0.610782 0.00000212 0.0639979 0.0681802

SYTVSGLRGLF 3mv9 0.617374 0.323536 0.828617 0.526827 0.971359 0.434394 0.411418 0.535446 0.00000212 0 0.0361611

TYSDRGSQSFF 3qdg 0.648525 0.11072 0.722896 0.515829 0.891851 0.868258 0.175929 0.923146 0.247788 0.0677723 0.255764

TSSSIFNTWL 3qdm 0.569646 0.4111 0.938886 0.631249 0.691341 0.157127 0.84914 0.173222 0.00000067 0.0297647

TYSNSASQSFF 3sjv 0.518348 0.189027 0.819917 0.701733 0.805909 0.889158 0.383632 0.703129 0.193611 0.164068 0.156457

NYSYGATPYLF 3vxm 0.604049 0.410399 0.728718 0.742129 1.08602 0.745594 0.838988 0.445064 0.511179 0.125107 0.0786879

SFTDSAIYNLQ 3vxr 0.465302 0.164521 0.786852 0.579148 0.720812 0.698266 0.00000095 0.667826 0.13927 0.0563695 0.0720967

TYSDRGSQSFF 3vxu 0.931178 0.33434 0.884834 0.701898 0.987389 0.883623 0.26108 0.690455 0.563042 0.00000080 0.0830573

TYSDRGSQSFF 4ftv 0.681333 0.18552 0.78056 0.66685 0.86646 0.734793 0.197191 0.766093 0.302991 0.0857342 0.0726363

VYETRDTTYYLF 4jrx 0.713032 0.136803 0.765995 0.579915 1.04415 0.693538 0.921662 0.927993 0.195089 0.209488 0.046985 0.06006

NYSTTSDRLY 4jry 0.620893 0.194692 0.605839 0.501145 0.847998 0.510921 0.682718 0.294004 0.00000094 0.119768

TYSYRGSQSFF 4l3e 0.712365 0.245635 0.696322 0.533107 0.982135 0.816241 0.33247 0.847295 0.201831 0.121673 0.161054

NFSDSVNNLW 4mnq 0.520567 0.188676 0.586265 0.470552 0.50584 0.697811 0.261802 0.00000110 0 0.00000067

SFPSSNFYALH 5d2l 0.563296 0.15314 0.384169 0.699481 0.770289 0.838464 0.292108 0.818351 0.0836221 0.00000094 0.220852

NHSTISGTDYIH 5d2n 0.678588 0.0911631 0.769697 0.860916 0.358143 0.615489 1.00117 0.821795 0.230269 0.467371 0.0784684 0.147499

TSSSIFNTWL 5e6i 0.631072 0.256016 0.97789 0.544512 0.759049 0.165007 0.825899 0.565349 0.00000067 0.413285

TYSDRGSTSFF 5e9d 0.660819 0.153795 0.831965 0.663122 0.804137 0.684247 0.268327 0.489123 0.218848 0.00000080 0.266362

NSSSVFSSLQ 5euo 0.531787 0.254949 0.810575 0.415799 0.444036 0.0233697 0.716307 0.21332 0.00000094 0

SFPSSNFYALH 5isz 0.591676 0.175142 0.45692 0.876887 0.80696 0.789317 0.417438 0.660235 0.154361 0.00000094 0

TYDTSESDYYLF 5jhd 0.677047 0.295229 0.803152 0.586418 1.02412 0.677012 0.941449 0.488945 0.188627 0.248477 0.217643 0.135738

TYDTSESDYYLF 5jzi 0.652304 0.277033 0.799095 0.452774 1.02041 0.672463 0.878309 0.939187 0.284458 0.253322 0.253629 0

NFSDSVNNLQ 5men 0.578952 0.0686896 0.722288 0.464256 0.461487 0.295394 0.712815 0.252723 0.00000094 0

TYSDRGSQSFF 5nht 0.410048 0.25289 0.663447 0.690051 0.874715 0.785478 0.387625 0.749933 0.107001 0.00000080 0.0427386

TYSDRGSQSFF 5nqk 0.653748 0.377603 0.929049 0.622535 0.983923 0.814639 0.369095 0.809865 0.110702 0.202333 0.0463799

SYEDSTFNYFP 5sws 0.604438 0.310806 0.788954 0.545292 0.903265 0.805781 0.181845 0.557936 0.567623 0.0260338 0.0802112

SYEDSTFNYFP 5swz 0.565751 0.248263 0.794766 0.415243 0.918926 0.702888 0.114992 0.430167 0.363684 0.00000080 0

TYSDSASNYFP 5tez 0.621414 0.126887 0.791797 0.49776 0.891751 0.850319 0.146536 0.644487 0.142474 0.00000080 0

TSSSIFNTWL 5w1v 0.697276 0.216343 0.890226 0.648443 0.518822 0.125807 0.629588 0.420084 0.0617069 0.412167

LYETSWWSYYIF 5xot 0.728629 0.214568 0.713086 0.367917 1.10846 0.658219 0.955893 0.575564 0.333471 0.342394 0.37592 0.0680187

LYETSWWSYYIF 5xov 0.698641 0.227307 0.714151 0.327047 1.02434 0.677529 1.04164 0.844364 0.333509 0.430435 0.24169 0.169457

TYDTSENDYILF 5yxu 0.628229 0.253791 0.621964 0.141788 0.923373 0.650619 0.756361 0.93483 0.354328 0.272134 0.16588 0.0410666

SHNNIATNDYIT 6avf 0.715536 0.106189 0.875233 1.1406 0.388403 0.526656 0.883484 0.902285 0.125804 0.64573 0.00000095 0.0664768

SHNNIATNDYIT 6avg 0.629572 0.0407823 0.885351 0.953205 0.268279 0.614699 0.896412 0.852442 0.23216 0.561139 0.00000095 0.0949078

VYETRDTTYYLF 6bj2 0.662696 0.313268 0.619978 0.681489 1.04241 0.757317 0.872974 0.81503 0.213294 0.442171 0.106724 0.0674883

TYSYRGSQSFF 6dkp 0.665824 0.191865 0.666921 0.579854 0.785179 0.731862 0.145435 0.859815 0.391153 0.183137 0.283774

**Table_nSESA_CDR1b**

AQDMNHEYMS 1ao7 0.489733 0.11988 1.00712 0.602572 1.19449 0.20198 0.534645 0.194618 0.00000089 0.165296

AQDMNHEYMS 1bd2 0.533427 0.118106 0.690737 0.518946 1.03023 0.207932 0.364145 0.049851 0.00000089 0

DPISGHVSLF 1mi5 0.5571 0.398667 0.507215 0.99036 1.10265 0.429249 0.572784 0.0581229 0.00000094 0

EQNLNHDAMY 1oga 0.590213 0.390665 0.852853 0.525716 0.92047 0.217321 0.497413 0.134632 0.233675 0.00000076

AQDMNHNSMY 2ak4 0.426438 0.142847 0.864494 0.402493 0.872816 0.336116 0.640506 0.346241 0.00000089 0.113611

NQTNNHNNMY 2e7l 0.54866 0.103884 0.87608 0.479962 0.903058 0.268156 0.685632 0.494031 0.00000089 0.171052

DPISGHDNLY 2esv 0.646245 0.253262 0.462256 0.942483 1.0398 0.381539 0.62631 0.174318 0.00000094 0

AQDMNHEYMS 2f53 0.559075 0.0966798 0.771768 0.367848 0.860114 0.122852 0.483088 0.00000076 0 0.00000145

NQTNNHNNMY 2oi9 0.554333 0.13706 0.856849 0.505218 0.888264 0.296623 0.692623 0.470098 0.00000089 0.109636

AQDMNHEYMS 2p5e 0.551884 0.172553 0.918627 0.3927 0.844589 0.0480453 0.525751 0.00000076 0 0.00000145

AQDMNHEYMS 2p5w 0.539108 0.222559 0.852069 0.339479 1.00654 0.0465228 0.544876 0.00000076 0 0.00000145

AQDMNHEYMS 2pye 0.578264 0.218356 0.832582 0.34245 0.950091 0.0488323 0.515827 0.117797 0.00000089 0.0768417

EQNLNHDAMY 2vlr 0.614451 0.235453 0.915625 0.5562 0.929154 0.281188 0.618379 0.00000164 0 0.00000076

NQTNNHNNMY 3e2h 0.514585 0.120643 0.860534 0.42819 0.802585 0.299103 0.704696 0.371694 0.00000089 0.071564

NQTNNHNNMY 3e3q 0.48952 0.0451884 0.865084 0.396454 0.861489 0.269835 0.67253 0.390587 0.00000089 0.111694

NPISGHATLY 3ffc 0.515111 0.30801 0.400774 0.864174 1.08632 0.279375 0.544842 0.00000121 0 0.131362

AQDMNHEYMS 3gsn 0.462126 0.0943442 0.93961 0.437998 0.967733 0.234632 0.594259 0.11585 0.00000089 0

TVEGTSNPNLY 3hg1 0.425362 0.0349874 0.605924 0.891162 0.543231 0.667004 0.777731 0.0408043 0.251904 0.00000094 0.264644

SPRSGDLSVY 3mv7 0.390101 0.385126 0.402383 1.07863 1.10921 0.283334 0.516804 0.15119 0.064779 0.0472612

SPRSGDLSVY 3mv8 0.54065 0.373021 0.451412 1.07392 1.09836 0.270246 0.546722 0.176187 0.0710419 0.0814454

SPRSGDLSVY 3mv9 0.516538 0.420461 0.422585 1.08711 1.0155 0.178019 0.569265 0.290364 0.00000111 0.224219

TQDMRHNAMY 3qdg 0.545507 0.101848 0.864493 0.558501 0.999674 0.15676 0.598101 0.288258 0.00000089 0.117794

HQTENHRYMY 3qdm 0.678538 0.033518 0.984001 0.718634 0.722019 0.967108 0.291691 0.366053 0.00000089 0.109811

AQDMNHEYMY 3sjv 0.579613 0.271537 0.805425 0.686511 0.767503 0.178586 0.590448 0.381123 0.0632873 0.178951

EQHMGHRAMY 3vxm 0.548808 0.172884 0.890671 0.38679 0.99544 0.317506 0.625731 0.214296 0.332162 0.0768655

DPISEHNRLY 3vxr 0.507445 0.426057 0.435552 0.896568 1.07102 0.400201 0.472363 0.262245 0.00000094 0.0422074

SQNMNHEYMS 3vxu 0.47515 0.173399 0.906505 0.396029 0.795792 0.185617 0.519056 0.263043 0.00000089 0

AQDMNHEYMS 4ftv 0.517375 0.345991 0.879605 0.591518 1.09297 0.178571 0.4616 0.216018 0.00000089 0.251569

AQDMNHNSMY 4jrx 0.54215 0.197961 1.03732 0.448209 0.886822 0.33612 0.548454 0.292564 0.00000089 0.115403

SPKSGHDTVS 4jry 0.499762 0.405186 0.532676 0.875864 0.926439 0.241218 0.422287 0.056212 0.00000111 0.0575069

TQDMRHNAMY 4l3e 0.60119 0.10586 0.817642 0.436786 0.867686 0.128625 0.622121 0.237072 0.00000089 0.150542

AQDMNHEYMS 4mnq 0.558346 0.22578 1.15146 0.590513 0.952358 0.150155 0.579236 0.421868 0.00000089 0.0422665

DPISGHTALY 5d2l 0.279708 0.243891 0.463575 0.997348 0.916559 0.408221 0.854342 0.253104 0.374568 0.166273

DPISGHVSLY 5d2n 0.525943 0.223288 0.341574 0.865524 1.01715 0.281661 0.427922 0.0766831 0.00000094 0.1185

SQNMNHEYMS 5e6i 0.692428 0.628228 1.00366 0.567448 1.11849 0.501925 0.636962 0.0530984 0.351267 0.00000145

AQDMNHEYMA 5e9d 0.534718 0.0629738 0.876057 0.566261 0.982528 0.19796 0.745115 0.337337 0.00000089 0

EQNLNHDAMY 5euo 0.515444 0.246321 0.869165 0.535023 0.865044 0.158032 0.486236 0.00000164 0.0565983 0.0324785

EQNLNHDAMY 5isz 0.621096 0.237155 0.817117 0.430237 0.902579 0.170218 0.644632 0.193472 0.0258515 0.209053

EQNLNHDAMY 5jhd 0.802662 0.225284 0.801061 0.471512 0.863968 0.22834 0.683478 0.293874 0.00000089 0.117674

SQTMGHDKMY 5jzi 0.506167 0.174723 0.704259 0.468855 0.989935 0.289822 0.782088 0.394481 0.00000089 0.0656153

AQDMNHEYMS 5men 0.488951 0.130528 0.940702 0.562651 0.977174 0.153344 0.570713 0.400377 0.00000089 0.103382

EQNLNHDAMY 5nht 0.800412 0.213044 0.826913 0.525396 0.652071 0.115738 0.335671 0.00000164 0 0.00000076

EQNLNHDAMY 5nqk 0.736182 0.204964 0.829617 0.553947 0.833657 0.127303 0.37417 0.118335 0.0386555 0.0439709

SQTMNHDTMY 5sws 0.526798 0.228922 0.958229 0.531792 0.981892 0.260364 0.570564 0.00000121 0.0448898 0.0471168

SQTMNHDTMY 5swz 0.438734 0.168764 1.04858 0.533634 0.872829 0.198605 0.61046 0.00000121 0 0.00000076

SQNMNHEYMS 5tez 0.540392 0.190261 0.883376 0.462559 0.948551 0.160611 0.657452 0.571764 0.00000089 0.0910645

SPRSGDLSVY 5w1v 0.277837 0.383215 0.537273 1.04846 0.731198 0.0672497 0.310755 0.00000145 0 0.109934

AQDMNHNSMY 5xot 0.427339 0.100926 0.769186 0.506899 0.875335 0.262014 0.4602 0.00000145 0 0.00000076

TVEGTSNPNLY 5xov 0.589568 0.151735 0.656785 0.883197 0.395188 0.825188 0.814668 0.133392 0.307742 0.121966 0.194535

SQTMGHDKMY 5yxu 0.476957 0.0929765 0.703465 0.4609 1.01662 0.197943 0.699923 0.345126 0.00000089 0

VQDMDHENMF 6avf 0.505144 0.158007 0.854145 0.426769 0.858134 0.168017 0.431559 0.270306 0.00000089 0

SPRSGDLSVY 6avg 0.418692 0.408266 0.231113 0.46225 0.756844 0.0754144 0.421905 0.159629 0.00000111 0.26205

SSQSGHNTVS 6bj2 0.636046 0.805787 0.501819 1.02622 0.998417 0.382619 0.473989 0.157942 0.133432 0.0316672

TQDMRHNAMY 6dkp 0.579475 0.0475241 0.844738 0.607743 0.890923 0.104617 0.667579 0.134044 0.0334607 0.122867

**Table_nSESA_CDR2a**

MSIYSNGDK 1ao7 0.193122 0.36347 0.189907 0.812915 0.801956 0.683898 0.642416 0.879374 0.589997

ISISSIKD 1bd2 0.171234 0.377236 0.108938 0.742729 0.478018 0.964807 0.329164 0.832743

IHGLTSNV 1mi5 0.17544 0.55746 0.149987 0.485055 0.797379 0.838705 0.835471 0.447987

VTVVTGGEVK 1oga 0.203469 0.289646 0.136327 0.642039 0.654493 0.634596 0.485061 0.400761 0.742987 0.494858

IRRNSFDEQNE 2ak4 0.191414 0.493275 0.158272 0.290505 0.211893 0.795433 0.582917 0.473359 0.766867 0.642315 0.673425

LKYYSGDPVVQ 2e7l 0.0881218 0.605765 0.236983 0.701641 0.852846 0.867343 0.80033 0.538179 0.685896 0.320183 0.84566

IHGLKNNE 2esv 0.110054 0.496375 0.189173 0.515557 0.844734 0.735727 0.749707 0.676646

LLIPFWQREQ 2f53 0.140029 0.268118 0.00000095 0.0377699 0.664292 0.67816 0.577875 0.82346 0.818262 0.471403

LKYYSGDPVVQ 2oi9 0.101157 0.570313 0.247339 0.695485 0.850483 0.869554 0.802189 0.531832 0.634683 0.309312 0.854213

LLITPWQREQ 2p5e 0.190391 0.216335 0.166752 0.335436 0.472219 0.857761 0.566428 0.583108 0.924213 0.494391

LLITPWQREQ 2p5w 0.211036 0.355431 0.1463 0.399441 0.578904 0.839661 0.851302 0.600688 0.847385 0.576433

LLIQSSQREQ 2pye 0.146127 0.271465 0.00000095 0.576721 0.60323 0.991763 0.613384 0.827836 0.86514 0.485176

VTVVTGGEVK 2vlr 0.167162 0.34827 0.215797 0.674895 0.657695 0.637776 0.64433 0.439864 0.74422 0.53302

LKYYSGDPVVQ 3e2h 0.101429 0.598303 0.339003 0.729931 0.915301 0.890502 0.788649 0.535636 0.691884 0.229527 0.869701

LKYYSGDPVVQ 3e3q 0.092913 0.599704 0.240029 0.701396 0.834231 0.91899 0.81327 0.581297 0.61732 0.267343 0.860275

IYQGSYDQG 3ffc 0.12393 0.372014 0.105629 0.0635205 0.287142 0.807356 0.697039 1.04765 0.900421

FVMTLNGDEK 3gsn 0.210979 0.640516 0.0608279 0.761075 0.750509 0.714466 0.968082 0.820028 0.841721 0.623827

MFIYSNGDK 3hg1 0.205325 0.344012 0.181224 0.790859 0.771233 0.736171 0.662671 0.868227 0.604258

FTLYSAGEEK 3mv7 0.229765 0.211524 0.00000094 0.777628 0.611971 0.928436 0.957407 0.506329 0.77298 0.54227

FTLYSAGEEK 3mv8 0.263545 0.185122 0.0626304 0.736563 0.66606 0.965997 0.91883 0.459282 0.840501 0.530719

FTLYSAGEEK 3mv9 0.246601 0.219283 0.00000094 0.68889 0.666623 0.829055 0.919392 0.434831 0.713654 0.538231

MFIYSNGDK 3qdg 0.368049 0.49685 0.287912 0.724976 0.827698 0.799659 0.546426 0.924911 0.620759

IALYKAGELT 3qdm 0.252157 0.576853 0.00000094 0.798135 0.779219 0.743724 0.564378 0.890884 0.593166 0.896711

MSVYSSGN 3sjv 0.184706 0.419601 0.107555 0.683337 0.871542 0.735274 0.947077 0.560106

LKYFSGDTLVQ 3vxm 0.0929251 0.564931 0.213291 0.742962 0.6319 0.758071 0.87498 0.5849 0.727484 0.26011 0.903381

LLIQSSQREQ 3vxr 0.271578 0.348408 0.0579601 0.493799 0.591009 1.00546 0.616336 0.911101 0.760287 0.475706

MSIYSNGDK 3vxu 0.197333 0.579214 0.148367 0.682927 0.908806 0.766103 0.599555 0.941403 0.771281

MSIYSNGDK 4ftv 0.296022 0.368741 0.319345 0.755529 0.777483 0.820262 0.637825 0.944529 0.599295

IRRNSFDEQNE 4jrx 0.237467 0.419828 0.201898 0.340568 0.095986 0.777541 0.643322 0.989849 1.04527 0.795949 0.62421

FVLLSNGAVK 4jry 0.199602 0.314801 0.0756353 0.741027 0.670809 0.688749 0.816233 0.455264 0.736132 0.631087

MFIYSNGDK 4l3e 0.396133 0.443685 0.240617 0.668717 0.767556 0.750992 0.607618 0.896728 0.605833

FYIPSGTK 4mnq 0.180426 0.447664 0.00000095 0.526923 0.73795 0.881649 0.616883 0.920571

FVMTLNGDEK 5d2l 0.234469 0.545867 0.00000089 0.653359 0.722632 0.705101 0.822217 0.533435 0.93946 0.709687

IHGLTSNV 5d2n 0.0445245 0.489459 0.219537 0.507893 0.825348 0.790413 0.906248 0.440593

IALYKAGELT 5e6i 0.393523 0.781999 0.191782 0.432348 0.842627 0.785662 0.572392 0.848278 0.684551 0.956082

MSIYSNGDK 5e9d 0.135374 0.404596 0.118372 0.70259 0.810567 0.784137 0.61684 0.884475 0.624015

VTVVTGGEVK 5euo 0.251637 0.313223 0.127537 0.666859 0.672849 0.655804 0.735649 0.420537 0.761039 0.568342

FVMTLNGDEK 5isz 0.20876 0.423611 0.282739 0.826407 0.77722 0.726986 0.866011 0.97239 0.758647 0.52731

IRQEAYKQQ 5jhd 0.370731 0.588749 0.395173 0.49817 0.3644 0.907905 0.821141 0.936776 0.759672

IRQEAYKQQ 5jzi 0.551666 0.542747 0.390283 0.281369 0.513763 0.823642 0.643712 0.977232 0.927309

FYIPSGTK 5men 0.184228 0.391892 0.072213 0.519891 0.889819 0.669184 0.915906 0.638748

MSIYSNGDK 5nht 0.109514 0.347018 0.165633 0.693278 0.664214 0.591344 0.44614 0.704845 0.611737

MSIYSNGDK 5nqk 0.0967799 0.528861 0.181981 0.71572 0.908506 0.867763 0.636488 0.897237 0.567675

ISIRSVSDKK 5sws 0.262452 0.416023 0.14918 0.760765 0.615908 0.893511 0.778522 0.795934 0.725593 0.533645

ISIRSVSDKK 5swz 0.223057 0.324587 0.0777709 0.856702 0.588855 0.975514 0.663427 0.809051 0.659705 0.702588

IDIRSNVGEK 5tez 0.194434 0.329648 0.0677698 0.6415 0.556728 0.916203 0.595922 0.724652 0.778392 0.568329

IALYKAGELT 5w1v 0.141342 0.577452 0.00000094 0.834276 0.757288 0.593757 0.955193 0.513901 0.80314 0.548857

IRQGSDEQ 5xot 0.235625 0.539419 0.271775 0.252351 0.444779 1.00323 0.759981 0.921709

IRQGSDEQ 5xov 0.185823 0.65391 0.225811 0.154512 0.374541 1.10107 0.776389 0.961038

IRQEAYKQQ 5yxu 0.241241 0.610909 0.308916 0.479902 0.413853 0.952412 0.712272 1.03541 0.902492

IQGYKTKV 6avf 0.0727794 0.511793 0.245399 0.564306 0.738413 0.923049 0.738615 0.50257

IQGYKTKV 6avg 0.0843115 0.523826 0.179882 0.51556 0.803779 0.792323 0.728211 0.420892

IRRNSFDEQNE 6bj2 0.253971 0.578991 0.125975 0.272455 0.480005 0.879753 0.538744 0.733952 0.47321 0.608495 0.618608

MFIASNGDK 6dkp 0.291199 0.689084 0.336964 0.666412 0.835289 0.733214 0.636819 0.917253 0.557802

**Table_nSESA_CDR2b**

HYSVGAGITD 1ao7 0.256784 0.369284 0.0893217 0.509312 0.308057 0.770556 1.00382 0.629104 0.817543 0.693463

HYSVGAGITD 1bd2 0.227472 0.399067 0.04686 0.554533 0.196622 0.844488 0.956756 0.617792 0.653512 0.694202

TYFQNEAQLDK 1mi5 0 0.162049 0.0644559 0.630191 0.536385 0.701071 0.517888 0.591784 0.75174 0.346498 0.800622

YYSQIVNDFQ 1oga 0.196963 0.11525 0.00000145 0.507289 0.568076 0.551554 0.901365 0.588441 0.636409 0.500753

YYSASEGTTD 2ak4 0.248699 0.190438 0.00000145 0.249017 0.499717 0.742768 0.845618 0.53758 0.626289 0.546605

YYSYGAGSTE 2e7l 0.181056 0.22013 0.0938766 0.712772 0.37889 0.802545 1.01663 0.395218 0.665641 0.380261

LHFVKESKQDE 2esv 0.150974 0.00000090 0.0652855 0.518306 0.737012 0.74286 0.619584 0.699344 0.648306 0.401738 0.741529

HYSVSVGMTD 2f53 0.221104 0.160053 0.00000145 0.391948 0.544851 0.711503 0.987157 0.439508 0.659832 0.563175

YYSYGAGSTE 2oi9 0.169242 0.280185 0.00000145 0.71981 0.383412 0.803372 1.02802 0.295263 0.6353 0.37962

HYSVAIQTTD 2p5e 0.201372 0.215939 0.0830921 0.55801 0.504664 0.687064 0.932585 0.46837 0.68046 0.704096

HYSVSVGMTD 2p5w 0.195025 0.169175 0.00000145 0.403376 0.572451 0.742488 1.03198 0.440333 0.697924 0.617959

HYSVGAGTTD 2pye 0.204611 0.166696 0.0436849 0.627857 0.358051 0.729403 0.946896 0.702218 0.68199 0.708416

YYSQIVNDFQ 2vlr 0.238969 0.118709 0.0291584 0.601072 0.60784 0.615831 0.897352 0.711417 0.71049 0.591489

YYSYGAGSTE 3e2h 0.152618 0.230626 0.00000145 0.733879 0.47783 0.832692 0.956332 0.491474 1.12653 0.402869

YYSYGAGSTE 3e3q 0.167941 0.327745 0.10893 0.741524 0.379431 0.799375 1.01539 0.328917 0.68125 0.400037

IQFQNNGVVDD 3ffc 0.102659 0.183093 0.0738931 0.604858 0.584968 0.762365 0.528324 0.715628 0.523623 0.341528 0.607885

HYSVGAGITD 3gsn 0.206299 0.223896 0.0685091 0.456901 0.301983 0.839866 0.960392 0.619862 0.743707 0.625628

FYSVGIGQI 3hg1 0.116136 0.40748 0.0267302 0.788768 0.288247 0.684439 0.791986 0.676193 0.51439

IQYYNGEERA 3mv7 0.131303 0.183911 0.137832 0.550189 0.72062 0.71387 0.61675 0.805687 0.678162 0.369976

IHYYNGEERA 3mv8 0.104721 0.319981 0.137301 0.588511 0.711011 0.698685 0.641182 0.780703 0.722655 0.495908

IAYYNGEERA 3mv9 0.110018 0.162188 0.183289 0.571881 0.624239 0.652188 0.580084 0.768668 0.718367 0.523327

HYSNTAGTTG 3qdg 0.190677 0.378438 0.121353 0.907068 0.545921 0.723562 0.864556 0.576701 0.90115 0.445598

HYSYGVKDTD 3qdm 0.192582 0.393812 0.17802 0.788177 0.504967 0.762009 0.981185 0.639363 0.68321 0.72773

HYSVGEGTTA 3sjv 0.151213 0.425377 0.109629 0.668973 0.245775 0.772561 0.662349 0.849326 0.740351 0.523858

FVYSYEKLSI 3vxm 0.112059 0.0564797 0.2566 0.455625 0.676453 0.665721 0.798462 0.689244 0.518651 0.291862

TYFQNEAQLEK 3vxr 0.145454 0.116212 0.0419371 0.437919 0.699024 0.71523 0.50207 0.720446 0.676453 0.504963 0.845973

YYSMNVEVTD 3vxu 0.282704 0.144879 0.00000145 0.564002 0.470094 0.506176 0.849261 0.462855 0.668407 0.424455

HYSVGAGITD 4ftv 0.222661 0.423439 0.0687571 0.586687 0.312305 0.793321 0.882231 0.67633 0.691685 0.699891

YYSASEGTTD 4jrx 0.268508 0.174169 0.00000145 0.233584 0.500914 0.588289 1.03304 0.511454 0.65844 0.551287

FQYYEEEERQ 4jry 0 0.206467 0.12205 0.399856 0.932463 0.752331 0.653574 0.577164 0.639546 0.48289

HYSNTAGTTG 4l3e 0.21054 0.332778 0.195947 0.715054 0.546171 0.666501 0.860983 0.590528 0.81647 0.396452

HYSIHPEYTD 4mnq 0.22149 0.185102 0.00000145 0.677559 0.572473 0.439051 0.904218 0.723073 0.726876 0.706079

IYFQGNSAPDK 5d2l 0.301965 0.385977 0.449897 0.697383 0.924636 1.06507 0.763867 0.656591 0.879783 0.708349 0.850412

TYFNYEAQQDK 5d2n 0 0.294903 0.0504772 0.403806 0.739779 0.753357 0.581906 0.534701 0.768974 0.407517 0.776208

YYSMNVEVTD 5e6i 0.309787 0.150596 0.245985 0.48581 0.783138 0.620001 0.883805 0.634047 0.87082 0.495091

HYSVGVGITD 5e9d 0.18319 0.46124 0.0450157 0.759945 0.311751 0.74823 0.8897 0.613633 0.700427 0.716198

YYSQIVNDFQ 5euo 0.197179 0.135599 0.00000145 0.514361 0.570756 0.569213 0.903068 0.590694 0.627268 0.4911

YYSQIVNDFQ 5isz 0.234344 0.307659 0.190984 0.793318 0.600442 0.685284 0.922021 0.588764 0.666955 0.653308

YYSQIVNDFQ 5jhd 0.269641 0.278016 0.00000145 0.700041 0.662219 0.602303 0.805243 0.398211 0.701869 0.457937

HYSYGVNSTE 5jzi 0.199843 0.315388 0.130623 0.775632 0.394427 0.591821 0.94713 0.446743 0.832166 0.550742

HYSVGAGITD 5men 0.367895 0.296479 0.00000145 0.803353 0.279993 0.778074 0.868959 0.589199 0.435771 0.866236

YYSQIVNDFQ 5nht 0.198627 0.185752 0.00000145 0.568959 0.504444 0.453985 0.672955 0.601421 0.548995 0.626296

YYSQIVNDFQ 5nqk 0.220038 0.301194 0.132595 0.510745 0.604256 0.604557 0.913958 0.578441 0.668625 0.618752

LFYYDKILNR 5sws 0.151572 0.378658 0.271147 0.605016 0.545364 0.876724 0.89875 0.584734 0.697307 0.655902

LFYYDKILNR 5swz 0 0.212632 0.174578 0.53762 0.791478 0.912279 0.815459 0.568035 0.561749 0.585951

YYSMNVEVTD 5tez 0.21524 0.321996 0.00000145 0.642994 0.531109 0.55095 0.863947 0.473515 0.639679 0.463924

IQYYNGEERA 5w1v 0.223703 0.12737 0.175327 0.47555 0.691945 0.578423 0.811625 0.741124 0.694811 0.488251

YYSASEGTTD 5xot 0.267019 0.246515 0.100691 0.561301 0.544699 0.723369 1.10046 0.556433 0.702766 0.719843

FYSVGIGQI 5xov 0.213314 0.43663 0.162096 0.753636 0.184561 0.671061 0.862431 0.658761 0.53167

HYSYGVNSTE 5yxu 0.149029 0.342891 0.06751 0.745733 0.410553 0.689337 0.992584 0.353363 0.734673 0.529849

YFSYDVKMKE 6avf 0.250384 0.150563 0.00000145 0.479276 0.53343 0.547309 0.762415 0.503962 0.756083 0.440387

IQYYNGEERA 6avg 0.118627 0.217695 0.170214 0.495548 0.738113 0.65409 0.807966 0.733622 0.744123 0.462049

FQYYREEENG 6bj2 0.0557233 0.381377 0.329871 0.478466 0.777265 0.43595 0.777593 0.657655 0.471661 0.360866

HYSNTAGTTG 6dkp 0.157658 0.347828 0.194842 0.711493 0.539191 0.727241 0.86817 0.639165 0.755477 0.428024

**Table_nSESA_CDR3a**

CAVTTDSWGKLQF 1ao7 0 0.00000164 0 0.00000121 0.624973 0.659926 0.769122 0.735264 0.185666 0.861737 0.246639 0.464326 0.435339

CAAMEGAQKLVF 1bd2 0 0.00000164 0 0.10094 0.510033 0.516011 0.524941 0.764059 0.559395 0.0737145 0.293359 0.213181

CILPLAGGTSYGKLTF 1mi5 0.090428 0.0535795 0.411857 0.258684 0.509251 0.627798 0.551382 0.905102 0.913248 0.607012 0.405023 0.26467 0.414502 0.18201 0.895759 0.0979854

CAGAGSQGNLIF 1oga 0 0.00000164 0 0.337778 0.196318 0.852244 0.821955 0.220871 0.254177 0.10917 0.336983 0.525784

CALSGFYNTDKLIF 2ak4 0 0.0749197 0.126489 0.252725 0.413653 0.588463 0.818497 0.644069 0.773283 0.480413 0.403692 0.180445 0.396125 0.231266

CAVSHQGRYLTF 2e7l 0 0.00000164 0.0892087 0.26704 0.505849 0.885727 0.905247 0.578223 0.424247 0.11174 0.564878 0.284742

CIVVRSSNTGKLIF 2esv 0.243224 0.0805982 0.443148 0.447385 0.778061 0.796103 0.96453 0.763758 0.26207 0.447347 0.670755 0.137233 0.88378 0.114789

CAVRPTSGGSYIPTF 2f53 0 0.292381 0.183385 0.439208 0.729415 0.817723 1.00717 1.03431 0.969987 0.403536 0.61554 0.511379 0.200123 0.449333 0.38886

CAVSGFASALTF 2oi9 0 0.00000164 0.0944418 0.326443 0.867503 0.906854 0.673953 0.540112 0.38971 0.0889309 0.644971 0.114751

CAVRPLLDGTYIPTF 2p5e 0 0.293848 0.16575 0.403835 0.593109 0.617597 0.894911 0.751286 1.06071 0.478582 0.671913 0.49992 0.289612 0.452385 0.470324

CAVRPLLDGTYIPTF 2p5w 0 0.319026 0.115483 0.338324 0.625544 0.604628 0.92544 0.766905 1.0734 0.463682 0.690231 0.553924 0.250736 0.453284 0.424331

CAVRPLLDGTYIPTF 2pye 0 0.292797 0.178936 0.33809 0.671018 0.61935 0.901289 0.770826 1.0341 0.496521 0.691015 0.538555 0.294002 0.467672 0.425956

CAGAGSQGNLIF 2vlr 0 0.00000164 0 0.374312 0.230818 0.979357 0.851518 0.239164 0.277474 0.118156 0.364545 0.490463

CAVSLERPYLTF 3e2h 0.191282 0.0899515 0.144726 0.484294 0.610696 0.657104 0.666542 0.623095 0.445926 0.137103 0.765327 0.142544

CAVSDPPPLLTF 3e3q 0 0.00000164 0 0.384999 0.35985 0.711226 0.841373 0.889625 0.441628 0.312412 0.467147 0.133921

CAMREDTGNQFYF 3ffc 0 0.00000164 0 0.179164 0.391802 0.768111 0.846552 0.636139 0.671373 0.430878 0.18828 0.466199 0.273705

CARNTGNQFYF 3gsn 0 0.0899482 0.305794 0.356148 0.76006 0.553069 0.844164 0.35525 0.287202 0.555069 0.246196

CAVNVAGKSTF 3hg1 0 0.00000164 0 0.579251 0.37001 0.896784 1.01899 0.646959 0.398377 0.392611 0.216445

CAVQDLGTSGSRLTF 3mv7 0.142689 0.0748234 0.288885 0.263247 0.478057 0.698784 0.889723 0.75259 0.887008 1.03151 0.392388 0.58166 0.0367485 0.625051 0.207406

CAVQDLGTSGSRLTF 3mv8 0 0.00000164 0.172165 0.245922 0.535714 0.681339 0.867887 0.740298 0.927255 1.09998 0.437185 0.841528 0.0794479 0.612234 0.202484

CAVQDLGTSGSRLTF 3mv9 0 0.00000164 0.227871 0.271184 0.294569 0.662878 0.921334 0.683587 0.986632 0.883116 0.394817 0.650393 0.00000094 0.754033 0.181879

CAVNFGGGKLIF 3qdg 0.126413 0.00000164 0 0.251864 0.200131 0.717048 0.539436 0.423277 0.496048 0.335193 0.138769 0.205195

CAGGTGNQFYF 3qdm 0 0.0425712 0.00000212 0.181335 0.717763 0.780533 0.745341 0.432566 0.298796 0.629493 0.393003

CVVRAGKLIF 3sjv 0.143583 0.00000111 0.139581 0.406266 0.512407 1.13021 0.797364 0.113579 0.671627 0.31788

CAVGAPSGAGSYQLTF 3vxm 0.0855484 0.182943 0.346508 0.0559702 0.473652 0.568896 0.300017 1.07669 0.745884 0.709854 0.395725 0.548668 0.287276 0.126817 0.48335 0.284141

CAVRMDSSYKLIF 3vxr 0 0.239056 0.00000111 0.223345 0.38269 0.787104 0.93641 0.720357 0.61694 0.437314 0.178313 0.564853 0.227907

WGTYNQGGKLIF 3vxu 0.234569 0.00000212 0.108815 0.478658 0.583605 0.705783 0.811444 0.420896 0.383378 0.12198 0.503653 0.231234

CAVTTDSWGKLQF 4ftv 0 0.00000164 0.15382 0.173371 0.675471 0.635899 0.693292 0.66643 0.353814 0.743108 0.25165 0.579473 0.385262

CALSGFYNTDKLIF 4jrx 0 0.0841162 0.217839 0.38294 0.392196 0.66722 0.918212 0.608432 0.773328 0.475145 0.648623 0.0888883 0.557589 0.275863

CAVGGGSNYQLIW 4jry 0 0.00000164 0 0.00000212 0.428196 0.963361 0.974129 0.646917 0.516644 0.575329 0.0745262 0.455492 0.201878

CAVNFGGGKLIF 4l3e 0.208064 0.00000164 0.120351 0.327829 0.13539 0.797591 0.689029 0.484244 0.47157 0.187669 0.132412 0.121611

CAVDSATALPYGYIF 4mnq 0 0.00000164 0 0.330414 0.386551 0.848421 0.894465 0.829293 1.00506 0.603907 0.805943 0.370936 0.262683 0.543195 0.241306

CAFITGNQFYF 5d2l 0 0.258243 0.0492814 0.307692 0.396253 1.02775 0.959246 0.659679 0.500957 0.484483 0.630938

CILDNNNDMRF 5d2n 0.245799 0.0978908 0.426158 0.315169 0.810486 0.749076 0.603027 0.651333 0.219785 0.638233 0.267829

CAGPGGSSNTGKLIF 5e6i 0 0.118996 0.245272 0.517361 0.584863 0.501086 1.07565 0.736688 1.05035 0.720189 0.133149 0.448531 0.334722 0.59144 0.276295

CAVTKYSWGKLQF 5e9d 0 0.00000164 0.184945 0.255111 0.617638 0.654342 1.16378 0.782566 0.613546 0.889437 0.512 0.598018 0.19233

CAGAIGPSNTGKLIF 5euo 0 0.00000164 0 0.0814282 0.381993 0.710291 0.822602 0.601104 0.962629 0.759204 0.302084 0.58652 0.147806 0.531987 0.412963

CAFDTNAGKSTF 5isz 0.094569 0.0818033 0.21874 0.141813 0.567866 0.859121 0.881345 0.305454 0.866978 0.659967 0.412617 0.414362

CAWGVNAGGTSYGKLTF 5jhd 0.071997 0.113427 0.326771 0.275673 0.485808 0.477614 0.886143 0.966114 0.59234 1.04213 0.56441 0.428251 0.571911 0.70796 0.238315 0.64167 0.181253

CAYGEDDKIIF 5jzi 0.165404 0.133438 0.240734 0.15543 0.595835 0.750898 0.615805 0.50357 0.0800615 0.56923 0.36976

CAVDSATSGTYKYIF 5men 0 0.00000164 0.0712399 0.411553 0.435796 0.677314 0.810182 0.77287 1.08745 0.748442 0.800103 0.585569 0.302832 0.583752 0.274657

CAVGGGADGLTF 5nht 0 0.00000164 0.24332 0.28805 0.665046 0.995726 0.809158 0.825516 0.235264 0.139115 0.559932 0.163866

CAGGGGADGLTF 5nqk 0.111371 0.0829956 0.661217 0.386672 0.734445 0.771865 0.724956 0.837921 0.00000212 0.17187 0.704562 0.233033

CAASEGSGSWQLIF 5sws 0.09586 0.157274 0.161013 0.204085 0.567258 0.823211 0.982612 0.497146 0.71225 0.469449 0.660232 0.303006 0.385177 0.286346

CAASETSGSWQLIF 5swz 0 0.00000164 0 0.0596609 0.413449 0.865634 0.91866 0.52273 0.344276 0.432924 0.540006 0.127571 0.487729 0.164296

CAASFIIQGAQKLVF 5tez 0 0.00000164 0 0.00000145 0.236375 0.836388 0.510318 0.99557 0.975828 0.565307 0.64048 0.556905 0.352842 0.314521 0.37161

CAGQPLGGSNYKLTF 5w1v 0.0841854 0.00000164 0.221657 0.51646 0.452041 0.804642 1.04884 0.73294 0.85383 0.81888 0.395017 0.629919 0.410713 0.386699 0.363789

CALGEGGAQKLVF 5xot 0.0875019 0.0312706 0.114779 0.00000212 0.417407 0.489623 0.964612 0.626511 0.230439 0.629557 0.179716 0.427915 0.185974

CALGELARSGGYQKVTF 5xov 0.0814794 0.106966 0.193704 0.00000212 0.386611 0.72814 0.464608 1.01255 0.904414 0.683946 0.732329 0.350995 0.369669 0.639029 0.543391 0.642785 0.174744

CAYGEDDKIIF 5yxu 0.0881681 0.0986016 0.308191 0.00000212 0.605727 0.723707 0.418128 0.615383 0.0653901 0.644793 0.200053

CLVGEILDNFNKFYF 6avf 0 0.0243335 0.389386 0.235691 0.617963 0.338272 0.638939 0.726758 0.693955 0.685048 0.494165 0.645231 0.15617 0.983784 0.136462

CLVVDQKLVF 6avg 0.0885901 0.0811383 0.364467 0.324791 0.843193 1.01546 0.969425 0.25242 0.939817 0.176268

CALSHNSGGSNYKLTF 6bj2 0.12419 0.151793 0.178713 0.189309 0.391918 0.7925 0.578479 1.05026 0.703597 0.92185 0.56684 0.910753 0.671701 0.367131 0.478947 0.210868

CAVNFGGGKLIF 6dkp 0.13441 0.0531321 0.00000111 0.3307 0.3101 0.749008 0.511792 0.184054 0.469422 0.160134 0.130719 0.196284

**Table_nSESA_CDR3b**

CASRPGLAGGRPEQYF 1ao7 0 0.00000164 0 0.211883 0.237632 0.356142 0.880603 0.734814 0.607102 1.01469 0.739172 0.424397 0.177777 0.0663411 0.565069 0.28358

CASSYPGGGFYEQYF 1bd2 0 0.00000164 0 0.00000145 0.538219 0.380789 1.07806 0.623991 0.728643 0.619371 0.544413 0.480959 0.0509137 0.683937 0.194554

CASSLGQAYEQYF 1mi5 0 0.00000164 0 0.047782 0.901256 0.262817 0.639185 0.936821 0.779701 0.668706 0.166389 0.638168 0.22465

CASSSRSSYEQYF 1oga 0 0.00000164 0 0.00000145 0.821585 0.764368 0.341418 0.236949 0.475766 0.334703 0.00000094 0.631312 0.31721

CASPGLAGEYEQYF 2ak4 0 0.100243 0.00000145 0.581681 0.554333 0.968807 0.528514 0.546702 0.964271 0.9981 0.712112 0.679277 0.245501 0.137198

CASGGGGTLYF 2e7l 0 0.00000164 0 0.145813 0.992846 0.964778 0.805295 0.283966 0.183751 0.587497 0.170926

CASSQDRDTQYF 2esv 0 0.00000164 0 0.00000145 0.76002 0.705421 0.593291 0.591271 0.381243 0.146224 0.648997 0.218833

CASSYVGNTGELFF 2f53 0 0.00000164 0 0.00000145 0.672382 0.625038 0.511152 0.862385 0.520164 0.28119 0.558478 0.203368 0.518897 0.295404

CASGGGGTLYF 2oi9 0 0.00000164 0 0.0863987 0.966848 1.00313 0.395308 0.380655 0.130737 0.567502 0.206291

CASSYLGNTGELFF 2p5e 0 0.00000164 0 0.00000145 0.782039 0.534233 0.504152 0.895222 0.570149 0.498713 0.589493 0.179448 0.56138 0.296591

CASSYLGNTGELFF 2p5w 0 0.00000164 0 0.00000145 0.673049 0.509436 0.560832 0.889961 0.574823 0.45849 0.536041 0.266107 0.546026 0.301414

CASSYLGNTGELFF 2pye 0 0.00000164 0 0.00000145 0.69944 0.536416 0.552104 0.85593 0.477757 0.371328 0.523185 0.337676 0.61316 0.301167

CASSSRASYEQYF 2vlr 0 0.00000164 0 0.0297241 0.787431 0.759741 0.403333 0.244017 0.48444 0.348503 0.00000094 0.639942 0.289813

CASGGGGTLYF 3e2h 0 0.00000164 0 0.0928847 1.01943 0.508951 0.780139 0.439522 0.119538 0.61662 0.226153

CASGGGGTLYF 3e3q 0 0.00000164 0 0.0419999 0.948394 1.01878 1.2752 0.459136 0.184246 0.510659 0.200249

CASSFTWTSGGATDTQYF 3ffc 0 0.00000164 0 0.00000145 0.769047 0.428906 0.604945 0.837559 0.685873 0.314526 0.348683 0.777394 0.696114 0.108376 0.247834 0.104477 0.610625 0.187901

CASSPVTGGIYGYTF 3gsn 0 0.00000164 0 0.328956 0.559355 0.63123 0.521801 1.09487 0.939778 0.636974 0.833985 0.676563 0.238926 0.600082 0.186578

CAWSETGLGTGELFF 3hg1 0 0.00000164 0.110051 0.301996 0.855395 0.815635 0.810845 1.04197 1.09839 0.618888 0.613251 0.512534 0.274461 0.544234 0.26108

CASSARSGELFF 3mv7 0 0.10661 0.0939502 0.345193 0.420739 1.08226 0.963734 0.400956 0.393437 0.00000094 0.413848 0.221874

CASSARSGELFF 3mv8 0 0.114782 0.0970849 0.343091 0.392503 1.11501 0.931984 0.436206 0.337095 0.00000094 0.393488 0.222074

CASSARSGELFF 3mv9 0 0.00000164 0 0.198868 0.383639 1.05375 0.859083 0.459096 0.341341 0.00000094 0.40133 0.123159

CASSLSFGTEAFF 3qdg 0 0.00000164 0 0.00000145 0.752814 0.592622 0.82384 0.536063 0.601228 0.557864 0.46007 0.580801 0.309562

CAISEVGVGQPQHF 3qdm 0 0.00000164 0.168801 0.155521 0.750385 0.771325 0.762209 0.893647 0.63408 0.615456 0.428665 0.0866146 0.672774 0.259234

CASGQGNFDIQYF 3sjv 0 0.00000164 0 0.192402 0.861888 0.669587 0.628514 0.180655 0.543646 0.407206 0.200885 0.664573 0.194256

CASSPTSGIYEQYF 3vxm 0 0.00000164 0 0.0405677 0.562981 0.740468 1.04867 1.04612 0.783501 0.251462 0.326506 0.0795839 0.491866 0.211604

CASSSWDTGELFF 3vxr 0 0.00000164 0 0.0725709 0.476884 0.897951 0.777726 0.526455 0.392301 0.281047 0.00000094 0.5487 0.297103

CASSGASHEQYF 3vxu 0 0.00000164 0 0.299365 0.565721 0.7408 0.707627 0.632955 0.308565 0.0877318 0.578262 0.276858

CASRPGLMSAQPEQYF 4ftv 0 0.110756 0.0785476 0.236499 0.301517 0.313946 0.800865 0.638059 0.64726 1.04861 0.819072 0.413171 0.327391 0.147924 0.600449 0.147026

CASPGETEAFF 4jrx 0 0.00000164 0 0.391295 0.421612 0.935055 0.652086 0.388457 0.00000164 0.42108 0.288372

CASSRTGSTYEQYF 4jry 0 0.0707496 0.0918087 0.61779 0.773387 0.646498 1.09733 0.809921 0.839133 0.714464 0.680924 0.233423 0.535464 0.210943

CASSWSFGTEAFF 4l3e 0 0.00000164 0 0.00000145 0.788115 0.579494 0.782608 0.652303 0.54065 0.585689 0.143743 0.649815 0.214472

CASSYQGTEAFF 4mnq 0 0.214637 0.0695533 0.285575 0.710799 0.835065 0.784725 0.639363 0.379297 0.311291 0.534206 0.358873

CASSQTQLWETQYF 5d2l 0.310115 0.0922248 0.090127 0.0517419 0.826326 0.734725 0.553192 0.484843 0.565445 0.494177 0.632088 0.19146 0.625525 0.460349

CASSLAPGTTNEKLFF 5d2n 0 0.00000164 0 0.140803 0.696519 0.570815 0.691642 0.507961 0.928981 0.8648 0.703635 0.610674 0.744207 0.254271 0.578334 0.254192

CASSLIYPGELFF 5e6i 0.137242 0.0496615 0.324574 0.207931 0.70124 0.751253 0.542899 0.67816 0.70735 0.82104 0.230867 0.588054 0.18605

CASRPGWMAGGVELYF 5e9d 0 0.00000164 0 0.311866 0.566911 0.507667 0.647456 0.92025 0.93664 0.849645 0.437946 0.94991 0.610974 0.22268 0.579214 0.226553

CASSIRSSYEQYF 5euo 0 0.00000164 0.098489 0.00000145 0.777844 0.74878 0.356027 0.39184 0.412197 0.329675 0.00000094 0.586578 0.257101

CASSIFGQREQYF 5isz 0 0.00000164 0 0.137495 0.818867 0.83829 0.502134 0.588545 0.664603 0.477742 0.196525 0.605623 0.186743

CASSIGVYGYTF 5jhd 0 0.00000164 0 0.00000145 0.692292 0.542349 0.362004 0.702099 0.695884 0.328175 0.430182 0.2136

CASRRGPYEQYF 5jzi 0 0.00000164 0 0.00000072 0.897442 0.281507 0.57999 0.434227 0.379632 0.10084 0.600337 0.394515

CASSYQGTEAFF 5men 0 0.206281 0.068449 0.204592 0.69301 0.829097 0.886277 0.604932 0.446394 0.332695 0.528272 0.387476

CASSQGLAGAGELFF 5nht 0 0.00000164 0 0.00000145 0.633922 0.580733 0.721788 0.0956978 0.00000212 0.639625 0.293713 0.471402 0.0628342 0.425226 0.226079

CASSQGLAGAGELFF 5nqk 0 0.00000164 0 0.00000145 0.722214 0.686252 0.732259 0.148752 0.00000212 0.568168 0.322886 0.553006 0.211772 0.524638 0.274815

CASSAGLDAEQYF 5sws 0 0.00000164 0 0.00000145 0.798129 0.455447 0.520304 0.918484 0.772818 0.770316 0.232384 0.559773 0.316706

CASSRDLGRDTQYF 5swz 0 0.00000164 0 0.00000145 0.590769 0.419809 0.555567 0.203014 0.684623 0.527226 0.476454 0.122968 0.56461 0.193308

CASSLLGGWSEAFF 5tez 0 0.198871 0.089683 0.22434 0.889051 0.646529 0.630994 0.482348 0.682581 0.530934 0.570644 0.450213 0.507699 0.234855

CASSANPGDSSNEKLFF 5w1v 0 0.00000164 0 0.121693 0.109104 0.404207 0.97548 0.89884 0.671713 0.977179 0.960863 0.563934 0.645879 0.594522 0.0419533 0.567604 0.284224

CASRTRGGTLIEQYF 5xot 0 0.00000164 0 0.120794 0.618915 0.606441 0.952729 0.943003 0.731542 0.826634 0.275235 0.221308 0.146961 0.647662 0.212035

CAWSVSVGAGVPTIYF 5xov 0 0.00000164 0.16503 0.00000145 0.57219 0.551826 0.683181 0.975748 0.781283 0.555332 0.761717 0.398725 0.268585 0.120067 0.578775 0.223256

CASRRGSAELYF 5yxu 0 0.00000164 0 0.029921 0.723146 0.695278 0.65399 0.368469 0.140288 0.0826616 0.566064 0.157544

CASSQRQEGDTQYF 6avf 0 0.00000164 0 0.00000145 0.524847 0.783263 1.04345 0.483899 0.148722 0.420222 0.43083 0.100123 0.508402 0.255023

CASSGGHTGSNEQFF 6avg 0 0.00000164 0 0.228119 0.929429 0.551369 0.713198 0.290891 0.568826 1.13068 0.702332 0.51532 0.291667 0.483343 0.181738

CASSFRGGKTQYF 6bj2 0.225579 0.0761734 0.327864 0.495335 0.343305 0.850952 1.05056 0.883932 0.302959 0.364893 0.451376 0.569644 0.16997

CASSWSFGTEAFF 6dkp 0 0.00000164 0.0950756 0.0371629 0.766799 0.545908 0.744568 0.585067 0.610012 0.5065 0.0311189 0.642147 0.210719
