# Supplementary material for: Predicting Antigen‐Specificities of Orphan T Cell Receptors from Cancer Patients with TCRpcDist
Source: Adv Sci (Weinh). 2024 Aug 19;11(40):2405949. doi: 10.1002/advs.202405949 (PMC11516110; doi:10.1002/advs.202405949)
Supplement: Supplementary file 2 — Supporting Information [file ADVS-11-2405949-s001.zip › SI-corrected/DataS2.docx]

Predicting Antigen-Specificities of Orphan T Cell Receptors from Cancer Patients with TCRpcDist

*Marta A. S. Perez^1,2^, Johanna Chiffelle^1,3^, Sara Bobisse^1,3^, Francesca Mayol-Rullan^1,2^, Marine Bugnon^1,2^, Maiia E. Bragina^1,2^, Marion Arnaud^1,3^, Christophe Sauvage ^1,3^, David Barras^1,3^, Denarda Dangaj Laniti^1,3^, Florian Huber^1,3^, Michal Bassani-Sternberg^1,3^, George Coukos^1,3,4^, Alexandre Harari^1,3^ and Vincent Zoete^1,2*^*

^1^ Ludwig Institute for Cancer Research, Lausanne Branch, Department of Oncology, Lausanne University Hospital (CHUV) and University of Lausanne (UNIL), Agora Cancer Research Center, Lausanne, Switzerland.

^2^Molecular Modeling Group, SIB Swiss Institute of Bioinformatics, University of Lausanne, Quartier UNIL-Sorge, Bâtiment Amphipole, CH-1015 Lausanne, Switzerland.

^3^Center for Cell Therapy, CHUV-Ludwig Institute, Lausanne, Switzerland

^4^Immuno-Oncology Service, Department of Oncology, Lausanne University Hospital, Lausanne, Switzerland

*** Correspondence:**Corresponding Author
[Vincent.zoete@unil.ch](mailto:Vincent.zoete@unil.ch)

# Supporting Information

**Data S2 - Reevaluation of TCRpcDist using a larger set of 96 TCRs structures retrieved from Protein Data Bank that recognize 48 distinct known pMHCs.**

In July 2024, we conducted a new benchmark using a set of 96 T-cell receptors (TCRs) that recognize 48 distinct known peptide-major histocompatibility complexes (pMHCs). The Protein Data Bank (PDB) codes utilized in this study were as follows: 1AO7, 1BD2, 1MI5, 1OGA, 2AK4, 2BNQ, 2ESV, 2NX5, 2P5E, 2VLR, 2YPL, 3DXA, 3FFC, 3GSN, 3HG1, 3MV7, 3O4L, 3QDG, 3QDM, 3SJV, 3UTS, 3VXM, 3VXR, 3VXU, 4EUP, 4FTV, 4G8G, 4JFD, 4JRX, 4JRY, 4L3E, 4MJI, 4MNQ, 4QRP, 5BRZ, 5D2L, 5D2N, 5E6I, 5E9D, 5EU6, 5EUO, 5HHO, 5ISZ, 5JHD, 5JZI, 5MEN, 5NHT, 5NME, 5NQK, 5TEZ, 5W1V, 5WKF, 5XOT, 5XOV, 5YXU, 6AVF, 6AVG, 6BJ2, 6MTM, 6R2L, 6RP9, 6RPA, 6RPB, 6RSY, 6TRO, 6UK2, 6ULN, 6ULR, 6UON, 6UZ1, 6VM7, 6VM9, 6VMX, 6VQO, 6VRM, 6ZKW, 7DZM, 7L1D, 7N1E, 7N1F, 7N2N, 7N2P, 7N2S, 7NME, 7OW5, 7PB2, 7PBE, 7PHR, 7QPJ, 7R80, 7RK7, 7RM4, 7RRG, 8CX4, 8GVB, and 8GVI.

We maximized the number of pMHCs by including singleton structures—TCRs that cannot find a pair with the same specificity—to enhance the set size, challenge our approach, and better mimic clinical scenarios where singleton TCRs are common. The weighting parameters (CDR3s contributing 30% each and CDRs 1 and 2 contributing 10% each) and solvent accessibility thresholds (residues with normalized Solvent Exposed Surface Area (nSESA) < 5% in CDRs 1 and 2, and residues with nSESA < 25% in CDR3 excluded from the distance calculation), originally developed using 54 TCRs and 16 pMHCs, remain valid.

Although maximal accuracy was previously observed for TCRpcDist-3D in the 54-structure set ( refer to **Figure 2 of the main manuscript**), in this larger set of 96 structures, TCRpcDist6CDRs and TCRpcDist-3D demonstrated similar accuracy, each presenting an AUC of 0.83 ± 0.1 (see **figure below**). The inclusion of 3D structures added value for several specificities, though it proved less efficient for others (see **table below**). We do not exclude the possibility of further improving TCRpcDist as more TCR-pMHC crystal structures become available.

**Figure.** TCRpcDist clustering TCRs and correlating with their specificity for a set of 96 TCRs recognizing 48 different pMHC. The clustering efficiency was measured by the number of color changes, the pMHC-distance and the AUC, similarly to the analysis performed in the main manuscript for the developmental set.

A) shows hierarchical clustering using the Atchley-based distance considering only sliding windows of 4 consecutive residues of the CDR3β. After clustering, each TCR is colored according to the pMHC it binds. The sequence of the bound peptide is also given.

B) shows the hierarchical clustering using the Atchley-based distance considering all 6 TCR CDRs (i.e., CDR1α, CDR2α, CDR3α, CDR1β, CDR2β, and CDR3β), with CDR3s contributing 30% each and CDRs 1 and 2 10% each. After clustering, each TCR is colored according to the pMHC it binds. The sequence of the bound peptide is also given.

C) shows hierarchical clustering using the Atchley-based distance considering all 6 TCR CDRs and normalized solvent acessibility thresholds (nSESA). CDR3s contributing 30% each and CDRs 1 and 2 10% each to the final distance. Residues with nSESA < 5% in CDRs 1 and 2 and residues with nSESA < 25% in CDRs 3 were excluded from the distance calculation.

**Table**. Average branch length distance, pMHC-distance, for non-singleton peptides in the Evolutionary Tree for the set of 96 TCRs considering three different scenarios: just CDR3beta, all 6 CDRs and all CDRs + nSESA based. The number of TCRs per peptide is also presented, in column1. Each peptide contributes with the same weight (1/48) to the final pMHC-distance, to prevent this measure to be biased by the most frequent pMHCs. Singleton peptides cannot be paired and contribute with 0.

| **nTCRs** | **peptide** | **pMHC-distance** | | |
| --- | --- | --- | --- | --- |
|  |  | **TCRpcDist-CDR3b** | **TCRpcDist-6CDRs** | **TCRpcDist-3D** |
| 2 | ALHGGWTTK | 0.94 | 0.91 | 0.55 |
| 2 | APRGPHGGAASGL | 1.06 | 0.77 | 0.39 |
| 3 | FLRGRAYGL | 1.12 | 0.78 | 0.74 |
| 3 | GADGVGKSXX | 0.71 | 0.43 | 0.52 |
| 8 | GILXXVFTL | 0.89 | 0.54 | 0.65 |
| 3 | HMTEVVRHC | 1.07 | 0.73 | 0.61 |
| 2 | ILAKFLHWL | 0.88 | 0.09 | 0.06 |
| 2 | IPLTEEAEL | 1.40 | 0.92 | 0.92 |
| 2 | IXDQVPFSV | 0.67 | 0.54 | 0.89 |
| 2 | KLVALGINAV | 0.00 | 0.13 | 0.18 |
| 4 | LLFGXXVYV | 1.01 | 0.43 | 0.41 |
| 3 | NLVPMVATV | 0.50 | 0.71 | 0.86 |
| 5 | RXPLTFGWXX | 0.81 | 0.71 | 0.76 |
| 5 | SLLMWITQX | 0.99 | 0.71 | 0.74 |
| 2 | TRLALIAPK | 0.37 | 0.08 | 0.08 |
| 2 | VMAPRTLIL | 1.15 | 0.64 | 0.59 |
| 2 | VVVGAXGVGK | 0.00 | 0.00 | 0.06 |
| 4 | XXEPLPQGQLTAY | 0.78 | 0.73 | 0.69 |
| 9 | XXXXIGILTV | 0.86 | 0.44 | 0.57 |
| 2 | YLEPGPVTV | 1.13 | 0.67 | 0.95 |
| 2 | YLQPRTFLL | 0.91 | 0.30 | 0.34 |

Note: Among the 48 specificities studied, 21 peptides had more than one TCR recognizing them, and these are listed in the table. In contrast, 28 peptides were recognized by only a single TCR, contributing a value of 0 to the final pMHC-distance. An "X" in the peptide sequence indicates that a given position might be mutated across the PDB structures used. These peptides were clustered together due to their sequence similarity. For example, GADGVGKSXX can represent GADGVGKSL, GADGVGKSAL, or GADGVGKSL.
