# Supplementary material for: Predicting Antigen‐Specificities of Orphan T Cell Receptors from Cancer Patients with TCRpcDist
Source: Adv Sci (Weinh). 2024 Aug 19;11(40):2405949. doi: 10.1002/advs.202405949 (PMC11516110; doi:10.1002/advs.202405949)
Supplement: Supplementary file 2 — Supporting Information [file ADVS-11-2405949-s001.zip › SI-corrected/DataS4.docx]

Predicting Antigen-Specificities of Orphan T Cell Receptors from Cancer Patients with TCRpcDist

*Marta A. S. Perez^1,2^, Johanna Chiffelle^1,3^, Sara Bobisse^1,3^, Francesca Mayol-Rullan^1,2^, Marine Bugnon^1,2^, Maiia E. Bragina^1,2^, Marion Arnaud^1,3^, Christophe Sauvage ^1,3^, David Barras^1,3^, Denarda Dangaj Laniti^1,3^, Florian Huber^1,3^, Michal Bassani-Sternberg^1,3^, George Coukos^1,3,4^, Alexandre Harari^1,3^ and Vincent Zoete^1,2*^*

^1^ Ludwig Institute for Cancer Research, Lausanne Branch, Department of Oncology, Lausanne University Hospital (CHUV) and University of Lausanne (UNIL), Agora Cancer Research Center, Lausanne, Switzerland.

^2^Molecular Modeling Group, SIB Swiss Institute of Bioinformatics, University of Lausanne, Quartier UNIL-Sorge, Bâtiment Amphipole, CH-1015 Lausanne, Switzerland.

^3^Center for Cell Therapy, CHUV-Ludwig Institute, Lausanne, Switzerland

^4^Immuno-Oncology Service, Department of Oncology, Lausanne University Hospital, Lausanne, Switzerland

*** Correspondence:**Corresponding Author
[Vincent.zoete@unil.ch](mailto:Vincent.zoete@unil.ch)

# Supporting Information

**Data S4 – Further details on the performance comparison between TCRpcDist and the following approaches: TCRbase, TCRdist3, TCRbase 1.0, NetTCR-2.2 and SwarmTCR.**

**Comparison of the specificities predicted by TCRpcDist-3D, NetTCR-2.2, TCRbase, and TCRdist3 for a subset of TCRs from the private set**

Table – Comparison of the specificities predicted by TCRpcDist-3D, NetTCR-2.2, TCRbase, and TCRdist3 for a subset of TCRs from the private set. The subset includes 12 TCRs known to bind ELAGIGILTV and 5 TCRs known to bind RAKFKQLL. The predicted specificities for rank 1 and rank 2 are displayed, with correct predictions highlighted in green.

**Comparison with SwarmTCR**

A comparison with the predictive model SwarmTCR (PMID: 34493215), is presented below, further illustrating the competitiveness of TCRpcDist. SwarmTCR is a supervised predictive model that requires training on a specific set of TCRs before it can perform clustering tasks. Due to that fact, direct comparison between TCRpcDist and SwarmTCR for the sets used in the current manuscript was not possible. To circumvent this issue , we applied TCRpcDist on a set of TCRs on which SwarmTCR was trained, knowing that the results could be favoring Swarm TCR. We compared SwarmTCR, TCRdist3, and TCRpcDist using the same datasets employed in the SwarmTCR study (PMID: 34493215) for the peptides YVLDHLIVV, GILGFVFTL, and NLVPMVATV. The results are shown in the table below. As illustrated, despite SwarmTCR's training advantage, TCRpcDist and TCRdist3 perform comparably well. However, due to the aforementioned reasons, we believe these results do not accurately represent the true predictive capabilitiTCes of TCRpcDist-3D. Therefore, we have decided not to include this comparison in our manuscript.


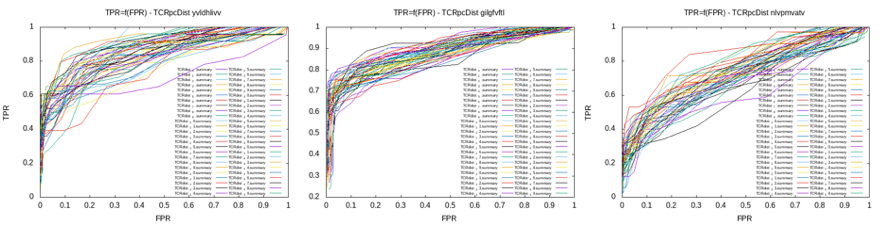


Figure -True Positive Rate as a function of the False Positive Rate for three peptides using TCRpcDist. For comparison purposes we use 50 iterations of the Reference and Test sets. Sets are provided in Swarm paper PMID: 34493215.

Table – Average Area Under ROC curves and Standard Deviations for the 3 peptides and 50 iterations described upwards. We compare SwarmTCR (supervised predictive model), TCRdist3 (unsupervised predictive model) and TCRpcDist-3D.

|  | SwarmTCR AUROC | TCRdist AUROC | TCRpcDist AUROC |
| --- | --- | --- | --- |
| YVLDHLIVV | 0.86 +/- 0.05 | 0.84 +/- 0.05 | 0.83 +/- 0.04 |
| GILGFVFTL | 0.88+/- 0.02 | 0.88 +/- 0.02 | 0.87 +/- 0.02 |
| NLVPMVATV | 0.69 +/- 0.06 | 0.68 +/- 0.05 | 0.71 +/- 0.04 |
