# Supplementary material for: Predicting Antigen‐Specificities of Orphan T Cell Receptors from Cancer Patients with TCRpcDist
Source: Adv Sci (Weinh). 2024 Aug 19;11(40):2405949. doi: 10.1002/advs.202405949 (PMC11516110; doi:10.1002/advs.202405949)
Supplement: Supplementary file 2 — Supporting Information [file ADVS-11-2405949-s001.zip › SI-corrected/DataS5.pdf]

## Supporting Information

### Benchmarking TCRpcDist using a set of 48 structures and comparison with the developmental set of 54 structures used in the main manuscript.

Since our approach considers aspects of TCR-pMHC 3D interactions, we have started working worked with a dataset of TCRs for which the TCR-pMHC 3D structures were known. Due to this, a set of 54 TCRs was the largest set available for developing our approach as of June 2020. This set included structures with single point mutations (SP). As the SP mutations are in CDRs1, 2 and CDR3, we kept them for the following reasons:

1. To increase the size of the set,
2. To check the effect of SP on the predictive ability, as they are placed in regions that contact the pMHC,
   It is known that SP mutations may result in different TCR specificity.

We excluded TCRs with SP mutations from the 54-set and ended up wit a training set of 48 TCRs. The PDB identifiers excluded from the 54 set in **SI Table 1** were 2P5W, 2PYE, 2VLR, 3MV8, 3MV9 and 5NQK. We benchmarked our approach using the 48-set and reached the same conclusions regarding the best combination of parameters to use in our approach (tables and discussion below).

The table below shows the quality of the cluster as measured by the number of color changes and the pMHC-distance, for diverse weightings of the contributions of the various CDRs. The maximal clustering efficiency is highlighted in yellow and obtained when each CDR3s contribute by 30% and each of the remaining CDRs by 10% to the distance calculation. The same conclusion holds for the 54 and 48 set.

| | | | | | | **54 set** | | **48 set** | |
|---|---|---|---|---|---|---|---|---|---|
| **CDR1a** | **CDR1b** | **CDR2a** | **CDR2b** | **CDR3a** | **CDR3b** | **color-change** | **pMHC-distance** | **color-change** | **pMHC-distance** |
| 0% | 0% | 0% | 0% | 0% | 100% | **39** | **0.52** | **38** | **0.54** |
| 0% | 0% | 0% | 0% | 40% | 60% | 35 | 0.46 | **35** | **0.45** |
| 0% | 0% | 0% | 0% | 50% | 50% | 33 | 0.46 | **33** | **0.44** |
| 0% | 0% | 0% | 0% | 60% | 40% | 38 | 0.49 | **38** | **0.53** |
| 0% | 0% | 0% | 0% | 100% | 0% | 40 | 0.60 | **40** | **0.6** |
| 5% | 5% | 5% | 5% | 40% | 40% | 34 | 0.43 | **34** | **0.42** |
| 10% | 10% | 10% | 10% | 30% | 30% | 31 | 0.44 | **32** | **0.43** |

The results exploring the best nSESA threshold are below. Similarly, to the 54 set, for the 48-set the clustering efficiency as measured by the number of color changes and the pMHC-distance is maximal when residues with nSESA < 5% in CDRs 1 and 2 and residues with nSESA < 20% in CDRs 3 are excluded from the distance calculation.

| | | 54 set | | 48 set | |
|---|---|---|---|---|---|
| | **% SESA CDR3(CDR1and2)** | **color change** | **pMHC-distance** | **color change** | **pMHC-distance** |
| **% SESA CDR3(CDR1and2)** | **5%(5%)** | 31 | 0.42 | **33** | **0.43** |
| | **10%(10%)** | 32 | 0.41 | **32** | **0.42** |
| | **15%(15%)** | 32 | 0.50 | **31** | **0.43** |
| | **20%(20%)** | 32 | 0.44 | **30** | **0.44** |
| | **25%(25%)** | 35 | 0.42 | **32** | **0.43** |
| | **30%(30%)** | 31 | 0.47 | **32** | **0.45** |
| | **35%(35%)** | 31 | 0.43 | **33** | **0.41** |
| | **40%(40%)** | 36 | ND | **ND** | **ND** |
| | **20%(5%)** | 29 | **0.39** | **30** | **0.40** |
| | **25%(5%)** | 34 | 0.41 | **31** | **0.42** |
| | **30%(5%)** | 32 | 0.42 | **30** | **0.41** |

TCRpcDist is employed for TCR repertoire analysis and specificity prediction. To assess the approach's effectiveness in grouping TCRs with the same specificity, we selected two non-standard performance metrics: color change and pMHC-distance. These metrics demonstrate the capability of our method to cluster TCRs with identical specificities based on a hierarchical clustering tree. Additionally, we provided a more conventional evaluation metric using ROC curves.

We have benchmarked our approach using the silhouette score as well. We plotted the silhouette score as a function of the number of clusters (K) and compared these plots across different parameter combinations (various CDR weights and different SESA thresholds, covering all parameter combinations benchmarked in the manuscript). The silhouette score and k-means clustering were computed with Python 3.8.13 using sklearn.cluster and sklearn.metrics. The plots depicting the silhouette score as a function of the number of clusters can be seen in the figure below. The combinations of SESA thresholds and CDR weights are noted in the upper right corner of each corresponding graph. For example, in the legend "CDR3a = 30%, CDR3b = 30%, CDR1a, 1b, 2a, 2b = 10%", means that the weight contribution of the CDRs to the distance calculation were 30% for CDR3 alpha, 30% for CDR3 beta, and 10% for CDRs 1 and 2 alpha and beta. For example, for the SESA nomenclature in the legend "SESA CDR3(CDR1 and 2) = 20%(5%)" indicates that CDR3 alpha and CDR3 beta

residues with SESA lower than 20% were excluded from the distance calculation and simulataneously CDRs 1 and 2 alpha and beta residues with SESA lower than 5% were excluded from the distance calculation.

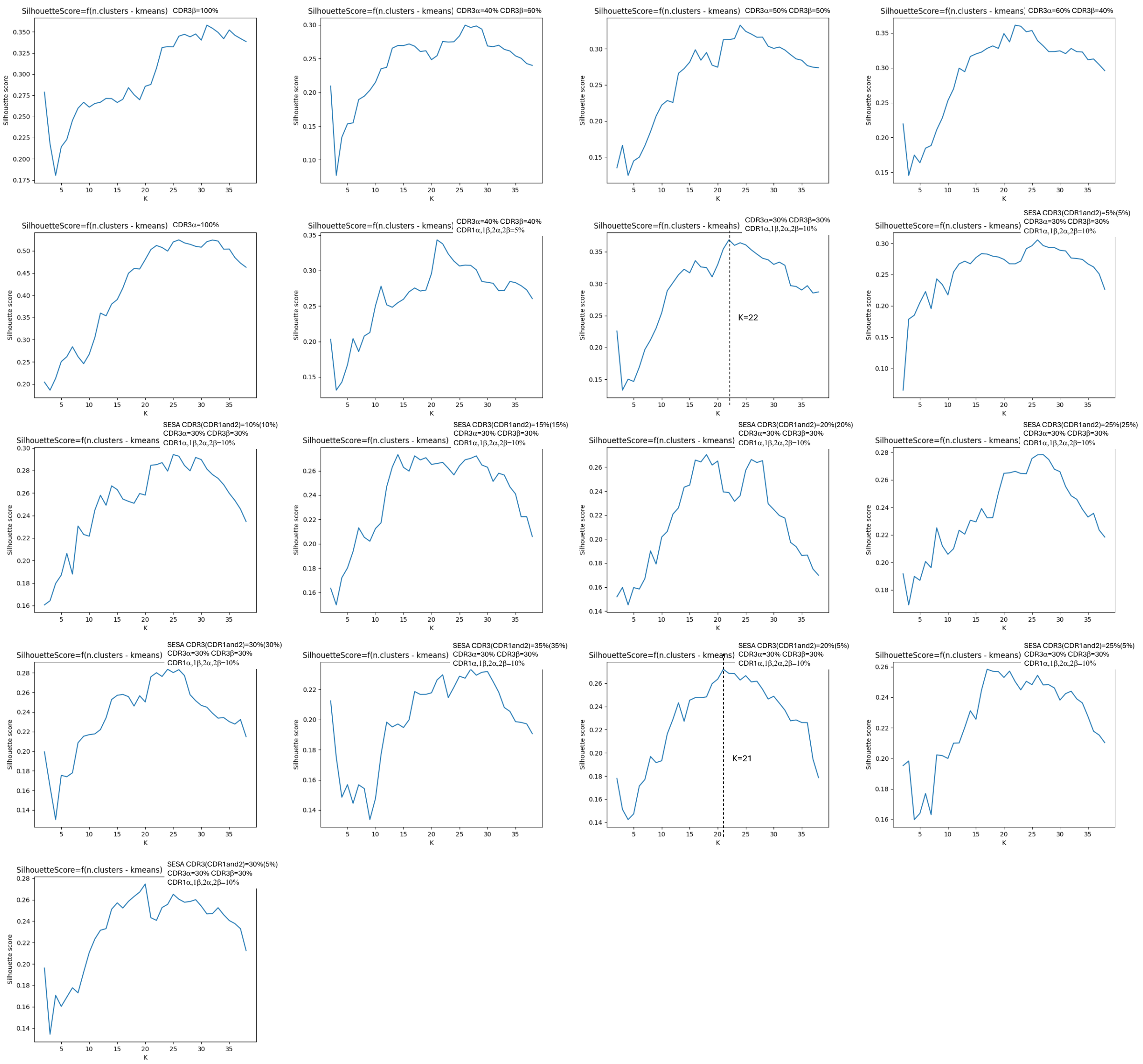


Figure. Interpreting clustering quality using silhouettte score for different number of clusters, K, while using different combinations of CDR weights and SESA thresholds. Data set of 54 TCRs with known 3D structure and specificity. The combination of the SESA parameters and CDRs weights are written on the right upper corner of each corresponding graph. For example, in the legend “CDR3a = 30%, CDR3b = 30%, CDR1a, 1b, 2a, 2b = 10%”, means that the weight contribution of the CDRs to the distance calculation was 30% for CDR3 alpha, 30% for CDR3 beta, and 10% for CDRs 1 and 2 alpha and beta. For example, regarding the SESA nomenclature in the legend “SESA CDR3(CDR1 and 2) = 20%(5%)” indicates that CDR3 alpha and CDR3 beta residues with SESA lower than 20% were excluded from the distance calculation and simulataneously CDRs 1 and 2 alpha and beta residues with SESA lower than 5% were excluded from the distance calculation.

We selected the optimal number of clusters (K) for each condition based on the maximization of the silhouette score. The highest silhouette scores, which indicate that the TCRs are well-matched to their own clusters and poorly matched to neighboring clusters, were observed when each TCR CDR3 alpha contributed 100% to the distance calculation. For K > 20, the silhouette scores were higher than 0.5. While the silhouette score effectively evaluates how well the clusters are separated, it does not necessarily reflect the quality of clustering in terms of grouping TCRs with the same specificity.

Therefore, we computed the purity of the clusters for the different conditions using the optimal K. Purity is a measure of how well each cluster represents a single specificity. It is calculated by first identifying the most common specificity in each cluster (major class), second counting the number of TCRs in each cluster belonging to the major class, third summing these counts across all clusters and finally dividing by the total number of TCRs in the dataset.

Our analysis revealed that for the 54 set, without incorporating SESA thresholds, the highest purity (0.63) is obtained when each CDR3 contributes 30% and each of the remaining CDRs contributes 10% to the distance calculation for a K=22. Additionally, the clustering purity is maximized (0.65) when residues with nSESA < 5% in CDRs 1 and 2 and residues with nSESA < 20% in CDRs 3 are excluded from the distance calculation for a K=21.

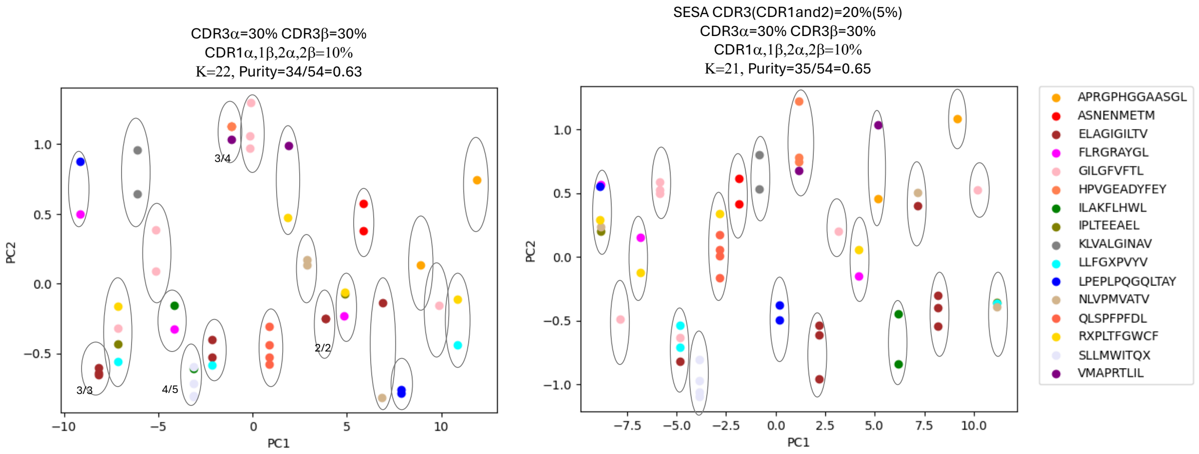


Figure. Principal Component Analysis (PCA) plot showing the clustering algorithm results for the 54 set and the best K. The K clusters are identified in black circles and each one of the 54 TCRs is color coded according to its specificity. On the left: without incorporating SESA thresholds, the purity is 0.63 and is obtained when each CDR3 contributes 30% and each of the remaining CDRs contributes 10% to the distance calculation for a K=22. On the right: when residues with nSESA < 5% in CDRs 1 and 2 and residues with nSESA < 20% in CDRs 3 are excluded from the distance calculation, the purity is 0.65 for a K=21.

The same conclusion held for the 48 set with proper redundancy reduction. Our analysis revealed that for the 48 set, without incorporating SESA thresholds, the highest purity (0.63) is obtained when each CDR3 contributes 30% and each of the remaining CDRs contributes 10% to the distance calculation for a K=22. Additionally, the clustering purity is maximized (0.65) when residues with nSESA < 5% in CDRs 1 and 2 and residues with nSESA < 20% in CDRs 3 are excluded from the distance calculation for a K=21.

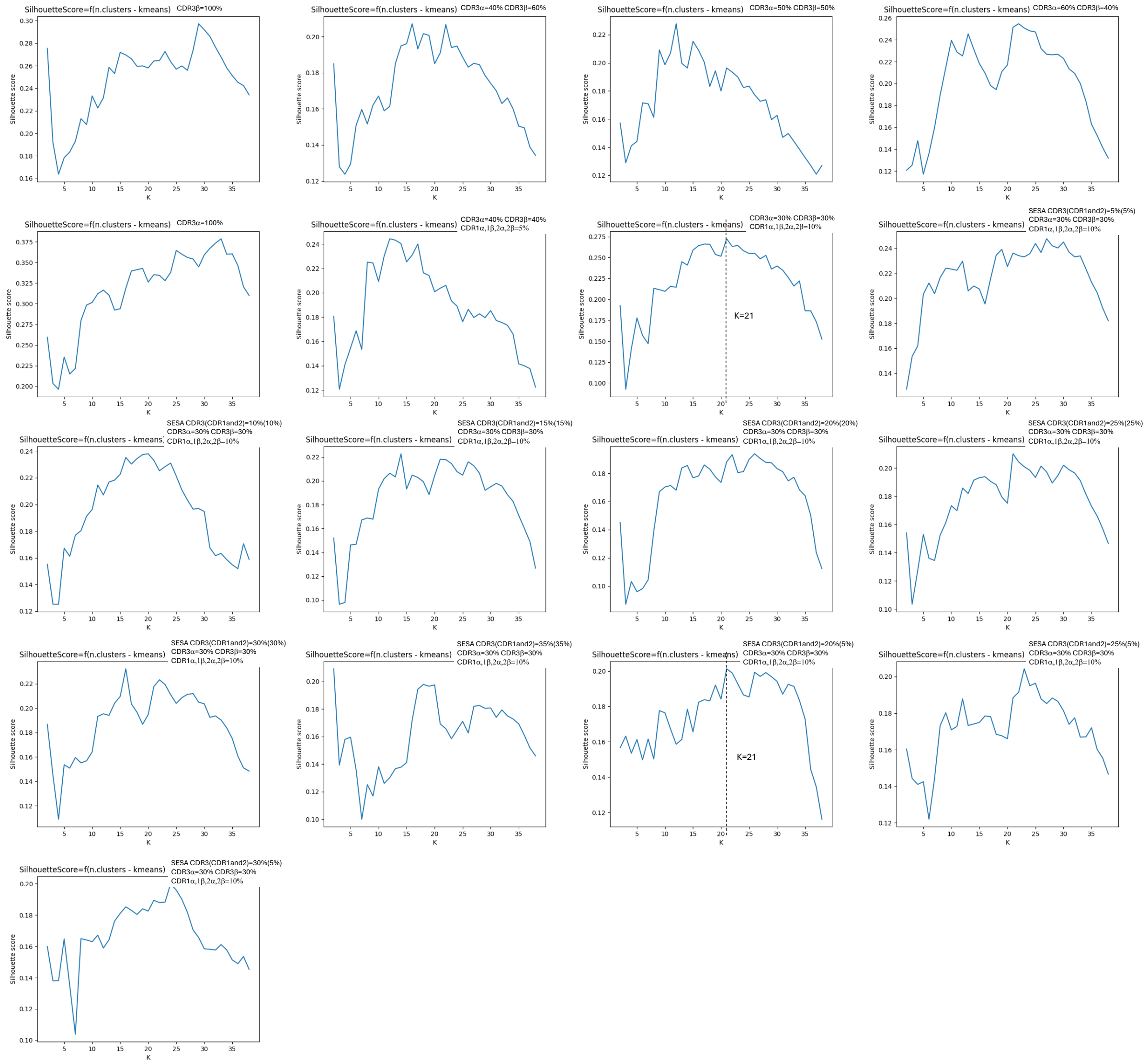


Figure. Interpreting clustering quality using silhouettte score for different number of clusters, K, while using different combinations of CDR weights and SESA thresholds. Data set of 48 TCRs with known 3D structure and specificity. The combination of the SESA parameters and CDRs weights are written on the right upper corner of each corresponding graph. For example, in the legend "CDR3a = 30%, CDR3b = 30%, CDR1a, 1b, 2a, 2b = 10%", means that the weight contribution of the CDRs to the distance calculation was 30% for CDR3 alpha, 30% for CDR3 beta, and 10% for CDRs 1 and 2 alpha and beta. For example, regarding the SESA nomenclature in the legend "SESA CDR3(CDR1 and 2) = 20%(5%)" indicates that CDR3 alpha and CDR3 beta residues with SESA lower than 20% were excluded from the distance calculation and simulataneously CDRs 1 and 2 alpha and beta residues with SESA lower than 5% were excluded from the distance calculation.

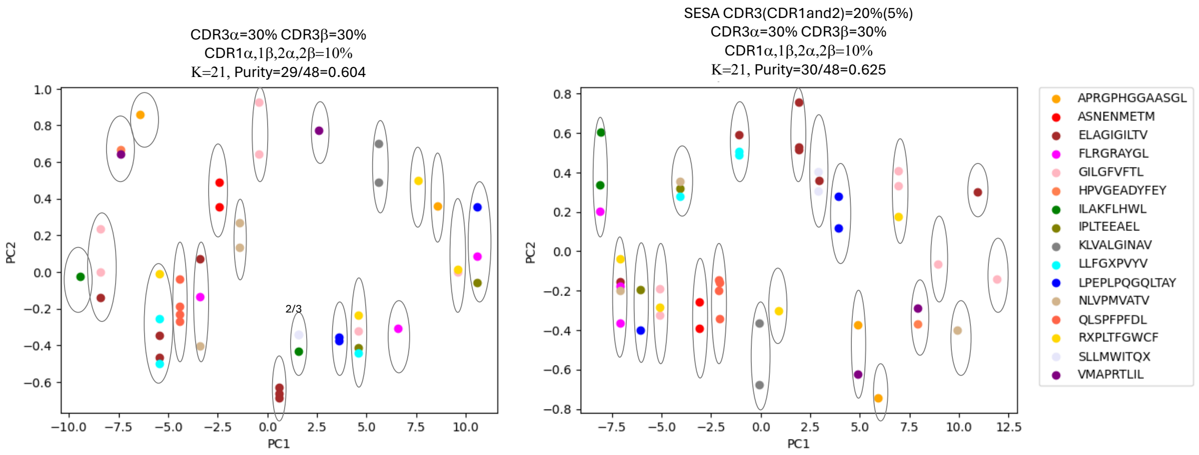


Figure. Principal Component Analysis (PCA) plot showing the clustering algorithm results for the 48 set and the best K. The K clusters are identified in black circles and each one of the 48 TCRs is color coded according to its specificity. On the left: without incorporating SESA thresholds, the purity is 0.60 and is obtained when each CDR3 contributes 30% and each of the remaining CDRs contributes 10% to the distance calculation for a K=21. On the right: when residues with nSESA < 5% in CDRs 1 and 2 and residues with nSESA < 20% in CDRs 3 are excluded from the distance calculation, the purity is 0.63 for a K=21.
