# Supplementary material for: Predicting Antigen‐Specificities of Orphan T Cell Receptors from Cancer Patients with TCRpcDist
Source: Adv Sci (Weinh). 2024 Aug 19;11(40):2405949. doi: 10.1002/advs.202405949 (PMC11516110; doi:10.1002/advs.202405949)
Supplement: Supplementary file 2 — Supporting Information [file ADVS-11-2405949-s001.zip › SI-corrected/DataS6.pdf]

Clustering VDJ[2019] TCR complexes 374 unique TCRs considered

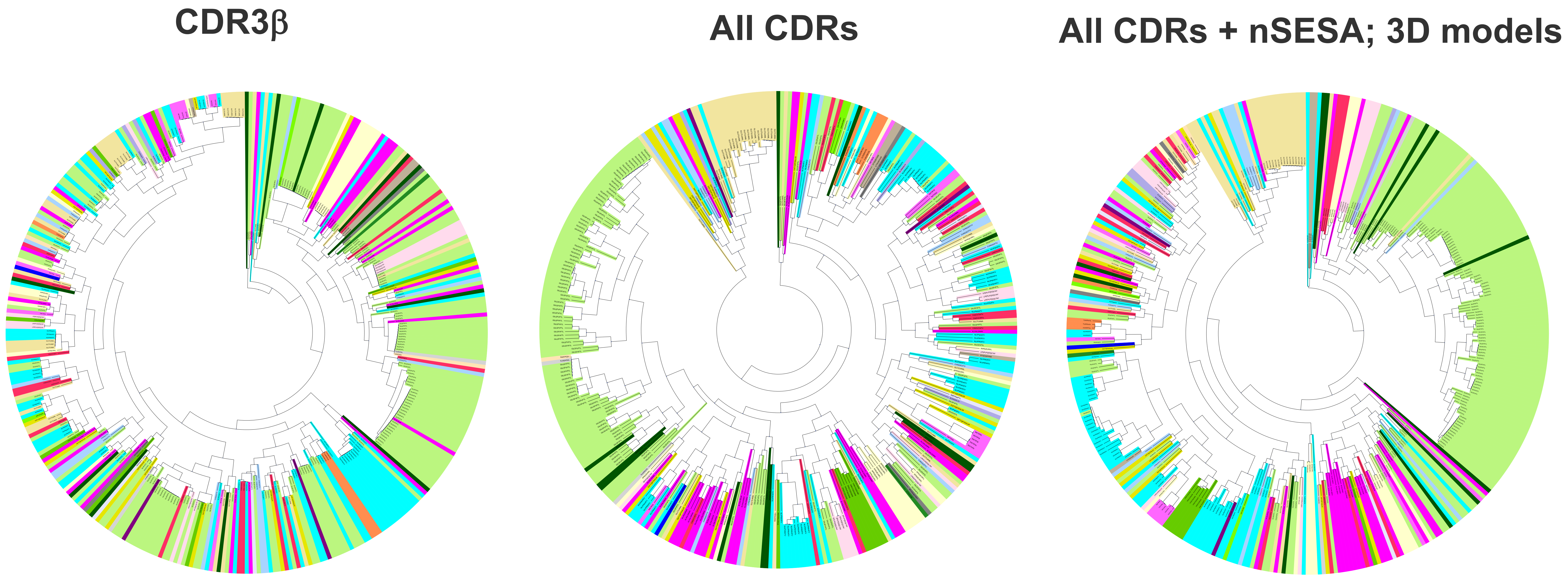

Color change = 198
pMHC-distance =0.87

Color change = 157
pMHC-distance =0.57

Color change = 154
pMHC-distance =0.55

Quality of the cluster weighting the contribution of different CDRs

CDR1a	CDR1b	CDR2a	CDR2b	CDR3a	CDR3b	color-change
0%	0%	0%	0%	0%	100%	**198**
0%	0%	0%	0%	40%	60%	184
0%	0%	0%	0%	50%	50%	189
0%	0%	0%	0%	60%	40%	192
0%	0%	0%	0%	100%	0%	182
5%	5%	5%	5%	40%	40%	168
10%	10%	10%	10%	30%	30%	157

Exploring the best SESA threshold

% SESA CDR3(CDR1and2)	color change
**5%(5%)**	162
**10%(10%)**	167
**15%(15%)**	166
**20%(20%)**	165
**25%(25%)**	174
**30%(30%)**	166
**35%(35%)**	187
**40%(40%)**	193
**20%(5%)**	154
**25%(5%)**	158
**30%(5%)**	158
