# Supplementary material for: Predicting Antigen‐Specificities of Orphan T Cell Receptors from Cancer Patients with TCRpcDist
Source: Adv Sci (Weinh). 2024 Aug 19;11(40):2405949. doi: 10.1002/advs.202405949 (PMC11516110; doi:10.1002/advs.202405949)
Supplement: Supplementary file 2 — Supporting Information [file ADVS-11-2405949-s001.zip › SI-corrected/SI-webservice/input-TCRpcDist-webservice.pdf]

1 TRAV13-1 CAASDSSASKIIFG TRAJ3 TRBV9 CASSVGKETQYFG TRBJ2-5 FAFGEPREL
2 TRAV14/DV4 CAMREPYYNQGGKLIFG TRAJ23 TRBV4-3 CASSQDRLARDTQYFG TRBJ2-3 FAFGEPREL
3 TRAV26-2 CILRAVYVRFG TRAJ43 TRBV5-5 CASSWKGVYNQPQHFG TRBJ1-5 RRWDEKAVDKSK
4 TRAV20 CAVQATSGSARQLTFG TRAJ22 TRBV27 CASSLSKTGRYNEQFFG TRBJ2-1 GTDSDSSRQK
5 TRAV14/DV4 CAMRAGGYNKLIFG TRAJ4 TRBV14 CASSHWTSGSGETQYFG TRBJ2-5 ILRGSVAHK
6 TRAV38-2/DV8 CARGTGNQFYFG TRAJ49 TRBV10-3 CAISAQGREQYFG TRBJ2-7 RVRAYTYSK
7 TRAV17 CATVFYGNNRLAFG TRAJ7 TRBV5-5 CASSFFPDSNQPQHFG TRBJ1-5 GLYDGMEHL
8 TRAV22 CAVGVLRDYKLSFG TRAJ20 TRBV2 CASIQGTGLAYTFG TRBJ1-2 GLYDGMEHL
9 TRAV21 CAVAVFPGNQFYFG TRAJ49 TRBV2 CASNLGQAIKYTFG TRBJ1-2 GLYDGMEHL
10 TRAV38-2/DV8 CAYRSAMYSGGGADGLTFG TRAJ45 TRBV7-8 CASSLGGSFQPQHFG TRBJ1-5 GLYDGMEHL
11 TRAV17 CATDAYNFNKFYFG TRAJ21 TRBV2 CASRANTGELFFG TRBJ2-2 GLYDGMEHL
12 TRAV19 CALSERPGGATNKLIFG TRAJ32 TRBV2 CASIVGQGNEQFFG TRBJ2-1 GLYDGMEHL
13 TRAV12-2 CAVKVQGAQKLVFG TRAJ54 TRBV9 CASSLTGYGYTFG TRBJ1-2 GLYDGMEHL
14 TRAV12-2 CAVKDGNTGKLIFG TRAJ37 TRBV9 CASSLTGYGYTFG TRBJ1-2 GLYDGMEHL
15 TRAV17 CATGISEENTGNQFYFG TRAJ49 TRBV6-1 CASSPLRDYFNEQFFG TRBJ2-1 GLYDGMEHL
16 TRAV12-2 CAVKGSGTSYGKLTFG TRAJ52 TRBV9 CASSLTGYEQFFG TRBJ2-1 GLYDGMEHL
17 TRAV12-2 CAVRGQAGTALIFG TRAJ15 TRBV9 CASSLTGYEQFFG TRBJ2-1 GLYDGMEHL
18 TRAV22 CATMEYGNKLVFG TRAJ47 TRBV5-5 CASSLENTEAFFG TRBJ1-1 GLYDGMEHL
19 TRAV22 CATMEYGNKLVFG TRAJ47 TRBV5-6 CASSPQGNSNQPQHFG TRBJ1-5 GLYDGMEHL
20 TRAV12-2 CAVNSGGGADGLTFG TRAJ45 TRBV20-1 CSATEGTPQFFG TRBJ2-1 ELAGIGILTV
21 TRAV27 CAGEAFGGNYQLIWGAG TRAJ33 TRBV4-3 CASSPDLAGVNEQFFG TRBJ2-1 ELAGIGILTV
22 TRAV35 CAGPNAGGTSYGKLTFG TRAJ52 TRBV4-2 CASSQDLAIGEQYFG TRBJ2-7 ELAGIGILTV
23 TRAV19 CALSEARGGADGLTFG TRAJ45 TRBV6-5 CASSYSGTSGIYEQYFG TRBJ2-7 ELAGIGILTV
24 TRAV12-1 CVVNGGYNNNDMRFG TRAJ43 TRBV6-5 CASSYSLSGTSSYEQYFG TRBJ2-7 ELAGIGILTV
25 TRAV12-2 CAVNAGNQFYFG TRAJ49 TRBV4-1 CASSPDRSADTQYFG TRBJ2-3 ELAGIGILTV
26 TRAV12-2 CAVGDYKLSFG TRAJ20 TRBV29-1 CSASRDIDSGNTIYFG TRBJ1-3 ELAGIGILTV
27 TRAV26-1 CIVRVPSDNFNKFYFG TRAJ21 TRBV28 CASSRTFRELFFG TRBJ2-2 ELAGIGILTV
28 TRAV22 CAVGLYNNNDMRFG TRAJ43 TRBV27 CASSFALGGGSSYNEQFFG TRBJ2-1 ELAGIGILTV
29 TRAV26-2 CILRDGTDKLIFG TRAJ34 TRBV27 CASSLWGTSGQIVTQYFG TRBJ2-5 ELAGIGILTV
30 TRAV3 CAVRDVNFNKFYFG TRAJ21 TRBV4-1 CASSQDFSSGNEQFFG TRBJ2-1 ELAGIGILTV
31 TRAV10 CVVSPLSGTYKYIFG TRAJ40 TRBV4-1 CASSQDFSSGNEQFFG TRBJ2-1 ELAGIGILTV
32 TRAV20 CAVQAPYSGAGSYQLTFG TRAJ28 TRBV5-1 CASKFGDTQYFG TRBJ2-3 TADFDITEL
33 TRAV1-2 CAVRTGYSGAGSYQLTFG TRAJ28 TRBV5-1 CASSYGNEQFFG TRBJ2-1 TADFDITEL
34 TRAV1-2 CAVIRGYSGAGSYQLTFG TRAJ28 TRBV5-1 CASKFGNELFFG TRBJ2-1 TADFDITEL
35 TRAV12-2 CAAFFRNDYKLSFG TRAJ20 TRBV7-9 CASRRTGGIGDGYTFG TRBJ1-2 TADFDITEL
36 TRAV13-1 CASYSGNTGKLIFG TRAJ37 TRBV9 CASSVTSGTLYFG TRBJ2-5 TADFDITEL
37 TRAV26-1 CIVRVPSGAGSYQLTFG TRAJ28 TRBV9 CASSVVGTREQFFG TRBJ2-1 TADFDITEL
38 TRAV12-2 CAVTPTSTLTFG TRAJ11 TRBV5-1 CASSWEGDGTDTQYFG TRBJ2-3 QKLRNSRKTW
39 TRAV17 CATVVRMDSSYKLIFG TRAJ12 TRBV7-9 CASSLVGEGWSDEQFFG TRBJ2-1 TPRVTGGGAM
40 TRAV21 CAVSPNYGQNFVFG TRAJ26 TRBV4-1 CASSQKQAYGYTFG TRBJ1-2 LPEEKQP
41 TRAV13-1 CAASEGGFKTIFG TRAJ9 TRBV11-2 CASSLGTGDNEQYFG TRBJ2-7 RAKFKQLL
42 TRAV17 CATDPRGGSNYKLTFG TRAJ53 TRBV29-1 CSAGSGEWGEQYFG TRBJ2-7 RAKFKQLL
43 TRAV27 CAGVGDSGGSNYKLTFG TRAJ53 TRBV4-1 CASSRLAGDTDTQYFG TRBJ2-3 RAKFKQLL
44 TRAV29/DV5 CVNNQGGKLIFG TRAJ23 TRBV3-1 CASSQARDPISEQYFG TRBJ2-7 RAKFKQLL
45 TRAV34 CGADHASGTYKYIFG TRAJ40 TRBV4-1 CASSPGQGEGYEQYFG TRBJ2-7 RAKFKQLL
