# Supplementary material for: Predicting Antigen‐Specificities of Orphan T Cell Receptors from Cancer Patients with TCRpcDist
Source: Adv Sci (Weinh). 2024 Aug 19;11(40):2405949. doi: 10.1002/advs.202405949 (PMC11516110; doi:10.1002/advs.202405949)
Supplement: Supplementary file 2 — Supporting Information [file ADVS-11-2405949-s001.zip › SI-corrected/SI-webservice/README-TCRpcDist-webservice.pdf]

TCRpcDist – Temporary webservice

```
######################
# On the client side #
######################
```

Checking that the server is up and running

Please type

```
curl -s beta.swiss-tcr.ch:2605
or
curl -s beta.swiss-tcr.ch:2605/
or
curl -s beta.swiss-tcr.ch:2605/hello
```

If the webser is up and running, you receive a "Hello World!" message.

```
> curl -s beta.swiss-tcr.ch:2605
Hello World!
> curl -s beta.swiss-tcr.ch:2605/
Hello World!
> curl -s beta.swiss-tcr.ch:2605/hello
Hello World!
```

TCRpcDist using only CDR3 beta loops

To launch a CDR3 beta distance measure using TCRpcDist, type

```
curl -s -F "myCDR3=@input.txt" beta.swiss-tcr.ch:2605/calccdr3
```

where
- input.txt is the file containing the TCR info, one TCR per line and the CDR3 lenghts for alpha and beta CDR3 have to be higher than or equal to 11 amino acids

An example is attached
The input file contains 8 columns space separated: TCR identifier, TRAV, CDR3a, TRAJ, TRBV, CDR3b, TRBV and peptide recognized (if it is a TCR with unknown specificity you may keep column 8 empty or filled with orphan or NA).
An example is attached with the name input.txt. The first rows of the input.txt are:

```
1 TRAV13-1 CAASDSSASKIIFG TRAJ3 TRBV9 CASSVGKETQYFG TRBJ2-5 FAFGEPREL
2 TRAV14/DV4 CAMREPYYNQGGKLIFG TRAJ23 TRBV4-3 CASSQDRLARDTQYFG TRBJ2-3 FAFGEPREL
3 TRAV26-2 CILRAVYVRFG TRAJ43 TRBV5-5 CASSWKGVYNQPQHFG TRBJ1-5 RRWDEKAVDKSK
```

Before launching, the server will make several checks:

If, for some technical reason, it is impossible to write the file on the server, the server is sending an Error message:

```
> curl -s -F "myCDR3=@input.txt" beta.swiss-tcr.ch:2605/calccdr3
Error: the input file could not be written on our file system. Please,
contact the SwissTCR team.
```

in case of major problem with the machine, we get

```
> curl -s -F "myCDR3=@input.txt" beta.swiss-tcr.ch:2605/calccdr3
internal server error
```

if the input file was empty, the job will not be submitted:

```
> curl -s -F "myCDR3=@input.txt" beta.swiss-tcr.ch:2605/calccdr3
```

Successful submission

If no problem was found , the distance calculation is submitted to the queue of the server. Then, the user is provided with a randomly chosen Session Number.
This Session Number will allow the user to check what is the status of the calculation, and retrieve the results in case of successful calculation.

```
> curl -s -F "myCDR3=@input.txt" beta.swiss-tcr.ch:2605/calccdr3
403049986
```

Here, 403049986 is the Session Number of the submitted screening.

Checking the status of a screening

A user can check the status of a job using the Session Number he/she received upon submission.
If the calculation is pending in the queue, waiting for its turn, the user will be informed, and will be provided with the number of jobs that are waiting before it in the queue.
If the job is currently running, the user will be informed and the running time will be sent.
The user is also informed if the job is terminated

```
> curl -s beta.swiss-tcr.ch:2605/checksession?sessionNumber=403049986
Calculation is in the queue. Number of jobs before yours: 12
```

... and later ...

```
> curl -s beta.swiss-tcr.ch:2605/checksession?sessionNumber=403049986
Calculation is in the queue. Number of jobs before yours: 4
```

... and later ...

```
curl -s beta.swiss-tcr.ch:2605/checksession?sessionNumber=403049986
> Calculation currently running. Run time: 0:02
```

... and later ...

```
> curl -s beta.swiss-tcr.ch:2605/checksession?sessionNumber=403049986
```

```
Calculation is finished
```

On top of the above checking, the system also verifies if the Session Number is correct, and if a problem prevented submitting the calculation for an existing Session

Wrong session number:

```
curl -s beta.swiss-tcr.ch:2605/checksession?sessionNumber=40304998
Error. There is no trace of session 40304998. Is the session number
correct?
```

No job submitted for an existing session:

```
> curl -s beta.swiss-tcr.ch:2605/checksession?sessionNumber=519879130
Error. No calculation was submitted to the queue for session 519879130.
Please resubmit. If failure persists, please, contact the Swiss-TCR team:
vincent.zoete@unil.ch ; marta.perez@sib.swiss
```

Cancelling a screening

The user can cancel a run that is currently running or pending in the queue.
This command will remove the calculation from the queue of the server:

```
curl -s -F "myCDR3=@input.txt" beta.swiss-tcr.ch:2605/calccdr3
174252494
```

... and later ...

```
> curl -s beta.swiss-tcr.ch:2605/cancelsession?sessionNumber=174252494
Calculation of session 174252494 was cancelled
```

Also for this command, the web server checks that the user provided a valid Session Number

```
> curl -s beta.swiss-tcr.ch:2605/cancelsession?sessionNumber=17425249
Error. There is no trace of session 17425249. Is the session number
correct?
```

Retrieving the results of a screening

When a user has check that his/her job is finished (see above), it is possible to retrieve the results.

Here is a full sequence, (i) submitting a job, (ii) regularly checking if the job is finished and (iii) retrieving the results when it is the case:

```
>curl -s -F "myCDR3=@input.txt" beta.swiss-tcr.ch:2605/calccdr3
186742962
```

... and later ...

```
>curl -s beta.swiss-tcr.ch:2605/checksession?sessionNumber=186742962
Calculation is in the queue. Number of jobs before yours: 2
```

... and later ...

```
>curl -s beta.swiss-tcr.ch:2605/checksession?sessionNumber=186742962
Calculation currently running. Run time: 0:01
```

... and later ...

```
> curl -s beta.swiss-tcr.ch:2605/checksession?sessionNumber=186742962
Calculation is finished

> curl -s beta.swiss-tcr.ch:2605/retrievesession?sessionNumber=186742962
> output.out
```

The TCR distances are retrieved to an out

If the user is too impatient, the system can remind him to wait until the job is finished, before retrieving the results

```
> curl -s beta.swiss-tcr.ch:2605/checksession?sessionNumber=437240532
Calculation is in the queue. Number of jobs before yours: 1

> curl -s beta.swiss-tcr.ch:2605/retrievesession?sessionNumber=437240532
Error. Calculation is not finished. Impossible to retrieve the results.
```

Again, the webserver checks the Session Number and the existence of a slurm job, and informs the user in case of problem

```
> curl -s beta.swiss-tcr.ch:2605/retrievesession?sessionNumber=43724053
Error. There is no trace of session 43724053. Is the session number
correct?
```

# TCRpcDist using all the CDRs (alpha and beta chains)

To launch a distance measure considering all the CDRs using TCRpcDist, the use/client must type

```
curl -s -F "myCDRs=@input.txt" beta.swiss-tcr.ch:2605/calcdist
```

All the checks and the retrieve of the results are done as described upwards using the session number

Such a run takes about 20 seconds.

# TCRpcDist-3D: using all the 6 CDRs and solvent accessibility

To launch a TCR distance measure using uniquely the solvent exposed residues of all the six CDRs, the user/client must type this time

```
curl -s -F "myCDRs3D=@input.txt" beta.swiss-tcr.ch:2605/calcdist3D
```

All the checks and the retrieve of the results are done as described upwards using the session number

Such a run takes about 10 minutes.
